# Dissecting Inflammatory Complications in Critically Injured Patients by Within-Patient Gene Expression Changes: A Longitudinal Clinical Genomics Study

**DOI:** 10.1371/journal.pmed.1001093

**Published:** 2011-09-13

**Authors:** Keyur H. Desai, Chuen Seng Tan, Jeffrey T. Leek, Ronald V. Maier, Ronald G. Tompkins, John D. Storey, and the Inflammation and the Host Response to Injury Large-Scale Collaborative Research Program

**Affiliations:** 1 Lewis-Sigler Institute for Integrative Genomics, Princeton University, Princeton, New Jersey, United States of America; 2 Department of Biostatistics, Johns Hopkins Bloomberg School of Public Health, Baltimore, Maryland, United States of America; 3 Department of Surgery, Harborview Medical Center, University of Washington, Seattle, Washington, United States of America; 4 Department of Surgery, Massachusetts General Hospital, Harvard Medical School, Boston, Massachusetts, United States of America; 5 Department of Molecular Biology, Princeton University, Princeton, New Jersey, United States of America; University College London, United Kingdom

## Abstract

**Background:**

Trauma is the number one killer of individuals 1–44 y of age in the United States. The prognosis and treatment of inflammatory complications in critically injured patients continue to be challenging, with a history of failed clinical trials and poorly understood biology. New approaches are therefore needed to improve our ability to diagnose and treat this clinical condition.

**Methods and Findings:**

We conducted a large-scale study on 168 blunt-force trauma patients over 28 d, measuring ∼400 clinical variables and longitudinally profiling leukocyte gene expression with ∼800 microarrays. Marshall MOF (multiple organ failure) clinical score trajectories were first utilized to organize the patients into five categories of increasingly poor outcomes. We then developed an analysis framework modeling early within-patient expression changes to produce a robust characterization of the genomic response to trauma. A quarter of the genome shows early expression changes associated with longer-term post-injury complications, captured by at least five dynamic co-expression modules of functionally related genes. In particular, early down-regulation of MHC-class II genes and up-regulation of p38 MAPK signaling pathway were found to strongly associate with longer-term post-injury complications, providing discrimination among patient outcomes from expression changes during the 40–80 h window post-injury.

**Conclusions:**

The genomic characterization provided here substantially expands the scope by which the molecular response to trauma may be characterized and understood. These results may be instrumental in furthering our understanding of the disease process and identifying potential targets for therapeutic intervention. Additionally, the quantitative approach we have introduced is potentially applicable to future genomics studies of rapidly progressing clinical conditions.

**Trial Registration:**

ClinicalTrials.gov NCT00257231

*Please see later in the article for the Editors' Summary*

## Abstract 2

By studying gene expression changes over time in a cohort of trauma patients, Keyur Desai and colleagues identify genes and pathways strongly associated with longer-term complications, which could lead to improved outcome prediction in the first 80 hours after injury.

## Editors' Summary

**Background:**

Trauma—a serious injury to the body caused by violence or by an accident—is a major global health problem. Every year, events that include traffic collisions, falls, blows, and fires cause injuries that kill more than 5 million people (9% of annual global deaths). Road traffic accidents alone are responsible for 1.3 million deaths a year and, if current trends continue, will be the fifth leading cause of death worldwide by 2030. Moreover, in many countries, including the US, trauma is the number one killer of individuals aged 1–44 y. Trauma can kill people rapidly through loss of blood or serious physical damage to internal organs, but it can also lead to localized infections and to sepsis, an infection of the bloodstream that is characterized by an amplified, body-wide (systemic) inflammatory response. Inflammation—redness, pain, and swelling—is an immune system response that normally provides protection against infections, but systemic inflammation can result in multiple organ failure (MOF) and death.

**Why Was This Study Done?:**

Inflammatory complications of trauma are responsible for more than half of late trauma deaths, but at present it is impossible to predict which patients with major injuries will recover and which will spiral down into MOF and death, because the biological processes that underlie post-injury inflammatory complications are poorly understood. If the changes in gene expression (the process that converts the information encoded in genes into functional proteins) that accompany systemic inflammation could be elucidated, it might be possible to improve the diagnosis of MOF and to develop better treatments for post-trauma inflammatory complications. In this prospective, longitudinal clinical genomics study (part of the Inflammation and Host Response to Injury multi-disciplinary research program [IHRI]), the researchers developed an approach to associate early within-patient gene expression changes with later clinical outcomes. A prospective study is one in which patients with a specific condition are enrolled and then followed to see how various factors affect their outcomes; a longitudinal study analyzes multiple samples taken at different times from individual patients; a clinical genomics study investigates how genes and gene expression affect clinical outcomes.

**What Did the Researchers Do and Find?:**

The researchers followed 168 patients for up to 28 d after they experienced blunt-force trauma (injuries caused when the human body hits or is hit by a large object such as a car). Using a molecular biology tool called a DNA microarray, they determined gene expression patterns in leukocytes (a type of immune system cell) isolated from multiple blood samples collected from each patient during the first few days after injury. Using clinical information collected by trained nurses, they organized the patients into five outcome categories based on a measure of MOF known as the Marshall score. Finally, they developed a statistical method (an analysis framework) to associate the early changes in gene expression with clinical outcomes.

A quarter of the patients' genes showed early expression changes that were associated with longer-term post-injury inflammatory complications. Among the associations revealed by this analysis, down-regulation (reduced expression) of MHC-class II genes (which encode proteins involved in antigen presentation, the process by which molecules from foreign invaders are presented to immune cells to initiate an immune response) and up-regulation of genes encoding components of the p38 MAPK signaling pathway (which helps to drive inflammatory responses) between 40 and 80 h post-injury were particularly strongly associated with longer-term post-injury complications and provided the strongest discrimination between patient outcomes.

**What Do These Findings Mean?:**

The statistical approach used in this study to link the early changes in gene expression that occur after trauma to clinical outcomes provides a detailed picture of genome-wide gene expression responses to trauma. These findings could help scientists understand why some patients develop inflammatory complications of trauma while others do not, and they could help to identify those patients most at risk of developing complications. They could also help to identify targets for therapy, although further studies are needed to confirm and extend these findings. Importantly, the quantitative approach developed by the researchers for analyzing associations between within-patient gene changes over time and clinical outcomes should provide more robust predictions of outcomes than single measurements of gene expression and could be applicable to genomic studies of other rapidly progressing clinical conditions.

**Additional Information:**

Please access these websites via the online version of this summary at http://dx.doi.org/10.1371/journal.pmed.1001093.

- More details about the Inflammation and Host Response to Injury research program are available; the program's website includes a link to an article that explains how genomics can be used to understand the inflammatory complications of trauma
- The World Health Organization provides information on injuries and on violence and injury prevention (in several languages)
- The US National Institutes of Health has a factsheet on burns and traumatic injury in the USA
- The US Centers for Disease Control and Prevention has information on injury and violence prevention and control
- MedlinePlus provides links to further resources on injuries

## Introduction

Trauma is the number one killer of individuals aged 1–44 y [1], a source of some of the top health care costs in the United States [2], and a major global health priority [3],[4]. Trauma injuries frequently lead to infections, sepsis, and multiple organ failure (MOF) [5],[6], which contribute to 51%–61% of late trauma mortality [7]. A number of clinical trials for treating late trauma complications have failed, believed partly due to the inability to identify a proper patient population as well as the limited understanding of the interplay of biological processes underlying post-injury inflammatory complications [8],[9]. A more comprehensive characterization of the genomic response to trauma is therefore required in order to increase our understanding of the molecular basis of clinical outcomes, leading to improvements in diagnosis and treatment.

Despite the public health implications of improved trauma care, relatively few studies have been carried out to understand the molecular basis of trauma recovery, particularly from a genome-wide perspective [10]. An endotoxin experiment on healthy volunteers [11] and a retrospective sepsis study [12] have shown a strong genomic response to trauma-related phenotypes. However, to date there has been no in-depth, prospective longitudinal characterization of the genome-wide expression response to blunt-force trauma that (a) identifies which pathways are fundamental determinants of the patient's recovery trajectory, and (b) elucidates the time period post-injury when these molecular signatures are most informative. Uncovering these factors can reveal new therapeutic strategies and the dynamic regimens for their administration.

To this end, the “Inflammation and the Host Response to Injury” (IHRI) research program conducted a large-scale, 28-d prospective clinical genomics study involving 168 patients, 797 microarrays, and 393 clinical variables. The key statistical challenge we faced was how to accurately associate early longitudinal gene expression measured at multiple time points with 28 d clinical trajectories captured by a constellation of clinical variables. We developed and applied a tractable and robust quantitative framework to analyze this complex clinical genomics study. Specifically, we sought to capitalize on the longitudinal structure within an individual, combining bioinformatics and statistical tools to elucidate pathway dynamics from the gene expression data.

We found that approximately one quarter of the genome changes during early stages of treatment in concordance with the observed variation in 28-d clinical outcomes. These expression changes are coordinated into five distinct modules, which together provide a fine-scale separation of patient outcomes. We pinpointed several pathways that appear to be key drivers of these modules and may be instrumental in furthering our understanding of the disease process and identifying potential targets for therapeutic intervention [13],[14]. We investigated the dynamics of these pathways and found that several discriminate among 28-d post-injury patients trajectories. Specifically, we identified p38 MAPK signaling pathway and MHC-class II genes as having the strongest discrimination in the first 40–80 h. Such information is potentially useful in determining the exact timing and effective dosage of drugs targeting these pathways in trauma patients.

A lack of reproducibility of clinical genomics results [15] has been shown to be largely due to patient heterogeneity, latent sources of confounding, and platform-dependent non-biological variation [16], all difficult to deal with when associating clinical outcome with a single snapshot of gene expression. Taking advantage of the longitudinal design of our study, we developed and applied an approach modeling “within-patient” gene expression dynamics for extracting robust signatures, thereby accounting for patient-specific effects and being more likely to reproduce in future patients. Our framework is likely applicable to other complex clinical genomics studies, especially in rapidly progressing clinical conditions.

## Methods

### Study Design and Patient Samples

**Figure 1:**
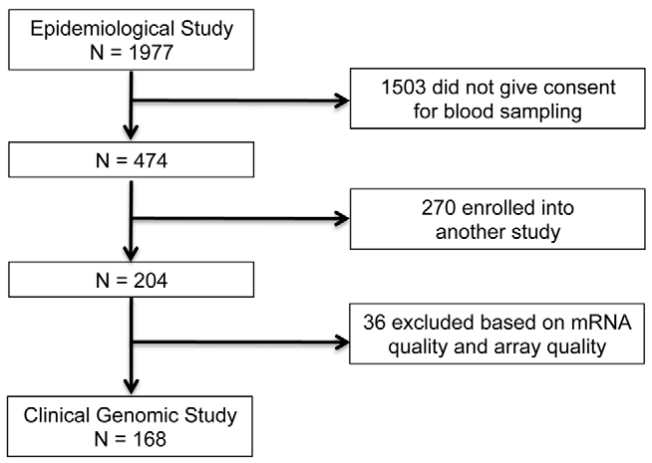
Patient selection for the IHRI study.

In the IHRI prospective clinical genomics study, we studied a cohort of 168 patients (ages 16–55 y; 107 males) from a larger epidemiological study, involving 1977 severe blunt-force trauma patients, conducted from 2003 to 2011 through 7 U.S. Level I trauma centers across the United States. Figure 1 provides the flow chart leading to the 168 patients analyzed in this paper (epidemiological study: ClinicalTrials.gov identifier: NCT00257231). These 168 patients were followed for up to 28 hospital days post-injury, and their longitudinal genome-wide gene expression was measured. To ensure patients were at risk of developing MOF, infectious complications, and death (thereby satisfying the study requirements), the consortium employed a set of inclusion/exclusion enrollment criteria (Text S1). Patients with isolated traumatic brain injury were excluded. Samples were taken at fixed time points following injury according to study design and independent of physician influence. Thus there was no physician or severity of illness bias in the sample collection process.

The institutional review board of each center approved the study, and written informed consent was obtained from all patients or their legal next of kin. The same standardized patient care protocol was used to minimize the impact of variability in clinical care across centers. Patient clinical information, typically consisting of >300 variables (some longitudinal), was collected by trained nurses and entered into a central database to maintain conformity and consistency across all participating centers. For each patient, genome-wide longitudinal gene expression was measured for total blood leukocytes isolated from peripheral blood samples (collection, processing, and normalization described in Text S1 and Figure S1). The data (de-identified as defined by the Health Insurance Portability and Accountability Act of 1996; see http://www.gluegrant.org/trdb.htm) are freely available at http://www.gluegrant.org; see http://www.gluegrant.org/glueadmin/register_consortium.jsp for details.

### Statistical Analysis

**Figure 2:**
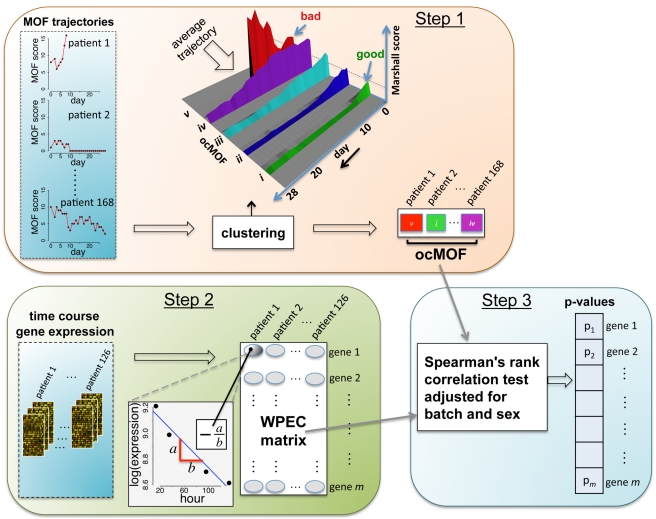
Schematic of the analysis framework. There are three fundamental steps in the analysis framework. Step 1: characterizing phenotypes from longitudinal clinical data; Step 2: quantifying within-patient expression changes from the genomic data; and Step 3: statistical modeling and hypothesis testing to relate the two.

Our proposed statistical framework (Figure 2), derived from two a priori statistical hypotheses (Results), consists of three key steps:

#### Step 1

**Figure 3:**
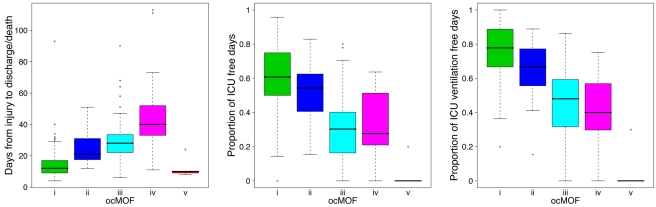
Order of the ocMOF subgroups. The ordering can be determined with the following clinical variables: days from injury to discharge/death, proportion of ICU-free days, and proportion of ICU ventilation–free days. Note that all patients in *ocMOF v* died.

We used a time-dependent modified Marshall score [17] (excluding the neurological component) as the measure of developing MOF, providing an up-to-28-d Marshall score time course trajectory for each patient. We applied hierarchical clustering to the 168 patient Marshall score trajectories, yielding five clusters (Figure 2, Step 1; Text S2). We used relevant patient information, such as the 28-d mortality and morbidity rates, to order these five subgroups (Figure 3; Table 1). This yielded a clinically interpretable, ordered categorical MOF score (ocMOF) ranging from *i* (good outcome) to *v* (bad outcome).

**Table 1:** Summary of the demographic and outcome variables of the 168 patients.

Variables	*ocMOF i*(n = 68)	*ocMOF ii*(n = 32)	*ocMOF iii*(n = 47)	*ocMOF iv*(n = 16)	*ocMOF v*(n = 5)	p-Value
**Demographics**						
Age	31.9±10.7	35.5±12.4	35.9±10.5	34.1±12.3	31.8±6.5	0.1264^a^
Gender Female	28 (41%)	15 (47%)	16 (34%)	2 (12%)	0 (0%)	0.0128^b^
Gender Male	40 (59%)	17 (53%)	31 (66%)	14 (88%)	5 (100%)	
**Design**						
Sampling Phase 1	12 (18%)	10 (31%)	14 (30%)	3 (19%)	3 (60%)	0.0041^b^
Sampling Phase 2	24 (35%)	5 (16%)	9 (19%)	0 (0%)	0 (0%)	
Sampling Phase 3	21 (31%)	8 (25%)	17 (36%)	8 (50%)	2 (40%)	
Sampling Phase 4	11 (16%)	9 (28%)	7 (15%)	5 (31%)	0 (0%)	
**Outcomes**						
Death within 28 d	0 (0%)	0 (0%)	0 (0%)	2 (12%)	5 (100%)	<0.0001^b^
Multiple organ failure	0 (0%)	1 (3%)	30 (64%)	16 (100%)	5 (100%)	<0.0001^b^
Ventilator associated pneumonia	4 (6%)	7 (22%)	27 (57%)	12 (75%)	2 (40%)	<0.0001^b^
Non-infectious complications	8 (12%)	21 (66%)	38 (81%)	15 (94%)	5 (100%)	<0.0001^b^
Surgical site infections	6 (9%)	6 (19%)	14 (30%)	9 (56%)	2 (40%)	<0.0001^b^
Nosocomial infections	15 (22%)	19 (59%)	40 (85%)	15 (94%)	3 (60%)	<0.0001^b^
ICU tracheostomy	1 (1%)	2 (6%)	14 (30%)	5 (31%)	1 (20%)	<0.0001^b^
Days from injury to discharge/death	15.3±12.3	24.6±9.9	31.3±15.2	48.4±28.7	12.2±6.6	<0.0001^a^
Percentage of ICU-free days	60.1±19.7	52.0±18.9	30.9±20.4	30.5±20.9	4.0±8.9	<0.0001^a^
Percentage of ICU ventilator–free days	76.2±17.1	65.2±16.2	45.8±21.1	38.3±23.1	6.0±13.4	<0.0001^a^

For non-categorical variables, the values represent the mean ± standard deviation. For categorical variables, the values represent the total patients and the percentage in the parentheses. To test for association and trend between the various variables and ocMOF as a numerical variable, the Spearman test (^a^) and Deviance test (^b^) of the logistic or multinomial model were used, as appropriate. Order of the ocMOF subgroups could be determined based on the data for the outcome variables.

#### Step 2

For the gene expression analysis, we considered 126 patients with three or more arrays meeting the RNA quality requirements among hours 12–250 (Text S2 and Figure S2). We sought to characterize within-patient expression changes (WPEC) by quantifying per hour log-fold change. To compute WPEC for each probeset, we regressed log gene expression on time (in hours) and extracted the linear slope (Figure 2, Step 2).

#### Step 3

**Figure 4:**
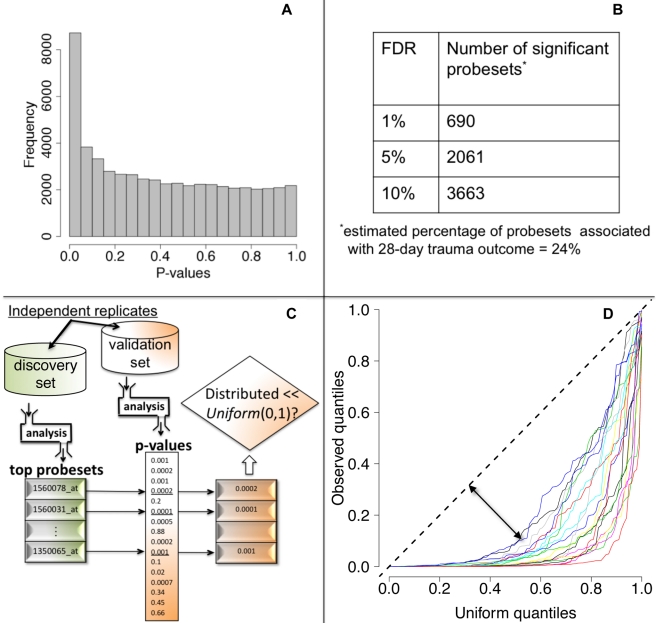
Statistical significance and reproducibility for the IHRIP data. (**a**) The histogram of 54,675 p-values from our framework. (b) The number of significant probesets at various FDR cut-offs. These results indicate strong statistical significance. (**c**) Our strategy to assess reproducibility. (**d**) Reproducibility assessment of our framework: 20 quantile-quantile plots for 20 cross-validations. Consistently large downward deviations from the diagonal (dashed line) indicate reproducibility.

We tested each probeset's WPEC for an association with ocMOF using an adjusted Spearman rank-based correlation test and obtained a p-value for each probeset (Figure 2, Step 3; Text S2). Associating WPEC with ocMOF through a rank-based test enhances robustness, as WPEC values are used to establish an ordering of directional changes across patients, but do not rely on the actual magnitudes. Statistical significance of the 54,675 resulting p-values (Figure 4a) was assessed using the false discovery rate (FDR) [18] yielding an estimate of the total percentage of probesets associated with ocMOF as well as specific probesets identified as significant at various FDR thresholds (Figure 4b).

All the methodological, algorithmic, and data-filtering decisions in Step 1 (clinical data) and in Step 2 (genomic data) were made completely independently by two different analysts to avoid any potential over-fitting.

Additional mathematical and algorithmic details about the statistical methods can be found in Text S2. The R statistical software environment (http://www.r-project.org/) was utilized to perform all data analyses. Computer code that reproduces all results is available as Dataset S1 with detailed documentation provided in Text S2.

### PCA-Based Analysis of WPEC Matrix

To assess the overall effectiveness of WPEC in explaining trauma response, we performed principal component analysis (PCA) [19] on the WPEC matrix and determined the number of principal components (PCs) via the scree plot. We used these PCs as explanatory variables to model each of the 393 measured clinical variables and identified the top ten most significant clinical variables (Text S3).

### Assessment of Reproducibility

We took a principled strategy to assess reproducibility (Figure 4c and 4d). Our strategy (a) obtains the most significant probesets using a “discovery set,” (b) computes their corresponding p-values in a “validation set,” and (c) assesses whether the validation set p-values show systematic, reproducible significance. We complete the last step by comparing the validation set p-values to the *Uniform* (0,1) distribution [18], which corresponds to the distribution of p-values when there is no significance. We performed 20 cross-validations by randomly splitting the study into discovery and validation datasets (Text S3).

### Dominant Expression Trajectories and Dynamic Co-expression Modules

We utilized the DAVID software package to classify probesets with FDR <10% into 54 functionally related gene sets. We further aggregated these gene sets into five modules according to the similarity of their dominant trajectories across the ocMOF subgroups (Text S4). We used the average Pearson correlation of dominant trajectories across ocMOF subgroups as the similarity metric. If the signs of these Pearson correlations were inconsistent across ocMOF subgroups, then the similarity metric was set to zero (no similarity). Next, we used Ward as the agglomeration method and performed hierarchical clustering to obtain five modules.

Specifically, to obtain the five dominant expression trajectories for a gene set, we first removed patient–probeset-specific effects by standardizing the gene expression values (scaling each patient using the sample standard deviation of all of its probesets and separately mean-centering each scaled probeset), and subsequently fitted a loess curve to each ocMOF subgroup. These five dominant trajectories were aligned to a common initial reference.

For each module, the five dominant trajectories were obtained by averaging the corresponding gene sets trajectories (Text S4). We then applied Ingenuity Pathways Analysis (IPA) to identify significant (p-value<0.002, after Bonferroni correction) canonical pathways among the probesets making up each module. The exact settings employed for DAVID and IPA are discussed in Text S4.

## Results

We conducted a large-scale 28-d prospective clinical genomics study involving 168 patients, 797 microarrays, and 393 clinical variables (Methods) in order to understand the molecular basis of clinical responses to trauma from a genome-wide perspective. To this end, the central data-analytic challenge we faced was to associate longitudinal gene expression and 28-d Marshall score time course trajectories without over-fitting the data and while maintaining clinical interpretability. To address this challenge, we developed a within-patient longitudinal gene expression framework (Figure 2), derived from the following a priori statistical hypotheses: (a) there are several distinct trauma recovery trajectories (or physiological responses to trauma), reflected in the time varying clinical measures of interest, and (b) by using each patient as her/his own internal control while modeling the gene expression, inter-patient heterogeneity and confounding are reduced. The framework collapses Marshall score trajectories into clinically interpretable, ocMOF scores and longitudinal gene expression into within-patient expression changes (WPEC). We associated ocMOF and WPEC for each probeset using a rank-based correlation test (Methods).

### Composite Longitudinal ocMOF Score Captures Relevant Clinical Variation

The ocMOF score is designed to capture the clinical variation among patients across the 28-d treatment window (Table 1; Figure 3; Text S5). Note that ocMOF is not introduced here to replace standard clinical measures, but instead serves as a 28-d longitudinal composite of overall patient variation and outcomes in which the higher the score, the worse the patient experience.

The *ocMOF i* subgroup captures uncomplicated recovery with minor or no inflammatory and infectious complications, whereas *ocMOF v* group captures complications leading to MOF and death. Figure 2, Step 1 shows the average Marshall score trajectories for the five ordinal patient subgroups. Note, for example, that the patients with *ocMOF iii* (0% mortality rate) and *v* (100% mortality rate) have very similar Marshall score trajectories during the first 7-d (Figures S3 and S4), and hence are difficult to separate using just Marshall scores (e.g., for the first 7-d mean Marshall scores, the two-sample t-test p-value is 0.506).

### WPEC Measure Robustly Captures Relevant Clinical Variation

Instead of associating absolute expression values with ocMOF, which is the de facto analysis strategy, we instead sought to associate the *within-patient* change in gene expression with clinical outcome. For each probeset and patient, a within-patient expression change (WPEC) was formed by quantifying per-hour log-fold change over hours 12–250 post injury (i.e., regressing log gene expression on time and estimating the linear slope), which was adequate (Text S6 and Figures S5 and S6).

We first performed a PCA-based analysis to assess the overall effectiveness of WPEC in explaining trauma response (Text S6). Eight PCs were obtained from the WPEC matrix (54,675 probesets by 126 patients), capturing 31% of total variation (Figure S7a). Among the 393 clinical variables, those related with Marshall and Denver scores are among the top ten most significant clinical variables associated with these eight PCs collectively, with ocMOF being the most significant (Table S1). We repeated the same analysis on the mean expression matrix (taking the mean across the time course, which effectively combines baseline expression and WPEC) for hours 12–250 (eight PCs, 62% variation, Figure S7b) and found sampling phase and trauma center among the ten most significant variables (Table S2), implying patient-specific baseline expression is susceptible to confounders.

### Significance Analysis of WPEC Associations with 28-d Trauma Outcome

We then performed a test of association between each probeset's WPEC measure and ocMOF to identify probesets that show a statistically significant association with clinical outcome (Methods). Both the resulting p-values (Figure 4a) and the FDR calculations (Figure 4b) indicate strong statistical significance. This information and mean WPEC for each ocMOF subgroup for all 54,675 probesets are provided in Table S3. The estimated percentage of probesets associated [18] with ocMOF was ≥24%, indicating that at least one-quarter of the genome undergoes early within-patient expression changes associated with 28-d trauma outcome.

A lack of reproducible results has been a major hurdle for translational research in clinical genomics [20],[21]. With thousands of genes tested for association and many being involved in the disease, it is important to assess the reproducibility of our association analysis. Therefore, we developed and applied a principled cross-validation strategy to assess reproducibility of significance (Figure 4c). We consistently observed small p-values for the top 100 probesets (Figure 4d), suggesting strong reproducibility of significant associations.

### Dynamic Co-expression Modules Discriminating Trauma Outcome

**Figure 5:**
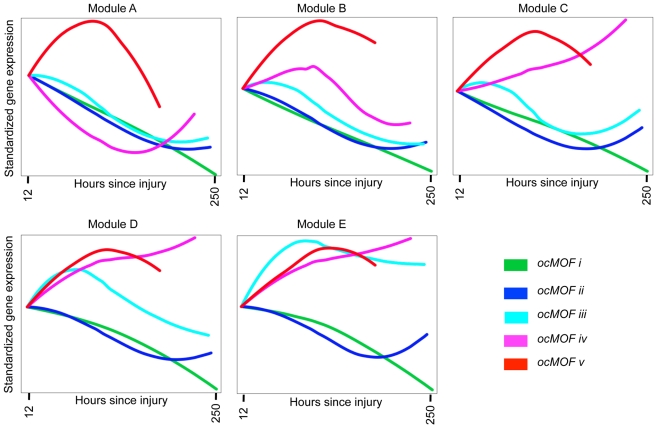
Dynamic co-expression modules and their dominant trajectories across the five ocMOF subgroups. We used DAVID to obtain 54 functionally related gene sets from the 3,663 most significant probesets (10% FDR), which were then clustered into five modules according to the similarity of their dominant trajectories across the ocMOF subgroups. Modules A, B, C, D, and E contain 47, 37, 577, 231, and 364 probesets, respectively. We applied IPA to identify enriched pathways within each module. This IPA analysis shortlisted the following pathways as statistically significant (p-value<0.002, after Bonferroni correction; see Table S5): Oxidative Phosphorylation (Module A); RAN, IL-10 and IL-6 signaling, and the Glycosphingolipid Biosynthesis-Lactoseries Pathway (Module C); Allograft Rejection Signaling, Antigen Presentation Pathway, Cytotoxic T Lymphocyte-mediated Apoptosis of Target Cells, OX40 Signaling Pathway, Nur77 Signaling in T Lymphocytes (Module D); and Protein Ubiquitination Pathway, Hypoxia Signaling, and Cleavage and Polyadenylation of Pre-mRNA in the Cardiovascular System (Module E). Note that Module A contains 47 probesets and one statistically significant pathway, and Module B contains 37 probesets and no statistically significant pathway.

We took a functional genomics approach to characterize the dynamic expression variation driving the WPEC and trauma outcome associations (Methods). We first applied DAVID [22],[23], a state-of-the-art classification algorithm that groups genes based on their co-occurrences in annotation terms, to functionally cluster 1,256 of the 3,663 probesets with FDR <10% (Table S4). We further aggregated these gene sets into five modules according to the similarity of their dominant trajectories across the ocMOF subgroups (Figure S8). These modules were then organized in descending order of their average pairwise similarity metric, Module A (largest) to Module E (smallest); their dominant trajectories are provided in Figures S9, S10, S11, S12, S13. The average dominant trajectory of each ocMOF subgroup in each module shows highly coordinated dynamic co-expression patterns that discriminate the ocMOF groups in an ordered manner (Figure 5), especially in the early time window post-injury.

We then applied IPA, a hand-curated database of biological interactions and functional annotations, to identify statistically significant canonical pathways among the probesets making up each module (Methods). In general, the significant canonical pathways within each module were relevant and related to inflammation and immunity (see Figure 5 caption and Table S5). Module A's dominant trajectory for *ocMOF v* is notably different from the other ocMOF subgroups, and its significant canonical pathway is oxidative phosphorylation. Metabolic dysfunction from trauma and infections has adverse effects on organ systems, and it generally originates from the mitochondrion, which is involved in the metabolism processes through oxidative phosphorylation [24],[25]. For Module E, the most significant canonical pathway is protein ubiquitination; targeting ubiquitin-mediated signaling is thought to regulate nuclear factor-κB (NF-κB), currently of interest as a therapeutic target in inflammatory diseases [26].

### Key Pathways Associated with Trauma Outcome

To identify the key drivers of the genomic response to trauma, we performed ontological analyses on the top 500 most significant probesets using DAVID and IPA. For these 500 probesets, the heatmap of all the gene expression data collected throughout the study (stratified by day and ocMOF outcome) is provided in Figure S14. We sought to identify gene sets that are enriched for biological processes leading to poor trauma outcomes, show tightly coordinated dynamic expression trajectories, and have strong discriminatory power for post-injury MOF.

The top six canonical pathways (p-values<2.2×10^−5^) identified by IPA are dendritic cell maturation, Toll-like receptor (TLR) signaling, p38 mitogen-activated protein kinase (p38 MAPK) signaling, interleukin(IL)-6 signaling, production of nitric oxide and reactive oxygen species in macrophages, and antigen presentation (see Table S6 for the top 20). All six are involved in cellular immune responses, implying a common theme. Two of the pathways, TLR and p38 MAPK signaling, were recently identified in a genome-wide expression study on early sepsis [12]. We applied DAVID to functionally cluster the top 500 probesets (Table S7), yielding five gene groups that strongly support the IPA results. Both analyses identified several genes that have been individually targeted in previous model system studies [27],[28]. We discuss below two gene sets identified from the analyses, with analogous results being shown for three others.

#### Antigen presentation pathway

**Figure 6:**
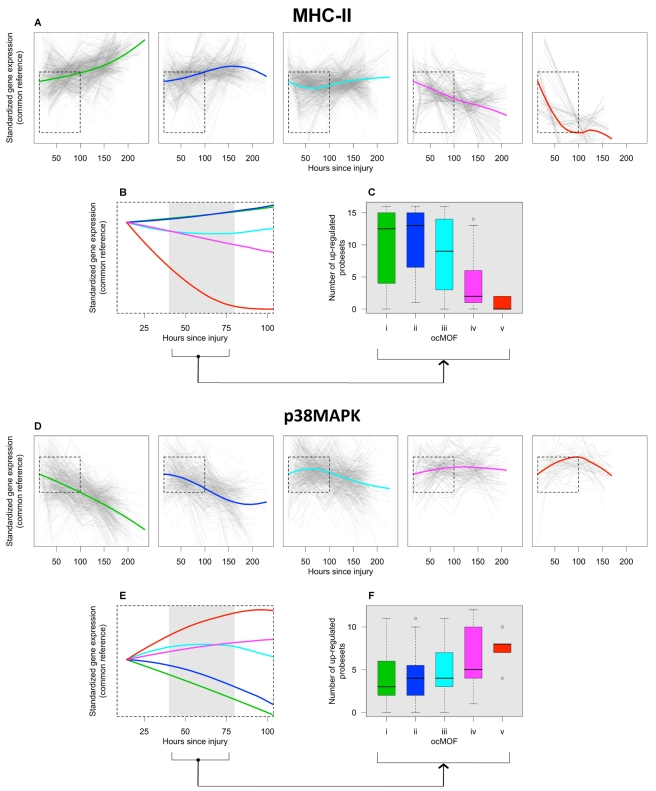
ocMOF and gene expression dynamics of MHC-II and p38 MAPK. For each ocMOF group, the dominant trajectory (thick colored line) was obtained by averaging all the standardized MHC-II (**a**) and p38 MAPK (**d**) probesets trajectories (gray lines) of patients within the ocMOF subgroup. Generally, the dominant trajectory for MHC-II increases with time for *ocMOF i* and *ii*, initially decreases and then increases for *ocMOF iii*, and decreases for *ocMOF iv* and *v*. For p38 MAPK, the early dominant trajectory decreases with time for *ocMOF i* and *ii*, initially increases and then decreases for *ocMOF iii*, and increases for *ocMOF iv* and *v*. The dominant trajectories within the first 100 h suggest that early expression changes (gray region) of MHC-II (**b**) and p38 MAPK (**e**) correlate with patient outcome. The number of up-regulated MHC-II (**c**) and p38 MAPK (**f**) probesets (computed using the two sampling time points closest to the 40–80 h post-injury interval) separates patients with *ocMOF i*, *ii, and iii* from patients with *ocMOF iv* and *v* (p-value of the Kruskal-Wallis test is 0.00004 for MHC-II and 0.00668 for p38 MAPK).

The top gene group from DAVID is enriched with the major histocompatibility complex class II (MHC-II) genes, in which 16 of the 17 probesets in that group are MHC-II, with four being in top 50. One of the top six canonical pathways from IPA is antigen presentation (p-value = 2.2×10^−5^), which also consists of MHC-II genes (Text S7 and Figure S15). The MHC-II molecules are relevant to MOF because they present foreign antigens on the cell surface, which is essential for adaptive or innate immunity [29],[30]. We used the 16 MHC-II probesets (representing HLA-DMB, HLA-DPA1, HLA-DPB1, HLA-DQA1, HLA-DRA, HLA-DRB1, HLA-DRB3, HLA-DRB4, LOC100294318, and LOC100133678 genes) identified by DAVID to comprise the MHC-II gene set for subsequent analysis (Figure 6a–6c).

The boxplots of WPEC suggest that, in moving from *ocMOF i* to *v*, the WPEC decreases for all 16 MHC-II probesets, exhibiting a dosage effect (Figure S16). The dominant expression trajectories also exhibited a similar trend (Figure 6a). The difference among dominant trajectories is particularly pronounced during the 40–80 h window, suggesting that early expression changes can be used to discriminate among the patient ocMOF scores (Figure 6b). Using the two time points closest to 40–80 h post-trauma, we counted the number of up-regulated MHC-II probesets within each patient, and observed consistently high counts for *ocMOF i*, *ii*, and *iii* and low counts for *ocMOF iv* and *v* (Figure 6c).

#### p38 MAPK signaling pathway

All of the top five canonical pathways from IPA contain the mitogen-activated protein kinase 14 (MAPK14) gene, and all four probesets representing it are among the top 500 with three being in top 50. The MAPK14 gene is an isoform of the p38 MAPK gene, the signaling pathway of which (one of the top five canonical pathways, Figure S17) is known to play an important role in driving the inflammatory response to either microbial products (via PAMPs), endogenous danger signals (via DAMPs or alarmins), and pro-inflammatory cytokines by phosphorylating transcription factors, resulting in the further expression of inflammatory mediators [31],[32],[33]. This suggests that the p38 MAPK signaling pathway is an integral signaling mechanism for the other top five canonical pathways and hence the presence of MAPK14 in those pathways. Using IPA, we obtained an additional eight (out of the top 500) probesets representing the genes involved in the p38 MAPK signaling pathway (Text S7), giving us the p38 MAPK gene set (12 probesets, representing MAPK14, CREB5, IL1R1, IL1RN, IRAK2, IRAK3, MAP2K6, and TIFA) for further analysis (Figure 6d–6f).

For the p38 MAPK probesets, we observed a trend opposite that of the MHC-II probesets. Generally, WPEC increased in moving from *ocMOF i* to *v* (Figure S18), and the early dominant trajectories also discriminated the ocMOF groups (Figure 6e–6f). A similar analysis was performed on three other gene sets, representative of the remaining top six canonical pathways, suggesting trends similar to those of p38 MAPK (Text S7 and Figures S19, S20, S21, S22, S23, S24).

Using a controlled endotoxin experiment dataset [11], which served as a corroborative experiment, we obtained dominant trajectories for MHC-II and p38 MAPK among healthy individuals administered with endotoxin (Text S7). The trends of these trajectories from ≥5 h were similar to those seen with *ocMOF i* and *ii* (Figure S25).

## Discussion

In this paper we have provided a comprehensive analysis of the longitudinal IHRIP study, taking into account all major sources of collected data. Despite inherent complexities in clinical genomic data, we showed that robust and relevant genomic signatures can be obtained with our framework, aimed at facilitating straightforward translation into a clinical setting. Our results have implications for the design and analysis of future large-scale clinical genomics studies.

We showed that clinical association using WPEC is straightforward to calculate and appears to be robust to confounders. Our framework collapses Marshall score trajectories and longitudinal gene expression into clinically interpretable quantities, permitting reliable statistical modeling without over-fitting the data. Using our framework we identified genes whose WPEC discriminate among the ocMOF outcomes. One of the main advantages of utilizing WPEC is that it leads more directly than do other measures to a clinical translation of the results, because it captures the change in expression *within* a patient, regardless of the patient's baseline value, which is susceptible to patient heterogeneity, confounders, and technical effects. On the other hand, any snapshot, baseline, or average expression profile will be susceptible to these effects. We repeated the above analyses using both an estimate of the hour 12 expression value and the average over the entire time course. Both measures showed evidence of being influenced by confounders (particularly batch and trauma center effects), and neither produced biological significance greater than WPEC.

We performed a global functional genomics analysis of the top 3,663 statistically significant WPEC associations with trauma outcome (FDR = 10%), identifying five dynamic co-expression modules highly enriched for immune pathways. We also performed a more focused pathway analysis of the top 500 associations (FDR = 0.6%) and identified a number of relevant gene sets. We pinpointed the MHC-II and p38 MAPK gene sets, showing that their expression dynamics suggest their potential as biomarkers. Moreover, our analysis suggests that the strongest discrimination occurs in the first 40–80 h post-injury.

From the dynamic co-expression modules results, one can consider the configuration of ocMOF-specific trajectories within and among these modules along with the biological significance of the modules to construct a spectrum of biologically relevant gene expression variation discriminating the clinical outcomes (ocMOF *i* to *v*). For example, our module-based analysis pointed to the NF-κB pathway. Previous studies have indicated that this pathway, which is downstream of the TLR, is critical in the context of post-traumatic immune dysfunction-induced poor outcomes [34],[35]. Taken as a whole, this systems analysis revealed a large and coordinated gene expression response to trauma, characterized by the modules and gene sets, indicating that something is to be gained from a systems-level understanding [36] of the molecular biology of post-injury MOF in forming therapeutic targets and prognostic procedures.

Our findings on the down-regulation of MHC-II genes among patients from *ocMOF iv* to *v* are consistent with persistently low HLA-DR expression that has been associated with septic complications [37],[38], because a marked depression of cell-mediated immune function (i.e., immunosuppression) is believed to play a role in sepsis after severe trauma. HLA-DR is a promising molecular surrogate marker for treating post-injury inflammatory complications [39], and monitoring HLA-DR expression to treat trauma patients with immunomodulatory drugs such as interferon-γ has been studied. Importantly, our genome-wide approach suggests the association of the entire MHC-II gene set (represented by 16 probesets, Kruskal-Wallis p-value = 0.00004, Figure 6c), besides HLA-DR (represented by five of these 16 probesets, Kruskal-Wallis p-value = 0.00029), may be more informative. Persistent systemic inflammatory response syndrome (SIRS) is shown to be predictive of nosocomial infection in trauma patients [40],[41], which is consistent with the up-regulation of genes in the p38 MAPK signaling pathway among patients from *ocMOF iii* to *v*.

It has been posited that MOF is an outcome of an inappropriate generalized inflammatory response, involving the interplay between mediators (e.g., cytokines and chemokines) and effector cells (e.g., neutrophils and macrophages) [42]. The SIRS and compensatory response syndrome (CARS) are proposed to be involved in the etiology of MOF [7]. It may well be that the increase in p38 MAPK expression seen here after severe trauma reflects the development of SIRS while a persistent decrease in MHC-II expression reflects the development of CARS (Text S8), which is supported from our agnostic, genome-wide analysis. Beyond the definitions of CARS and SIRS, the modern concept of post-traumatic immune suppression is also believed to be a major cause of secondary infections or organ dysfunction [43],[44].

Although it was to date the largest clinical genomic study on trauma response of which we are aware, the current study has certain limitations. Since gene expression was measured for total blood leukocytes isolated from peripheral blood samples, some of the differential expression changes identified could be confounded by changes in individual leukocyte subpopulations. Arguably, the predictive utility of the identified biomarkers still exists.

In this study, we identified relevant pathways and gene sets showing a coordinated pattern of expression variation associated with response to trauma at a genome-wide scale. These findings potentially provide the most comprehensive picture of the gene expression response to trauma to date, thereby demonstrating the power of moving beyond candidate gene studies [45] of this clinical condition. The expression variation at the genomic level that we have characterized among patients may help to provide a more comprehensive set of drug targets and a means to identify relevant subsets of patients for which these may be effective.

## Supporting Information

Dataset S1 **Annotated scripts that reproduce the results in the paper.** The scripts run the entire analysis in R statistical software (cran.r-project.org). See Text S2 for the details and http://genomine.org/trauma/ for instructions on obtaining the full dataset.
(ZIP)

Figure S1 **Microarray collection time points by patient.** X-axis is the time from injury and Y-axis patient IDs. Each circle represents a microarray collected. Intended sampling was on days 0, 1, 4, 7, 14, 21, and 28 since injury, but depending on the total days from injury to discharge/death, the number of microarrays per patient ranged between 2 to 7.
(PDF)

Figure S2 **Heatmap of patient–patient correlations.** Using the WPEC matrix we computed patient–patient correlations for 129 patients. The heatmap of dichotomized correlations (black = negative; gray = positive) identified two patients as outliers with completely opposite correlations from the rest. We removed these two patients due to potential array quality issues.
(PDF)

Figure S3 **Heatmap of the modified Marshall scores on day 0, 2, 3, …, 20 and the dendrogram of the hierarchical clustering.** Hierarchical clustering was performed on the modified Marshall score trajectories, where missing scores were imputed using k-nearest neighbor. The left plot is the dendrogram of the hierarchical clustering from which we obtained five subgroups: *ocMOF i* to *v*. Patients from *ocMOF i* to *iii* tend to have low modified Marshall scores, with patients with *ocMOF i* recovering to 0 first, followed by *ocMOF ii* and *iii*, while patients from *ocMOF iv* and *v* tend to have high modified Marshall scores throughout the first 20 d.
(PDF)

Figure S4 **Marshall score trajectories and ocMOF.** Thin dashed lines in gray correspond to patient-specific Marshall score trajectories, and thick solid lines to the mean trajectories of the ocMOF subgroup. Only the observed modified Marshall scores are used to make these plots, but the actual clustering was performed on imputed data. Note that four out of five patients with *ocMOF v* died on or before day 10 post-injury, and that the red dashed line is for the remaining patient who died on day 24 post-injury. Mean ocMOF trajectories, together with other relevant patient clinical information, allowed us to order the ocMOF clusters in terms of overall patient severity. In particular, *ocMOF i* = good outcome (fast and uncomplicated recovery) and *ocMOF v* = very bad outcome (death).
(PDF)

Figure S5 **Probesets with different dynamics.** (**a–d**) Expression trajectory of probesets in the time window 0–250 h (shown in **a** and **c**) and 12–250 hours (shown in **b** and **d**). Patient-specific trajectories are represented by gray lines, and population average trajectories and population average linear trajectories are represented by the black and red lines respectively. (**a**) and (**b**) correspond to the most non-significant probeset from the DWPEC analysis, where the differences between the average trajectory and average linear trajectory are minimal. (**c**) and (**d**) correspond to the most significant probeset from the DWPEC analysis, where the differences between the average trajectory and average linear trajectory are large in time window 0–250 h but are reduced in time window 12–250 h.
(PDF)

Figure S6 **Reasoning to exclude hours <12.** Boxplots of mean-square difference (MSD) between the population average trajectory and average linear trajectory for the 5,000 most non-significant (ns) and significant (sig) probesets from the DWPEC analysis. We investigated the MSD for four different time windows: hour 0–250, hour 4–250, hour 8–250 and hour 12–250. The MSDs for the non-significant probesets are very similar across all four time windows, but the MSDs for the significant probesets are generally high for hour 0–250 and tend to decrease as we progressively exclude the early hours.
(PDF)

Figure S7 **The scree plots to determine the number of principal components.** (**a**) corresponds to the WPEC matrix and (**b**) to the mean expression matrix.
(PDF)

Figure S8 **The dendrogram for grouping the 54 functional related gene sets into five modules according to the similarity of their dominant trajectories across the ocMOF subgroups.**
(PDF)

Figure S9 **The dominant trajectories for Module A.** For each ocMOF subgroup, where (**a**)–(**e**) correspond to *ocMOF i* to *v*, the dominant trajectories of the module (thick colored lines) are obtained by averaging all dominant trajectories of gene sets belonging to the module (gray lines). (**f**) plots all five ocMOF subgroup dominant trajectories for Module A in one plot by aligning them to a common initial reference.
(PDF)

Figure S10 **The dominant trajectories for Module B.** See the caption for Figure S9 for details.
(PDF)

Figure S11 **The dominant trajectories for Module C.** See the caption for Figure S9 for details.
(PDF)

Figure S12 **The dominant trajectories for Module D.** See the caption for Figure S9 for details.
(PDF)

Figure S13 **The dominant trajectories for Module E.** See the caption for Figure S9 for details.
(PDF)

Figure S14 **The heatmap of ranked gene expressions for all 168 patients over 28 d for the 500 most significant probesets from our analysis.** For each probeset, we ranked the expression values across all 168 patients over 28 d, i.e. 797 microarrays (green = low rank, black = average rank, red = high rank). The columns are microarrays ordered by days, and within each day by ocMOF values. The intended sampling was on days 0, 1, 4, 7, 14, 21, and 28 since injury.
(PDF)

Figure S15 **The antigen presentation pathway.** The MHC-II genes have negative spearman correlation coefficients between WPEC and ocMOF (colored blue).
(PDF)

Figure S16 **ocMOF and gene expression dynamics of MHC-II.** (**a**) Boxplots of WPEC versus ocMOF indicate a negative dosage effect as we go from *ocMOF i* to *v* (p-value of the Spearman's test <10^−15^). (**b**) For each ocMOF group, the dominant trajectory (thick colored line) was obtained by averaging all the standardized MHC-II probeset trajectories (gray lines) of patients within the ocMOF subgroup. Generally, the dominant trajectory increases with time for *ocMOF i* and *ii*, initially decreases and then increases for *ocMOF iii*, and decreases for *ocMOF iv* and *v*. Both WPEC and the dominant trajectories exhibit similar trend. (**c**) The dominant trajectories within the first 100 h suggest that early expression changes (gray region) correlate with patient outcome. (**d**) The number of up-regulated MHC-II probesets (computed using two time points near hour 40–80) separates patients with *ocMOF i*, *ii*, and *iii* from patients with *ocMOF iv* and *v* (p-value of the Kruskal-Wallis test is 0.00004).
(PDF)

Figure S17 **The p38 MAPK signaling pathway.** Among the top 500 probesets, 15 are in this canonical pathway (representing 11 genes). Those genes in blue and red have negative and positive Spearman correlation coefficients between WPEC and ocMOF, respectively. TRADD, MEF2, and Max were removed from further analysis because their correlations were inconsistent with those identified by IPA.
(PDF)

Figure S18 **Gene expression profiles of the 12 probesets involved in the p38 MAPK signaling pathway.** (**a**) Boxplots of WPEC versus ocMOF indicate a positive dosage effect as we go from *ocMOF i* to *v* (p-value of the Spearman's test <10^−15^). (**b**) For each ocMOF group, the dominant trajectory (thick colored line) was obtained by averaging all the standardized p38 MAPK probeset trajectories (gray dotted lines) of patients within the ocMOF subgroup. Generally, the early dominant trajectory decreases with time for *ocMOF i* and *ii*, initially increases and then decreases for *ocMOF iii*, and increases for *ocMOF iv* and *v*. Both WPEC and the dominant trajectories exhibit similar trends. (**c**) The dominant trajectories within the first 100 h suggest that early expression changes (gray region) correlate with patient outcome. (**d**) The number of up-regulated p38 MAPK probesets (computed using two time points near hour 40–80) separates patients with *ocMOF i*, *ii, and iii* from patients with *ocMOF iv* and *v* (p-value of the Kruskal-Wallis test is 0.00668).
(PDF)

Figure S19 **The Toll-like receptor (TLR) pathway.** Among the top 500 probesets, 12 are in this canonical pathway (representing nine genes). Those genes in blue and red have negative and positive Spearman correlation coefficients between WPEC and ocMOF, respectively. JNK1 was removed from further analysis because its correlation was inconsistent with that identified by IPA.
(PDF)

Figure S20 **Gene expression profiles of probesets involved in the TLR pathway.** Similar to p38 MAPK signaling pathway. See Figure S18 for details. Altogether 11 probesets (representing eight genes) were used for this pathway. For (**a**) the p-value of the Spearman's test <10^−15^ and for (**d**) the p-value of the Kruskal-Wallis test is 0.02092.
(PDF)

Figure S21 **The Interleukin (IL)-6 signaling pathway.** Among the top 500 probesets, 14 are in this canonical pathway (representing ten genes). Genes in blue and red have negative and positive Spearman correlation coefficients between WPEC and ocMOF, respectively. JNK was removed from further analysis because its correlation was inconsistent with that identified by IPA.
(PDF)

Figure S22 **Gene expression profiles of probesets involved in the IL-6 signaling pathway.** Similar to p38 MAPK signaling pathway. See Figure S18 for details. Altogether 13 probesets (representing nine genes) were used for this pathway. For (**a**) the p-value of the Spearman's test <10^−15^ and for (**d**) the p-value of the Kruskal-Wallis test is 0.00898.
(PDF)

Figure S23 **The production of nitric oxide and reactive oxygen species in macrophages pathway.** Among the top 500 probesets, 18 are in this canonical pathway (representing 13 genes). Genes in blue and red have negative and positive Spearman correlation coefficients between WPEC and ocMOF, respectively. JNK and PP1/PP2a were removed from further analysis because their correlations were inconsistent with those identified by IPA. IkB and p38 MAPK were removed because of their complexity in this pathway.
(PDF)

Figure S24 **Gene expression profiles of probesets involved in the production of nitric oxide and reactive oxygen species in macrophages pathway.** Similar to p38 MAPK signaling pathway. See Figure S18 for details. Altogether 11 probesets (representing nine genes) were used for this pathway. For (**a**) the p-value of the Spearman's test <10^−15^ and for (**d**) the p-value of the Kruskal-Wallis test is 0.01370.
(PDF)

Figure S25 **Gene expression profiles of MHC-II and p38 MAPK in a controlled endotoxin experiment.** (**a,b**) The mean of the log-expression of MHC-II (**a**) and p38 MAPK (**b**). The black and red lines correspond to the healthy patients administered with placebo and endotoxin, respectively. After hour 5 (the region to the right of the dotted vertical line), the mean trajectories corresponding to healthy patients administered with endotoxin are similar to the dominant trajectories of *ocMOF i* and *ii*.
(PDF)

Table S1 **The ten most significant clinical variables (out of 393) associated with the eight principal components from the WPEC matrix.** Legend: *Clinical variables are treated as categorical variables, and R^2^ corresponds to McFadden's pseudo R^2^.
(PDF)

Table S2 **The ten most significant clinical variables (out of 393) associated with the eight principal components from mean expression matrix.** Legend: *Clinical variables are treated as categorical variables, and R^2^ corresponds to McFadden's pseudo R^2^.
(PDF)

Table S3 **Ranking of the 54,675 probesets according to the significance analysis of WPEC associations with 28-d trauma outcome.**
(XLS)

Table S4 **Fifty-four functional related gene sets from DAVID for the 3,663 significant (FDR = 10%) probesets.**
(XLS)

Table S5 **The top five canonical statistically significant (p-value<0.002, after Bonferroni correction) pathways for the five dynamic co-expression modules.** For each canonical pathway we report the p-value of Fisher's exact test that ascertains enrichment and the proportion of genes in the pathway that were actually in the module within the brackets, and the gene names (in italics). Note that N.A. denotes no significant pathways with three or more genes were identified.
(PDF)

Table S6 **The top 20 canonical pathways for the top 500 probesets from WPEC and ocMOF association analysis (IPA, obtained May 2010).** Legend: p, p-value of Fisher's exact test for ascertaining enrichment; and ratio, the proportion of genes in the pathway that are in the top 500.
(PDF)

Table S7 **Detailed results of DAVID analysis on the top 500 probesets from WPEC and ocMOF association analysis.**
(PDF)

Text S1 Inclusion/exclusion criteria and gene expression information.
(PDF)

Text S2 Additional details on statistical framework.
(PDF)

Text S3 Details on assessing WPEC robustness and reproducibility.
(PDF)

Text S4 Details on module, pathway, and gene set analysis.
(PDF)

Text S5 Marshall MOF-derived clinical outcomes.
(PDF)

Text S6 Results on assessing WPEC robustness.
(PDF)

Text S7 Key pathways associated with trauma outcomes.
(PDF)

Text S8 p38 MAPK and MHC-II as biomarkers for SIRS and CARS.
(PDF)

## Abbreviations

CARS: compensatory response syndrome
FDR: false discovery rate
ocMOF: ordered categorical MOF score
IHRI: Inflammation and the Host Response to Injury
IPA: Ingenuity Pathways Analysis
MOF: multiple organ failure
PCA: principal component analysis
SIRS: systemic inflammatory response syndrome
WPEC: within-patient expression changes
