# Supplementary material for: Dissecting Inflammatory Complications in Critically Injured Patients by Within-Patient Gene Expression Changes: A Longitudinal Clinical Genomics Study
Source: PLoS Med. 2011 Sep 13;8(9):e1001093. doi: 10.1371/journal.pmed.1001093 (PMC3172280; doi:10.1371/journal.pmed.1001093)
Supplement: Dataset S1 — Annotated scripts that reproduce the results in the paper. The scripts run the entire analysis in R statistical software (cran.r-project.org). See Text S2 for the details and http://genomine.org/trauma/ for instructions on obtaining the full dataset. (ZIP) [file pmed.1001093.s001.zip › code/2_normalization/README.rtf]

The main *.R files for this subfolder:1. DChipnormalization.RPurpose: To normalize microarrays by calling dChip from R 2. Splitdata.RPurpose: To generate 20 sets/splits of train and test data for cross-validation 3. ComputeWPEC.RPurpose: Get slope (method=0), intercept (method=1), mean (method=2), of longitudinal gene expressions for various time intervals4. Consolidatebatches.RPurpose: Consolidate expression data from the four separate batches. The subroutine *.R files for this subfolder:1. gen.dChip.ini.RPurpose: A function that calls dChip to normalize the microarrays (i.e. *.CEL files) 2. mtx_linfit.RPurpose: Compute slope and offset (intercept) via matrix computation. 
