# Supplementary material for: Dissecting Inflammatory Complications in Critically Injured Patients by Within-Patient Gene Expression Changes: A Longitudinal Clinical Genomics Study
Source: PLoS Med. 2011 Sep 13;8(9):e1001093. doi: 10.1371/journal.pmed.1001093 (PMC3172280; doi:10.1371/journal.pmed.1001093)
Supplement: Dataset S1 — Annotated scripts that reproduce the results in the paper. The scripts run the entire analysis in R statistical software (cran.r-project.org). See Text S2 for the details and http://genomine.org/trauma/ for instructions on obtaining the full dataset. (ZIP) [file pmed.1001093.s001.zip › code/3_analysis/README.rtf]

The main *.R files for this subfolder:1. Adjanalysis.RPurpose: To generate the adjusted Spearman analysis using 126 patients         Produce the figure in the main paper.         Main Figure 4aThe subroutine *.R files for this subfolder:1. analysisfunctions.R Purpose: Contain functions for performing adjusted analysis
