# Supplementary material for: Dissecting Inflammatory Complications in Critically Injured Patients by Within-Patient Gene Expression Changes: A Longitudinal Clinical Genomics Study
Source: PLoS Med. 2011 Sep 13;8(9):e1001093. doi: 10.1371/journal.pmed.1001093 (PMC3172280; doi:10.1371/journal.pmed.1001093)
Supplement: Dataset S1 — Annotated scripts that reproduce the results in the paper. The scripts run the entire analysis in R statistical software (cran.r-project.org). See Text S2 for the details and http://genomine.org/trauma/ for instructions on obtaining the full dataset. (ZIP) [file pmed.1001093.s001.zip › code/5_genesets/README.rtf]

The main *.R files in this subfolder:1. Array_days.RPurpose: To identify patients whose arrays were burnt on different days.2. ModuleTop3663.RPurpose: Module approach for the top 3663 probesets from the adjusted Spearman analysis using 126 patients.         Produce the figure in the Supplementary Appendix.         Supplementary Figure 8 to 133. Probesets_genesets.RPurpose: To get the probesets for top gene sets.4. Dominant_trajectory.RPurpose: To get the dominant trajectories of MHC-II and p38MAPK         Produce the figure in the Main paper.         Main Figure 65. Boxplot_WPEC.RPurpose: To get the boxplot of slopes, dominant trajectories and counts of up/down for the various gene sets         Produce the figure in the Supplementary Appendix.         Supplementary Figure 16, 18, 20, 22 and 246. Endotoxin_analysis.RPurpose: Plot the mean log-expression of MHC-II and p38MAPK gene sets with endotoxin data          Produce the figure in the Supplementary Appendix.         Supplementary Figure 25The subroutine *.R files for this subfolder:1. slope_trajectory_count.R Purpose: To get the boxplot of slopes, dominant trajectories and up/down counts
