# Supplementary material for: Dissecting Inflammatory Complications in Critically Injured Patients by Within-Patient Gene Expression Changes: A Longitudinal Clinical Genomics Study
Source: PLoS Med. 2011 Sep 13;8(9):e1001093. doi: 10.1371/journal.pmed.1001093 (PMC3172280; doi:10.1371/journal.pmed.1001093)
Supplement: Dataset S1 — Annotated scripts that reproduce the results in the paper. The scripts run the entire analysis in R statistical software (cran.r-project.org). See Text S2 for the details and http://genomine.org/trauma/ for instructions on obtaining the full dataset. (ZIP) [file pmed.1001093.s001.zip › code/6_assessing_data/README.rtf]

The main *.R files for this subfolder:1. ClinInfoTable.RPurpose: To get clinical characteristics of the ocMOF subgroups         Extract the information for the table in the main paper2. HeatmapAll.RPurpose: Plot the heatmap of all 168 patients from hour 0 to 800         Produce the figure in the Supplementary Appendix.         Supplementary Figure 143. DataQualArrayTime.RPurpose: Data quality issues and microarray collection for all patients         Produce the figure in the Supplementary Appendix.         Supplementary Figure 1 and 24. First12hour.RPurpose: Reasons for excluding the first 12 hours gene expression         Produce the figure in the Supplementary Appendix.         Supplementary Figure 6 and 55. ReadClinVar.RPurpose: To get all the clinical variables into one *.Rdata file6. CleanClinVar.RPurpose: To clean up the clinical data and keep the first 6 days7. PCAarrayClinVarWPEC.RPurpose: Regresses the clinical variable onto the principal components of WPEC         Produce the figure in the Supplementary Appendix.         Supplementary Figure 7a         Extract the information for the table in the Supplementary Appendix.         Supplementary Table 18. PCAarrayClinVarMean.RPurpose: Regresses the clinical variable onto the principal components of mean expression         Produce the figure in the Supplementary Appendix.         Supplementary Figure 7b         Extract the information for the table in the Supplementary Appendix.         Supplementary Table 29. CorMHC2WPEC.RPurpose: Identifying probesets with similar WPEC profile as MHC2 gene sets10. HLA-DR.RPurpose: Compute the Kruskal-Wallis tests for the HLA-DR probesets only within the MHC2 MHC2 gene setsThe subroutine *.R files for this subfolder:1. pcaArrayClinVar_subroutine.R Purpose: Regresses the clinical variable onto the principal components coming from either WPEC/mean expression.
