# Supplementary material for: Dissecting Inflammatory Complications in Critically Injured Patients by Within-Patient Gene Expression Changes: A Longitudinal Clinical Genomics Study
Source: PLoS Med. 2011 Sep 13;8(9):e1001093. doi: 10.1371/journal.pmed.1001093 (PMC3172280; doi:10.1371/journal.pmed.1001093)
Supplement: Dataset S1 — Annotated scripts that reproduce the results in the paper. The scripts run the entire analysis in R statistical software (cran.r-project.org). See Text S2 for the details and http://genomine.org/trauma/ for instructions on obtaining the full dataset. (ZIP) [file pmed.1001093.s001.zip › code/README.rtf]

The folder contains the *.R files which perform the analysis and generate the figures/tables presented in the paper. The main.R file is the master script and this is its workflow:  (1) Get the ordered categorical MOF (ocMOF)                                 (2) Normalize the microarrays separately according to batch with dchip and compute WPEC(3) Perform adjusted Spearman analysis with WPEC and ocMOF(4) Perform reproducibility analysis using 20 cross-validations(5) Investigate the modules and gene sets discussed in the paper(6) Assessing the microarrays in the GLUE dataThe main.R file creates the following subfolders which contains information related to the analysis:1. logContains the *.log files from running main.R. The information_paper.log contains information from the analysis which is either mentioned or presented as a table in the paper or supplementary material. The main.log (or main_bypass.log) contains the R console output.2. plots/forpaperContains the figures used in the paper and supplementary material.3. tablesContains the large tables presented in the paper or supplementary material.It calls the *.R scripts in the various subfolders. The subfolders are presented according to the order of the workflow of main.R:1_ocMOFContains the *.R files for obtaining the ordered categorical MOF (ocMOF).2_normalizationContains the *.R files for:i.   normalizing the microarrays with chip.ii.  consolidating the normalize microarrays across batches.iii. computing the slope, offset and mean gene expressions.3_analysisContains the *.R files for performing the adjusted Spearman analysis.4_reprod_20cvContains the *.R files for performing reproducibility analysis.5_genesetsContains the *.R files for:i    plotting the dominant trajectories of the modules with 3663 probesets.ii.  plotting the dominant trajectories of the gene sets (including the endotoxin data) and the boxplots.6_assessing_dataContains the *.R files for:i.   getting the information for the clinical characteristics of the ocMOF sub-groups.ii.  plotting the heatmap of the gene expression from all 797 microarrays. iii. assessing the data quality issue and microarray collection for all patients.iv.  the reasoning to exclude the first 12 hours of gene expressions.v.   assessing the expression data variation (WPEC, mean expression) with PCA with the various clinical various.vi.  identifying probesets with similar WPEC profile as MHC2 gene set.vii. identifying HLA-DR probesets that are subset of the MHC2 gene set.
