# Supplementary material for: Dissecting Inflammatory Complications in Critically Injured Patients by Within-Patient Gene Expression Changes: A Longitudinal Clinical Genomics Study
Source: PLoS Med. 2011 Sep 13;8(9):e1001093. doi: 10.1371/journal.pmed.1001093 (PMC3172280; doi:10.1371/journal.pmed.1001093)
Supplement: Dataset S1 — Annotated scripts that reproduce the results in the paper. The scripts run the entire analysis in R statistical software (cran.r-project.org). See Text S2 for the details and http://genomine.org/trauma/ for instructions on obtaining the full dataset. (ZIP) [file pmed.1001093.s001.zip › data/README.rtf]

Contains the data used in the paper: microarray data and clinical data with their annotation files. All of the data are freely available at www.gluegrant.org for registered researchers. "Members who seek access to the human research data will have been granted from their home institution, institutional review board (IRB) approval to receive human research data in a manner consistent with the protection of confidentiality of the subjects" (www.gluegrant.org/glueadmin/register_consortium.jsp). Please contact the corresponding author John Storey (jstorey@princeton.edu) after being granted access to the full data set, and he will provide explicit instructions for incorporating the raw clinical and expression data into this analysis workflow bundle.The user will need to obtain the dChip normalization software from https://sites.google.com/site/dchipsoft/ (we used dchip_2010_01.exe). Rename this file to dchip.exe (drop the version info) and copy this .exe to CEL folder.  The folder contains data used in the paper and the subfolders are:1. CELContains the files for normalizing the microarrays and mapping the microarrays id/file name to patient id.2. normarrayContains the files of the normalized microarrays and the *.txt of the 20 cross-validations used in the paper.3. clinical_dataContains the files with clinical information of patients.4. endotoxinContains the normalized gene expression of the endotoxin study of Calvano SE et al (2005).5. IngenuityContains files from Ingenuity with information of the ranking of canonical pathways Ingenuity and mapping of the top 500 probesets from the WPEC analysis of 126 patients (12-250hours).6. top3663WPECContains file from DAVID with information of the gene sets of the top 3663 probesets from the WPEC analysis of 126 patients (12-250hours).
