# Supplementary material for: Dissecting Inflammatory Complications in Critically Injured Patients by Within-Patient Gene Expression Changes: A Longitudinal Clinical Genomics Study
Source: PLoS Med. 2011 Sep 13;8(9):e1001093. doi: 10.1371/journal.pmed.1001093 (PMC3172280; doi:10.1371/journal.pmed.1001093)
Supplement: Dataset S1 — Annotated scripts that reproduce the results in the paper. The scripts run the entire analysis in R statistical software (cran.r-project.org). See Text S2 for the details and http://genomine.org/trauma/ for instructions on obtaining the full dataset. (ZIP) [file pmed.1001093.s001.zip › documentation/Information.rtf]

Purpose: Information.rtf gives an overall perspective of the directory and analysis. The README.rtf in each subfolder contains detailed description of its contents.Prepared by: Chuen Seng Tan/John Storey/Keyur DesaiEmail: chuentan@princeton.edu/jstorey@princeton.edu/kdesai@princeton.edu Section 1: Structure of the working directoryThe working directory contains the following folders:1. codeIt contains a main.R, which runs the entire analysis and generates all the figures/tables in the paper by calling *.R scripts in its subfolders. The workflow of main.R:(1) Get the ordered categorical MOF (ocMOF)(2) Normalize the microarrays separately according to batch with dchip and compute WPEC(3) Perform adjusted Spearman analysis with WPEC and ocMOF(4) Perform reproducibility analysis using 20 cross-validations(5) Investigate the modules and gene sets discussed in the paper(6) Assessing the microarrays in the GLUE data The main.R file creates the following subfolders that contain information related to the analysis from the running of the scripts: (1) log: contains the *.log files from running main.R. The information_paper.log contains information from the analysis which is either mentioned or presented as a table in the paper or supplementary material. The main.log contains the R console output.(2) plots/forpaper: contains the figures used in the paper and supplementary material.(3) tables: contains the large tables presented in the paper or supplementary material.2. dataIt contains the data used in the paper: microarray data (CEL, normarray, endotoxin, Ingenuity & top3663WPEC subfolders) and clinical data with their annotation files (clinical_data subfolder).Note that the user will need to obtain the dChip normalization software from https://sites.google.com/site/dchipsoft/ (we used dchip_2010_01.exe). Rename this file to dchip.exe (drop the version info) and copy this .exe to CEL folder. Please contact the corresponding author John Storey (jstorey@princeton.edu) after being granted access to the full data set, and he will provide explicit instructions for incorporating the raw clinical and expression data into this analysis workflow bundle.Section 2: Running the *.R scripts in a quick and simple wayTo run the *.R scripts in the code folder, double click on runAnalysis.Rdata in the working directory. The following text should appear in the R console:                To run the analysis we have performed, we                will ask you a series of questions to set up                 the analysis. See Information.rtf in the                 documentation subfolder for more information.                Press Escape at any time to abort.                  Please choose one of the following options:1 -- To continue with the analysis2 -- To exit Enter number: If the above text does not appear, in order to get the above text, you will need to type the following string in the R console after the symbol ">". start.analysis()Once the above string is typed into the R console, hit the enter button. The above text should now appear on the R console.To reduce the time required to run the entire analysis, you may wish to bypass the normalization step. To do so, make sure you enter the number 2 when asked the following question:  If you are on a windows machine, do you want to perform dChip normalization on your PC? If you are not on a windows machine, please enter the number 2. 1 -- Yes  2 -- No, I do not want to perform normalization on my PC, or I am not on a windows machine Enter number: To get figure files with fine details as the paper, you will have to plot some of the figures as *.png files. To do so, make sure your computer can save figures as *.png files and then enter the number 1 when asked the following question:  If you are on a computer machine that allows you to save figures as *.png files, do you want to plot the gene sets figures in png? If you are unsure, please enter the number 2. 1 -- Yes  2 -- No, I do not want to plot these figures in png, or I am unsure if my computer allows me to save figures as *.png files Enter number:After answering a series of three questions, R will run the analysis based on the answers provided. It will take a couple of hours to a day or two depending on the computer make-up and the answers provided.Section 3: Workflow for normalizing microarrays1. There are 168 patients and altogether 797 microarrays were collected from hour 0 to 800.2. For the analysis, we restricted our attention to microarrays collected from hour 0 to 250 and with RNA quality >1 (604 microarrays), and these microarrays are normalized using dchip. 3. WPEC (i.e. the slope) is computed for patients with >2 microarrays. Altogether 129 patients' WPEC were computed. Three patients were removed because: (a) 1 patient died from head injuries, (b) 2 patients had data quality issues (see Supplementary Figure 21).4. Altogether there were 126 patients used in associating WPEC with ocMOF.Section 4: Plotting dominant trajectories of gene sets and heatmap of expression from hour 0 to 8001. We replace "HOURS_SINCE_INJURY" (taken from TRDB_TRAUMA-PT_DEMO_MICRO_SVRTY_RPT_20090824_105202.xls) of patientID: 4501723 with microarray id t20874672 from 16.3 to 14.5, so as to be consistent with the local database.2. For patientID: 16832704, the microarray id for "HOURS_SINCE_INJURY" at 332.1 is t18707233 for the local database and t23586509 for TRDB. The two *.CEL files are identical. We use the microarray id t18707233 so as to be consistent with the local database. Section 5: R libraries used in this analysisgdata, gplots, impute, nnet, pscl, qvalue
