# Supplementary material for: Dissecting Inflammatory Complications in Critically Injured Patients by Within-Patient Gene Expression Changes: A Longitudinal Clinical Genomics Study
Source: PLoS Med. 2011 Sep 13;8(9):e1001093. doi: 10.1371/journal.pmed.1001093 (PMC3172280; doi:10.1371/journal.pmed.1001093)
Supplement: Dataset S1 — Annotated scripts that reproduce the results in the paper. The scripts run the entire analysis in R statistical software (cran.r-project.org). See Text S2 for the details and http://genomine.org/trauma/ for instructions on obtaining the full dataset. (ZIP) [file pmed.1001093.s001.zip › README.rtf]

This folder, which is the working directory, contains files and data used in the paper.The analysis and figures in the paper were mainly generated in R. For those who are new to R, a quick and simple way to run the scripts is provided, see Information.rtf in the documentation folder for the details. For those who are familiar with running R on the command line, they may wish to run the main.R file in the code folder.An overview of the subfolders in the working directory:1. documentationContains information of the structure of this directory and how to run the codes provided in a quick and simple way. Read Information.rtf to get an overall perspective of the working directory and how to run the codes. The README.rtf in each of the subfolder contains detailed information of its contents. 2. codeContains the *.R files for running the analysis and generating the figures in the paper. The main.R file runs the entire analysis and generate the figures in the paper by calling *.R scripts in its subfolders.3. dataContains the data used in the paper: microarray data and clinical data with their annotation files. All of the data are freely available at www.gluegrant.org for registered researchers. "Members who seek access to the human research data will have been granted from their home institution, institutional review board (IRB) approval to receive human research data in a manner consistent with the protection of confidentiality of the subjects" (www.gluegrant.org/glueadmin/register_consortium.jsp). Note that in order to reproduce normalization of the raw data (CEL files), the user will need to obtain the dChip normalization software from https://sites.google.com/site/dchipsoft/ (we used dchip_2010_01.exe). Rename this file to dchip.exe (drop the version info) and copy this .exe to CEL folder.  Please contact the corresponding author John Storey (jstorey@princeton.edu) after being granted access to the full data set, and he will provide explicit instructions for incorporating the raw clinical and expression data into this analysis workflow bundle.An overview of the files in the working directory:1. README.rtfContains detailed information of the working directory.2. runAnalysis.RdataA *.Rdata file that makes running the *.R scripts simple and fast. See Information.rtf in the documentation folder for the details.If no text appears, you will need to type the following string in the R console after the symbol ">". start.analysis()Once the above string is typed into the R console, hit the enter button. The scripts should start running now. 
