# Supplementary material for: Dissecting Inflammatory Complications in Critically Injured Patients by Within-Patient Gene Expression Changes: A Longitudinal Clinical Genomics Study
Source: PLoS Med. 2011 Sep 13;8(9):e1001093. doi: 10.1371/journal.pmed.1001093 (PMC3172280; doi:10.1371/journal.pmed.1001093)
Supplement: Figure S1 — Microarray collection time points by patient. X-axis is the time from injury and Y-axis patient IDs. Each circle represents a microarray collected. Intended sampling was on days 0, 1, 4, 7, 14, 21, and 28 since injury, but depending on the total days from injury to discharge/death, the number of microarrays per patient ranged between 2 to 7. (PDF) [file pmed.1001093.s002.pdf]

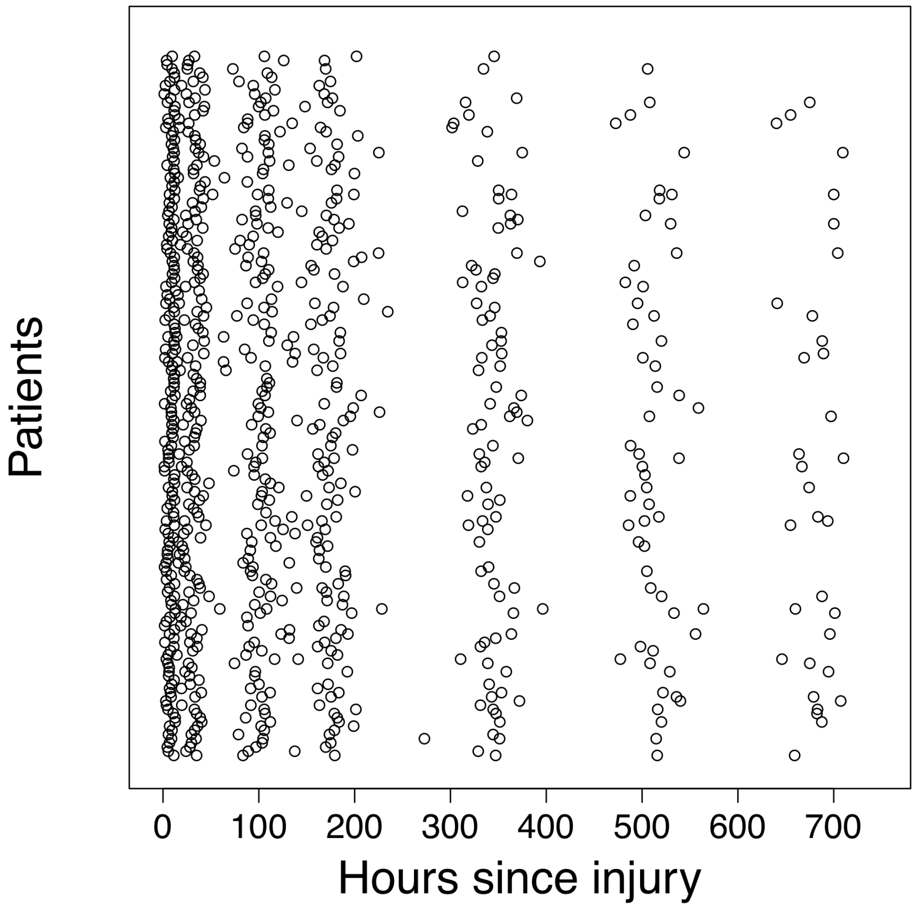

Supplementary Figure 1. Microarray collection timepoints by patient. X-axis is the time from injury and Y-axis patient IDs. Each circle represents a microarray collected. Intended sampling was on day 0, 1, 4, 7, 14, 21 and 28 since injury, but depending on the total days from injury to discharge/death, the number of microarrays per patient ranged between 2 to 7.
