# Supplementary material for: Dissecting Inflammatory Complications in Critically Injured Patients by Within-Patient Gene Expression Changes: A Longitudinal Clinical Genomics Study
Source: PLoS Med. 2011 Sep 13;8(9):e1001093. doi: 10.1371/journal.pmed.1001093 (PMC3172280; doi:10.1371/journal.pmed.1001093)
Supplement: Figure S2 — Heatmap of patient–patient correlations. Using the WPEC matrix we computed patient–patient correlations for 129 patients. The heatmap of dichotomized correlations (black = negative; gray = positive) identified two patients as outliers with completely opposite correlations from the rest. We removed these two patients due to potential array quality issues. (PDF) [file pmed.1001093.s003.pdf]

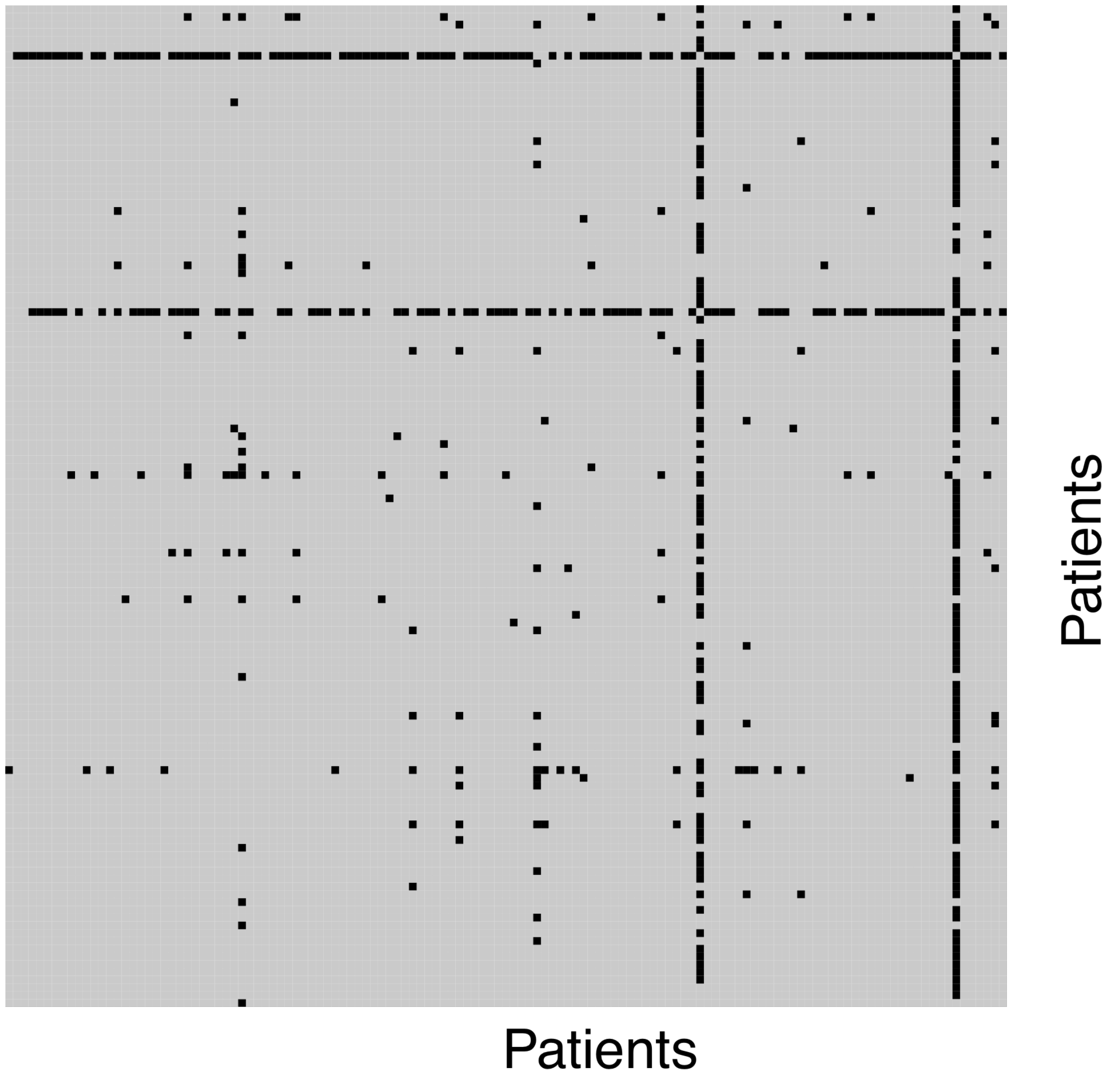

Supplementary Figure 2. Heatmap of patient-patient correlations. Using the WPEC matrix we computed patient-patient correlations for 129 patients. The heatmap of dichotomized correlations (black=negative and gray=positive) identified two patients as outliers with completely opposite correlations from the rest. We removed these two patients due to potential array quality issues.
