# Supplementary material for: Dissecting Inflammatory Complications in Critically Injured Patients by Within-Patient Gene Expression Changes: A Longitudinal Clinical Genomics Study
Source: PLoS Med. 2011 Sep 13;8(9):e1001093. doi: 10.1371/journal.pmed.1001093 (PMC3172280; doi:10.1371/journal.pmed.1001093)
Supplement: Figure S3 — Heatmap of the modified Marshall scores on day 0, 2, 3, …, 20 and the dendrogram of the hierarchical clustering. Hierarchical clustering was performed on the modified Marshall score trajectories, where missing scores were imputed using k-nearest neighbor. The left plot is the dendrogram of the hierarchical clustering from which we obtained five subgroups: ocMOF i to v. Patients from ocMOF i to iii tend to have low modified Marshall scores, with patients with ocMOF i recovering to 0 first, followed by ocMOF ii and iii, while patients from ocMOF iv and v tend to have high modified Marshall scores throughout the first 20 d. (PDF) [file pmed.1001093.s004.pdf]

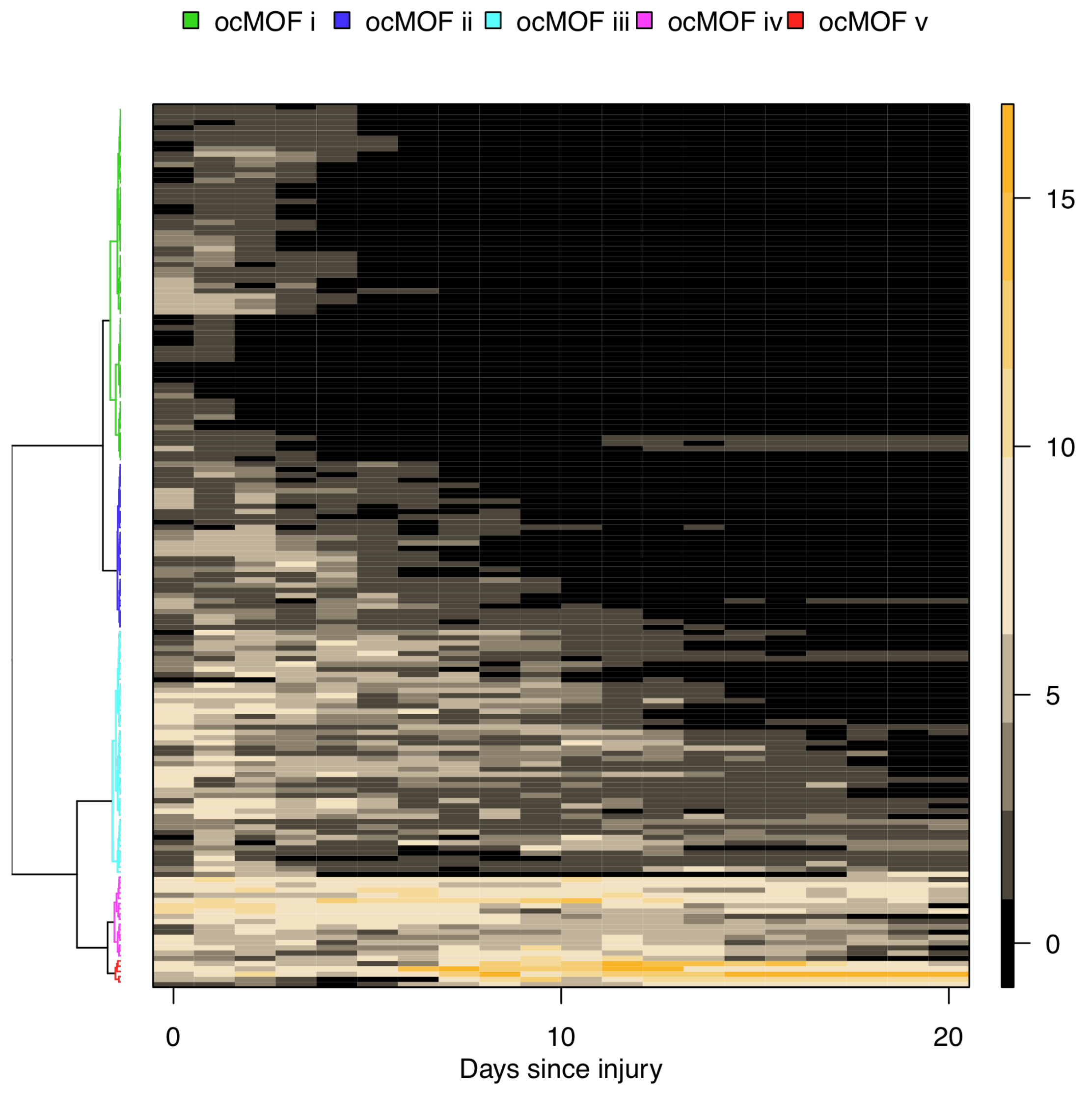

Supplementary Figure 3. Heatmap of the modified Marshall scores on day 0, 2, 3, …, 20 and the dendrogram of the hierarchical clustering. Hierarchical clustering was performed on the modified Marshall score trajectories, where missing scores were imputed using k-nearest neighbor. The left plot is the dendrogram of the hierarchical clustering from which we obtained five subgroups: *ocMOF i* to *v*. Patients from *ocMOF i* to *iii* tend to have low modified Marshall scores, with patients with *ocMOF i* recovering to 0 first, followed by *ocMOF ii* and *iii*, while patients from *ocMOF iv* and *v* tend to have high modified Marshall scores throughout the first 20 days.
