# Supplementary material for: Dissecting Inflammatory Complications in Critically Injured Patients by Within-Patient Gene Expression Changes: A Longitudinal Clinical Genomics Study
Source: PLoS Med. 2011 Sep 13;8(9):e1001093. doi: 10.1371/journal.pmed.1001093 (PMC3172280; doi:10.1371/journal.pmed.1001093)
Supplement: Figure S4 — Marshall score trajectories and ocMOF. Thin dashed lines in gray correspond to patient-specific Marshall score trajectories, and thick solid lines to the mean trajectories of the ocMOF subgroup. Only the observed modified Marshall scores are used to make these plots, but the actual clustering was performed on imputed data. Note that four out of five patients with ocMOF v died on or before day 10 post-injury, and that the red dashed line is for the remaining patient who died on day 24 post-injury. Mean ocMOF trajectories, together with other relevant patient clinical information, allowed us to order the ocMOF clusters in terms of overall patient severity. In particular, ocMOF i = good outcome (fast and uncomplicated recovery) and ocMOF v = very bad outcome (death). (PDF) [file pmed.1001093.s005.pdf]

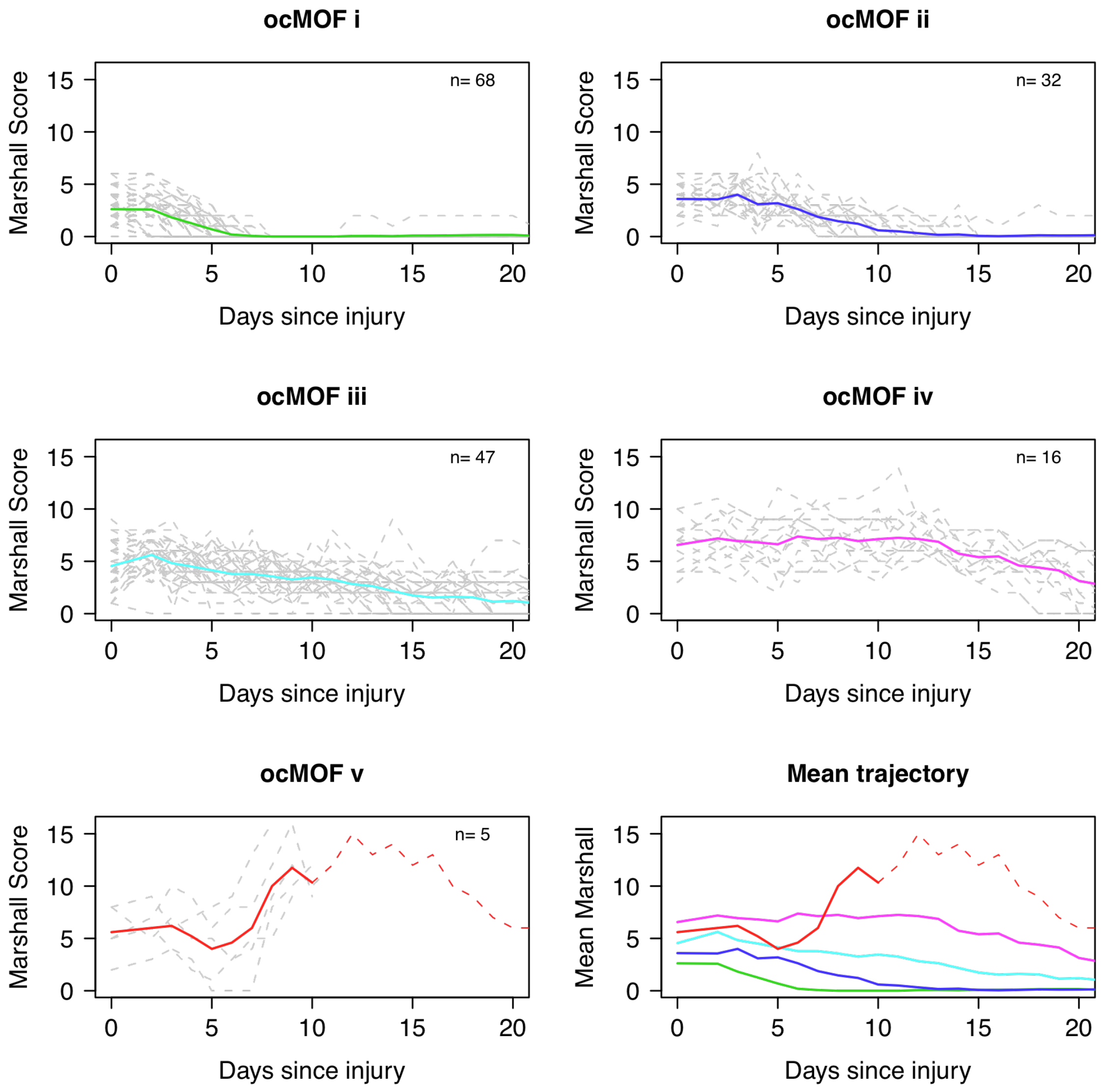


**Supplementary Figure 4. Marshall score trajectories and ocMOF.** Thin dashed lines in gray correspond to patient-specific Marshall score trajectories and thick solid lines to the mean trajectories of the ocMOF subgroup. Only the observed modified Marshall scores are used to make these plots, but the actual clustering was performed on imputed data. Note that 4 out of 5 patients with *ocMOF v* died on or before day 10 since injury, and that the red dashed line is for the remaining patient who died on day 24 since injury. Mean ocMOF trajectories together with other relevant patient clinical information allowed us to order the ocMOF clusters in terms of overall patient severity. In particular, *ocMOF i* = good outcome (fast and uncomplicated recovery) and *ocMOF v* = very bad outcome (death).
