# Supplementary material for: Dissecting Inflammatory Complications in Critically Injured Patients by Within-Patient Gene Expression Changes: A Longitudinal Clinical Genomics Study
Source: PLoS Med. 2011 Sep 13;8(9):e1001093. doi: 10.1371/journal.pmed.1001093 (PMC3172280; doi:10.1371/journal.pmed.1001093)
Supplement: Figure S5 — Probesets with different dynamics. (a–d) Expression trajectory of probesets in the time window 0–250 h (shown in a and c) and 12–250 hours (shown in b and d). Patient-specific trajectories are represented by gray lines, and population average trajectories and population average linear trajectories are represented by the black and red lines respectively. (a) and (b) correspond to the most non-significant probeset from the DWPEC analysis, where the differences between the average trajectory and average linear trajectory are minimal. (c) and (d) correspond to the most significant probeset from the DWPEC analysis, where the differences between the average trajectory and average linear trajectory are large in time window 0–250 h but are reduced in time window 12–250 h. (PDF) [file pmed.1001093.s006.pdf]

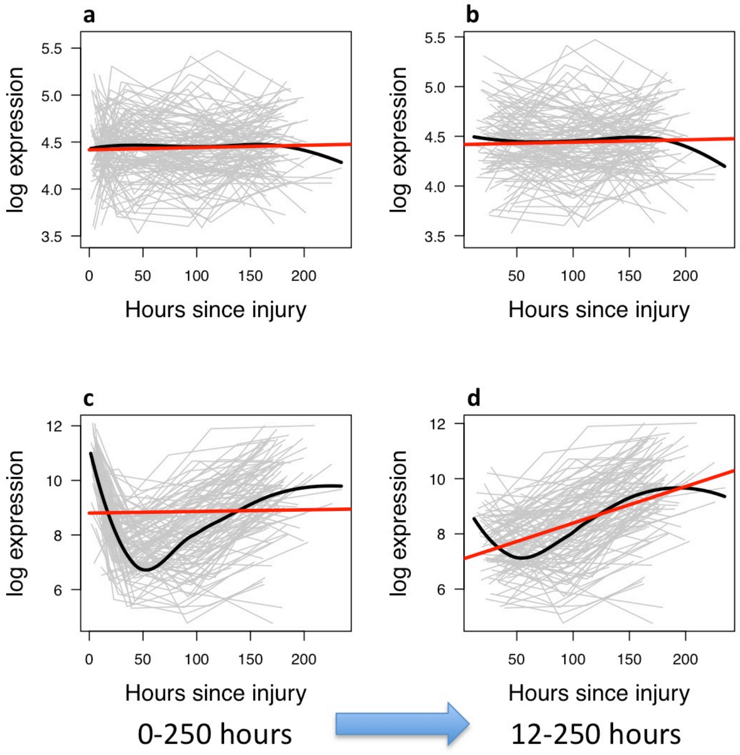

Supplementary Figure 5. Probesets with different dynamics. a-d, Expression trajectory of probesets in the time window 0 to 250 hours (shown in **a** and **c**) and 12 to 250 hours (shown in **b** and **d**). Patient-specific trajectories are represented by gray lines, and population average trajectories and population average linear trajectories are represented by the black and red lines respectively. Panels **a** and **b** correspond to the most non-significant probeset from the DWPEC analysis, where the differences between the average trajectory and average linear trajectory are minimal. Panels **c** and **d** correspond to the most significant probeset from the DWPEC analysis, where the differences between the average trajectory and average linear trajectory are large in time window 0 to 250 hours but are reduced in time window 12 to 250 hours.
