# Supplementary material for: Dissecting Inflammatory Complications in Critically Injured Patients by Within-Patient Gene Expression Changes: A Longitudinal Clinical Genomics Study
Source: PLoS Med. 2011 Sep 13;8(9):e1001093. doi: 10.1371/journal.pmed.1001093 (PMC3172280; doi:10.1371/journal.pmed.1001093)
Supplement: Figure S6 — Reasoning to exclude hours <12. Boxplots of mean-square difference (MSD) between the population average trajectory and average linear trajectory for the 5,000 most non-significant (ns) and significant (sig) probesets from the DWPEC analysis. We investigated the MSD for four different time windows: hour 0–250, hour 4–250, hour 8–250 and hour 12–250. The MSDs for the non-significant probesets are very similar across all four time windows, but the MSDs for the significant probesets are generally high for hour 0–250 and tend to decrease as we progressively exclude the early hours. (PDF) [file pmed.1001093.s007.pdf]

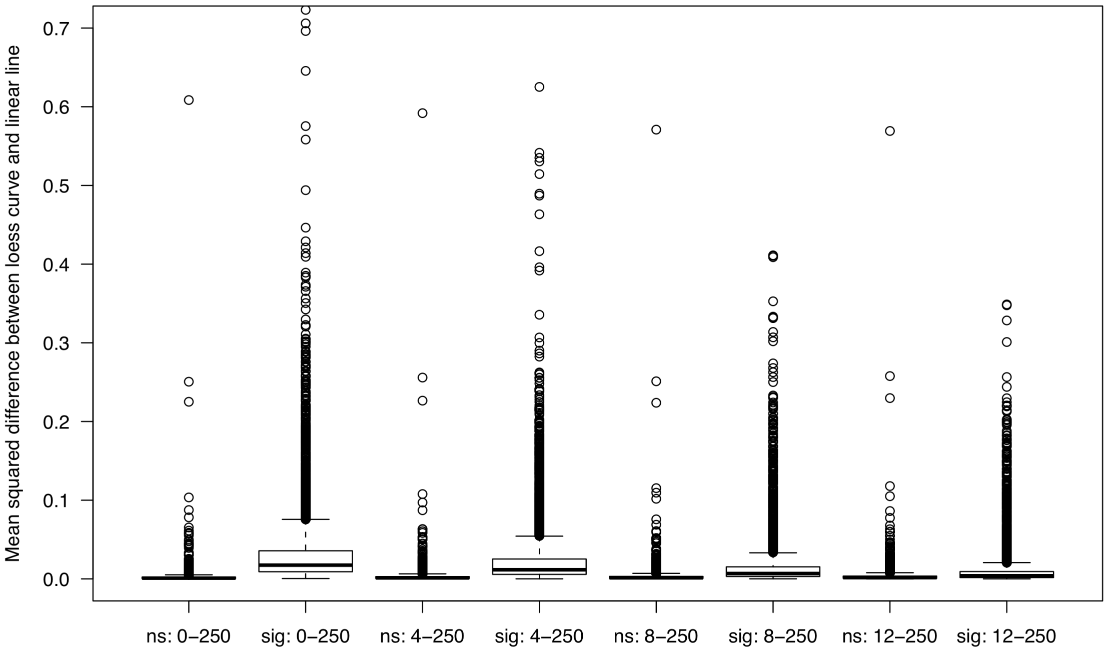

Supplementary Figure 6. Reasoning to exclude hours <12. Boxplots of mean-square difference (MSD) between the population average trajectory and average linear trajectory for the 5000 most non-significant (ns) and significant (sig) probesets from the DWPEC analysis. We investigated the MSD for four different time windows: hour 0-250, hour 4-250, hour 8-250 and hour 12-250. The MSDs for the non-significant probesets are very similar across all four time windows, but the MSDs for the significant probesets are generally high for hour 0 to 250 and tend to decrease as we progressively exclude the early hours.
