# Supplementary material for: Dissecting Inflammatory Complications in Critically Injured Patients by Within-Patient Gene Expression Changes: A Longitudinal Clinical Genomics Study
Source: PLoS Med. 2011 Sep 13;8(9):e1001093. doi: 10.1371/journal.pmed.1001093 (PMC3172280; doi:10.1371/journal.pmed.1001093)
Supplement: Figure S7 — The scree plots to determine the number of principal components. (a) corresponds to the WPEC matrix and (b) to the mean expression matrix. (PDF) [file pmed.1001093.s008.pdf]

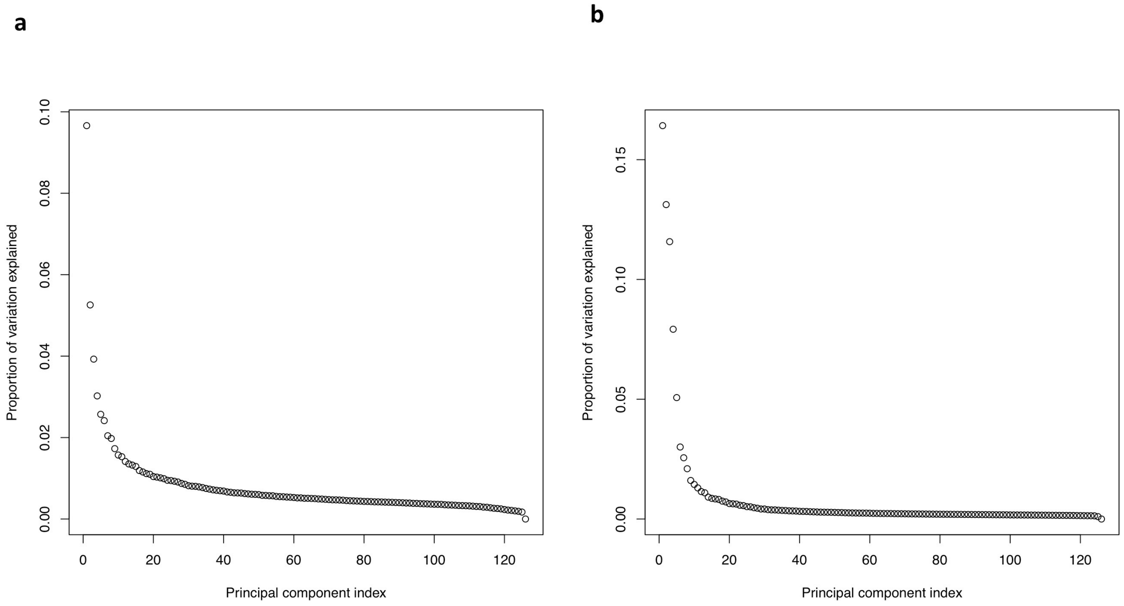

Supplementary Figure 7. The scree plots to determine the number of principal components. Panel a corresponds to the WPEC matrix and panel b to the mean expression matrix.
