# Supplementary material for: Dissecting Inflammatory Complications in Critically Injured Patients by Within-Patient Gene Expression Changes: A Longitudinal Clinical Genomics Study
Source: PLoS Med. 2011 Sep 13;8(9):e1001093. doi: 10.1371/journal.pmed.1001093 (PMC3172280; doi:10.1371/journal.pmed.1001093)
Supplement: Figure S8 — The dendrogram for grouping the 54 functional related gene sets into five modules according to the similarity of their dominant trajectories across the ocMOF subgroups. (PDF) [file pmed.1001093.s009.pdf]

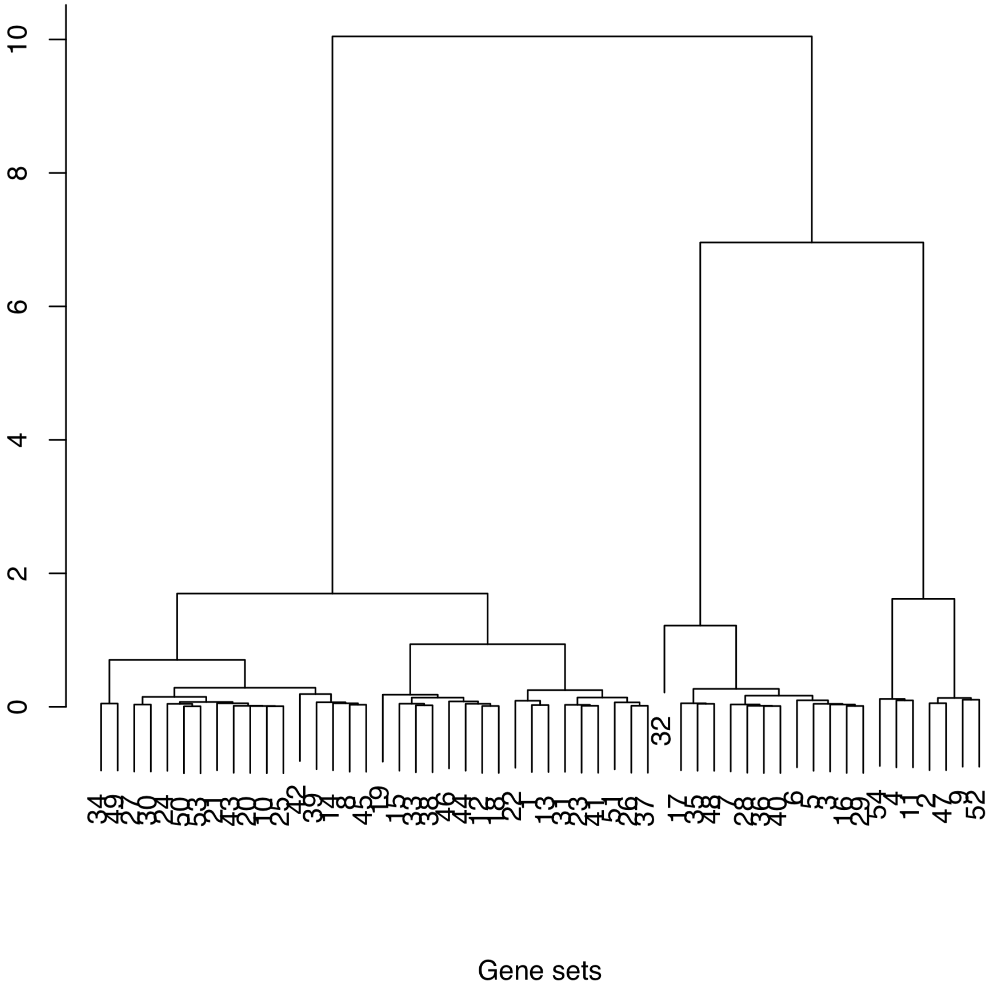

Supplementary Figure 8. The dendrogram for grouping the 54 functional related gene sets into 5 modules according to the similarity of their dominant trajectories across the ocMOF subgroups.
