# Supplementary material for: Dissecting Inflammatory Complications in Critically Injured Patients by Within-Patient Gene Expression Changes: A Longitudinal Clinical Genomics Study
Source: PLoS Med. 2011 Sep 13;8(9):e1001093. doi: 10.1371/journal.pmed.1001093 (PMC3172280; doi:10.1371/journal.pmed.1001093)
Supplement: Figure S9 — The dominant trajectories for Module A. For each ocMOF subgroup, where (a)–(e) correspond to ocMOF i to v, the dominant trajectories of the module (thick colored lines) are obtained by averaging all dominant trajectories of gene sets belonging to the module (gray lines). (f) plots all five ocMOF subgroup dominant trajectories for Module A in one plot by aligning them to a common initial reference. (PDF) [file pmed.1001093.s010.pdf]

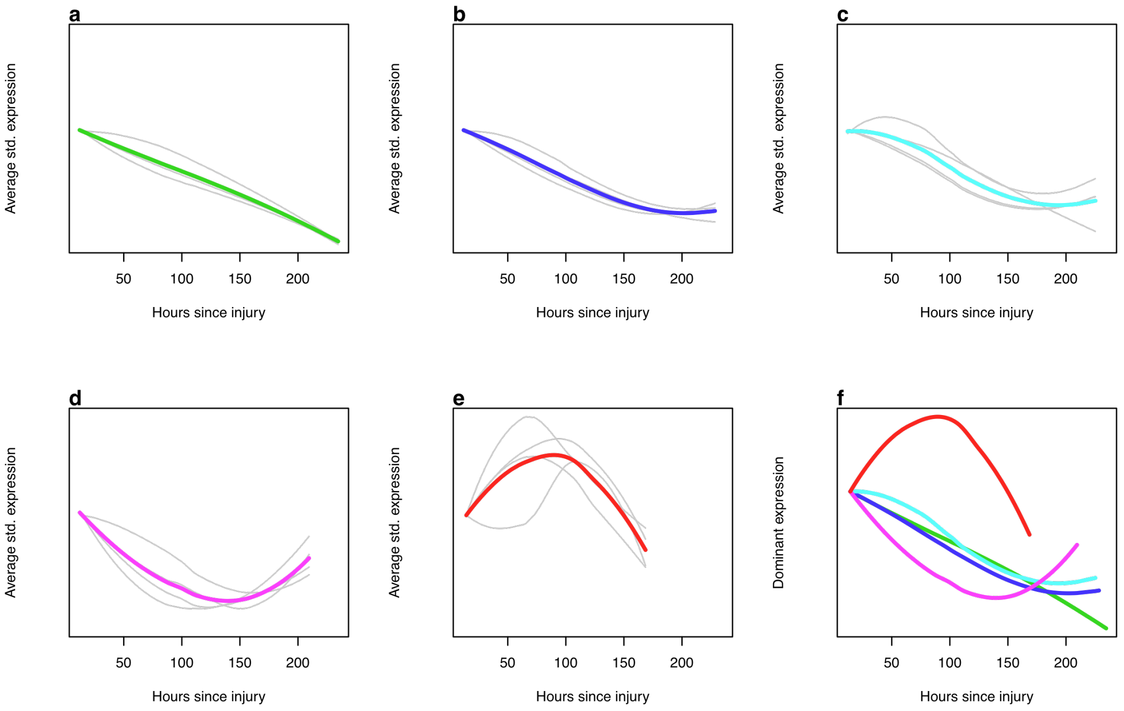

Supplementary Figure 9. The dominant trajectories for Module A. For each ocMOF subgroup, where panel **a** to **e** correspond to *ocMOF i* to *v*, the dominant trajectories of the module (thick colored lines) are obtained by averaging all dominant trajectories of gene sets belonging to the module (gray lines). Panel **f** plots all five ocMOF subgroup dominant trajectories for module A in a one plot by aligning them to a common initial reference.
