# Supplementary material for: Dissecting Inflammatory Complications in Critically Injured Patients by Within-Patient Gene Expression Changes: A Longitudinal Clinical Genomics Study
Source: PLoS Med. 2011 Sep 13;8(9):e1001093. doi: 10.1371/journal.pmed.1001093 (PMC3172280; doi:10.1371/journal.pmed.1001093)
Supplement: Figure S13 — The dominant trajectories for Module E. See the caption for Figure S9 for details. (PDF) [file pmed.1001093.s014.pdf]

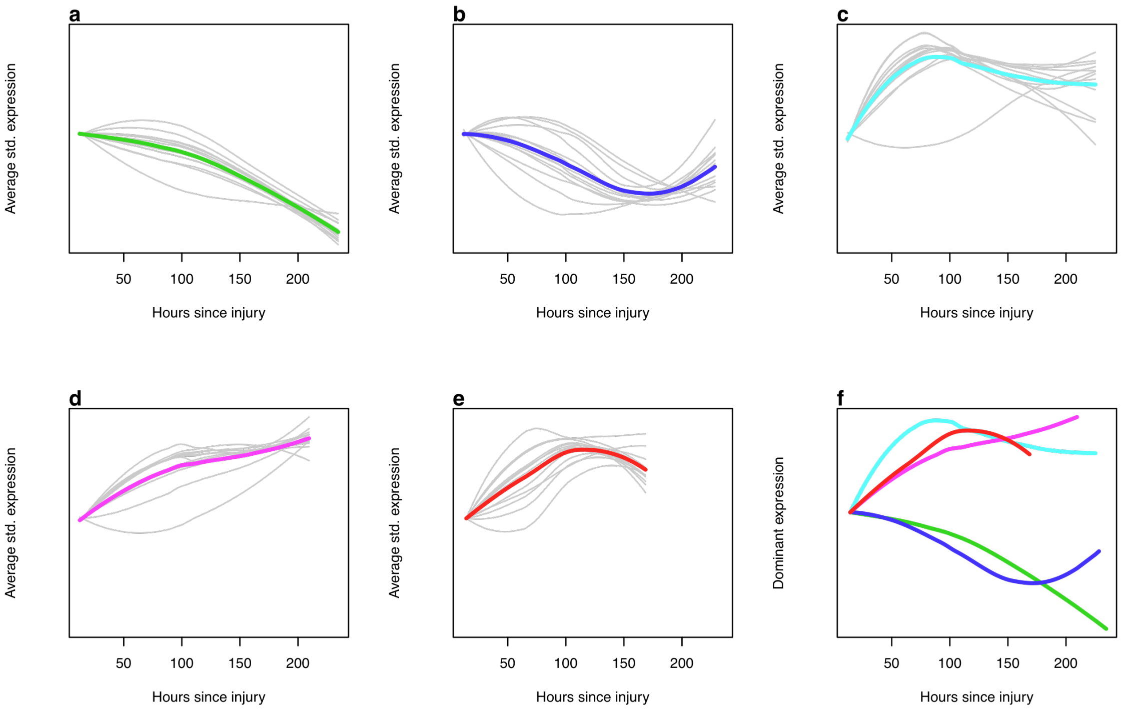

Supplementary Figure 13. The dominant trajectories for Module E. See the caption for Supp. Fig. 9 for details.
