# Supplementary material for: Dissecting Inflammatory Complications in Critically Injured Patients by Within-Patient Gene Expression Changes: A Longitudinal Clinical Genomics Study
Source: PLoS Med. 2011 Sep 13;8(9):e1001093. doi: 10.1371/journal.pmed.1001093 (PMC3172280; doi:10.1371/journal.pmed.1001093)
Supplement: Figure S14 — The heatmap of ranked gene expressions for all 168 patients over 28 d for the 500 most significant probesets from our analysis. For each probeset, we ranked the expression values across all 168 patients over 28 d, i.e. 797 microarrays (green = low rank, black = average rank, red = high rank). The columns are microarrays ordered by days, and within each day by ocMOF values. The intended sampling was on days 0, 1, 4, 7, 14, 21, and 28 since injury. (PDF) [file pmed.1001093.s015.pdf]

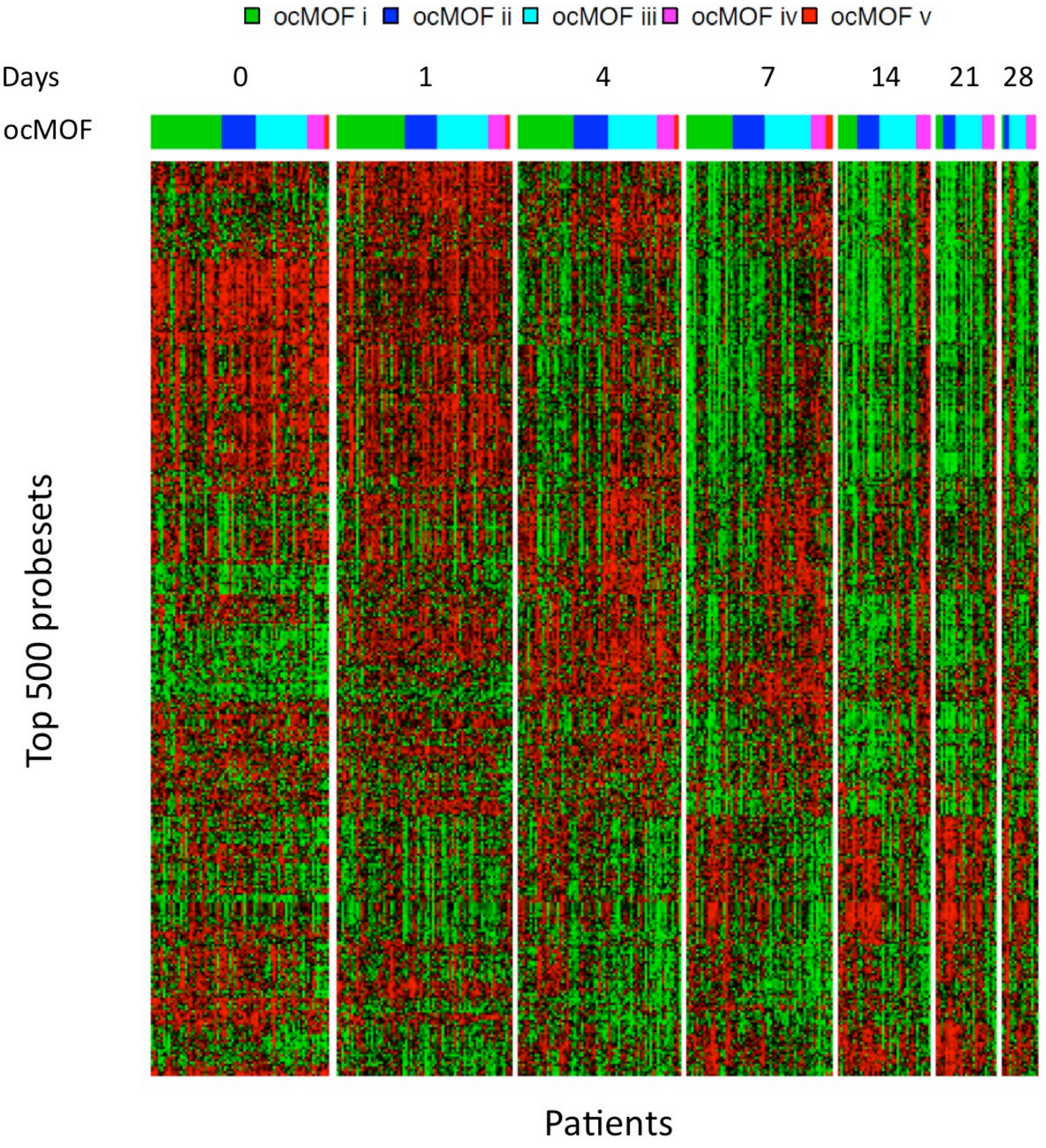

Supplementary Figure 14. The heatmap of ranked gene expressions for all 168 patients over 28 days for the 500 most significant probesets from our analysis. For each probeset, we ranked the expression values across all 168 patients over 28 days, i.e. 797 microarrays (green=low rank, black=average rank, red=high rank). The columns are microarrays ordered by days, and within each day by ocMOF values. The intended sampling was on days 0, 1, 4, 7, 14, 21 and 28 since injury.
