# Supplementary material for: Dissecting Inflammatory Complications in Critically Injured Patients by Within-Patient Gene Expression Changes: A Longitudinal Clinical Genomics Study
Source: PLoS Med. 2011 Sep 13;8(9):e1001093. doi: 10.1371/journal.pmed.1001093 (PMC3172280; doi:10.1371/journal.pmed.1001093)
Supplement: Figure S15 — The antigen presentation pathway. The MHC-II genes have negative spearman correlation coefficients between WPEC and ocMOF (colored blue). (PDF) [file pmed.1001093.s016.pdf]

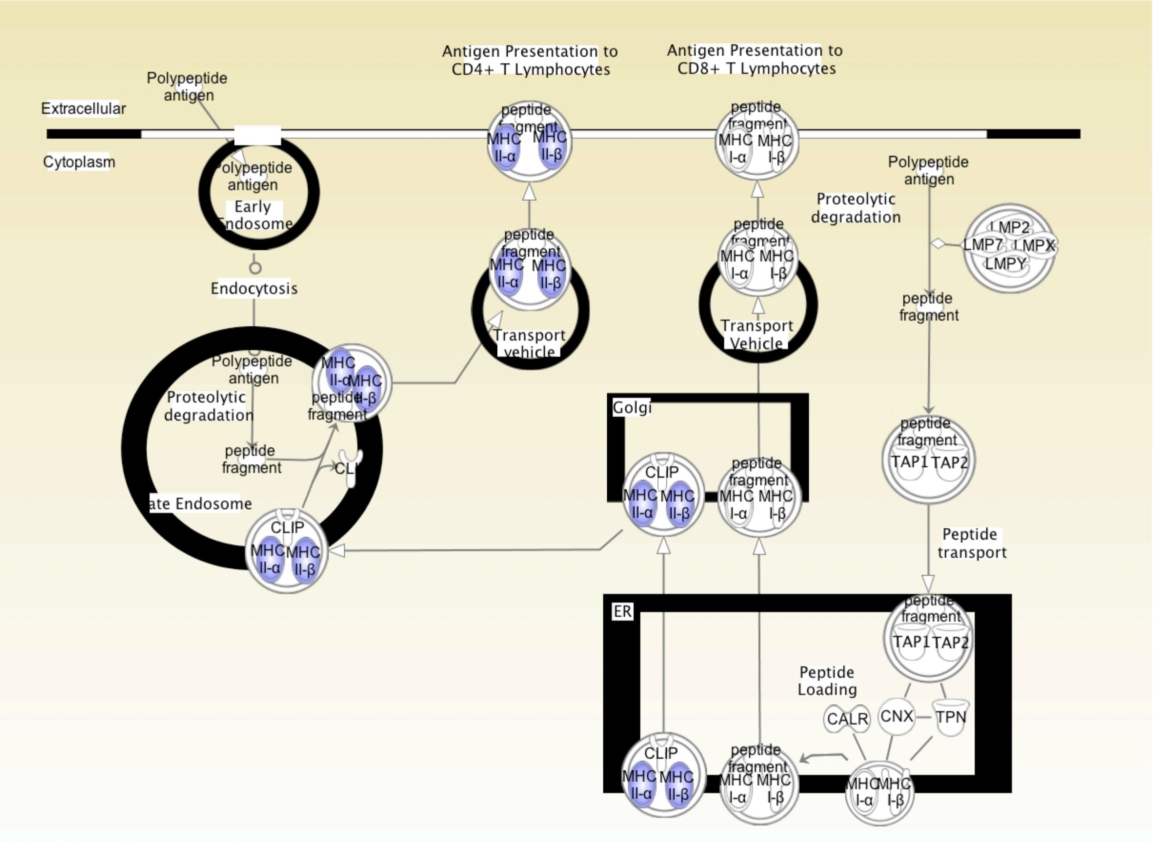

© 2000-2010 Ingenuity Systems, Inc. All rights reserved.

Supplementary Figure 15. The antigen presentation pathway. The MHC-II genes have negative spearman correlation coefficients between WPEC and ocMOF (colored blue).
