# Supplementary material for: Dissecting Inflammatory Complications in Critically Injured Patients by Within-Patient Gene Expression Changes: A Longitudinal Clinical Genomics Study
Source: PLoS Med. 2011 Sep 13;8(9):e1001093. doi: 10.1371/journal.pmed.1001093 (PMC3172280; doi:10.1371/journal.pmed.1001093)
Supplement: Figure S16 — ocMOF and gene expression dynamics of MHC-II. (a) Boxplots of WPEC versus ocMOF indicate a negative dosage effect as we go from ocMOF i to v (p-value of the Spearman's test <10−15). (b) For each ocMOF group, the dominant trajectory (thick colored line) was obtained by averaging all the standardized MHC-II probeset trajectories (gray lines) of patients within the ocMOF subgroup. Generally, the dominant trajectory increases with time for ocMOF i and ii, initially decreases and then increases for ocMOF iii, and decreases for ocMOF iv and v. Both WPEC and the dominant trajectories exhibit similar trend. (c) The dominant trajectories within the first 100 h suggest that early expression changes (gray region) correlate with patient outcome. (d) The number of up-regulated MHC-II probesets (computed using two time points near hour 40–80) separates patients with ocMOF i, ii, and iii from patients with ocMOF iv and v (p-value of the Kruskal-Wallis test is 0.00004). (PDF) [file pmed.1001093.s017.pdf]

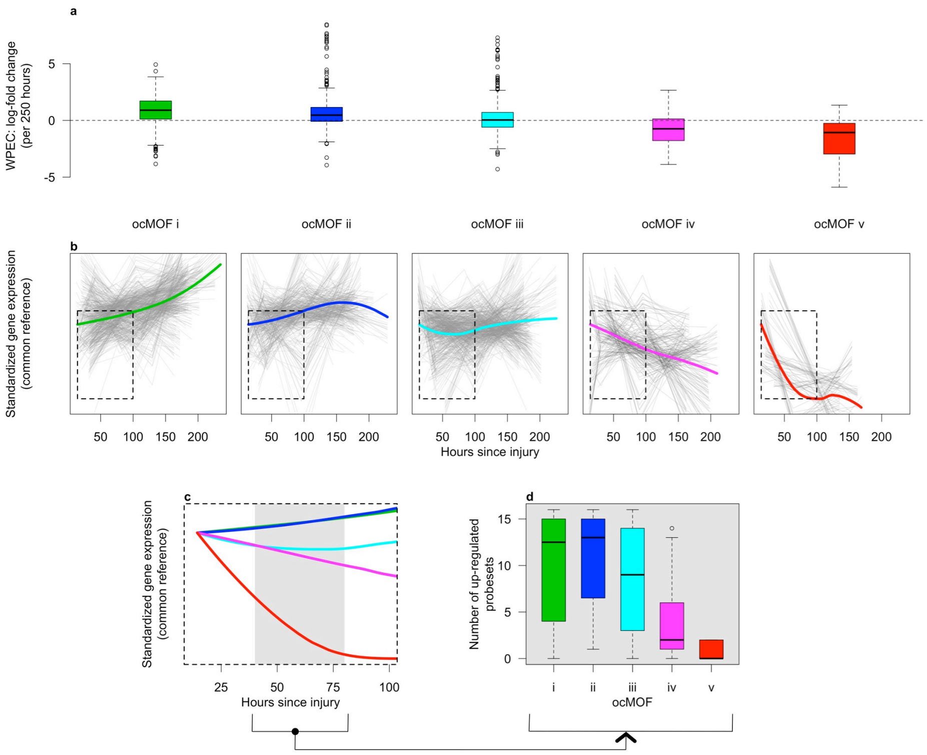

Supplementary Figure 16. ocMOF and gene expression dynamics of MHC-II. **a**, Boxplots of WPEC versus ocMOF indicate a negative dosage effect as we go from *ocMOF i* to *v* (p-value of the Spearman's test < 10^{-15}). **b**, For each ocMOF group, the dominant trajectory (thick colored line) was obtained by averaging all the standardized MHC-II probeset trajectories (gray lines) of patients within the ocMOF subgroup. Generally, the dominant trajectory increases with time for *ocMOF i* and *ii*, initially decreases and then increases for *ocMOF iii*, and decreases for *ocMOF iv* and *v*. Both WPEC and the dominant trajectories exhibit similar trend. **c**, The dominant trajectories within the first 100 hours suggest that early expression changes (gray region) correlate with patient outcome. **d**, The number of up-regulated MHC-II probesets (computed using two time points near hour 40 to 80) separates patients with *ocMOF i*, *ii*, and *iii* from patients with *ocMOF iv* and *v* (p-value of the Kruskal-Wallis test is 0.00004).
