# Supplementary material for: Dissecting Inflammatory Complications in Critically Injured Patients by Within-Patient Gene Expression Changes: A Longitudinal Clinical Genomics Study
Source: PLoS Med. 2011 Sep 13;8(9):e1001093. doi: 10.1371/journal.pmed.1001093 (PMC3172280; doi:10.1371/journal.pmed.1001093)
Supplement: Figure S17 — The p38 MAPK signaling pathway. Among the top 500 probesets, 15 are in this canonical pathway (representing 11 genes). Those genes in blue and red have negative and positive Spearman correlation coefficients between WPEC and ocMOF, respectively. TRADD, MEF2, and Max were removed from further analysis because their correlations were inconsistent with those identified by IPA. (PDF) [file pmed.1001093.s018.pdf]

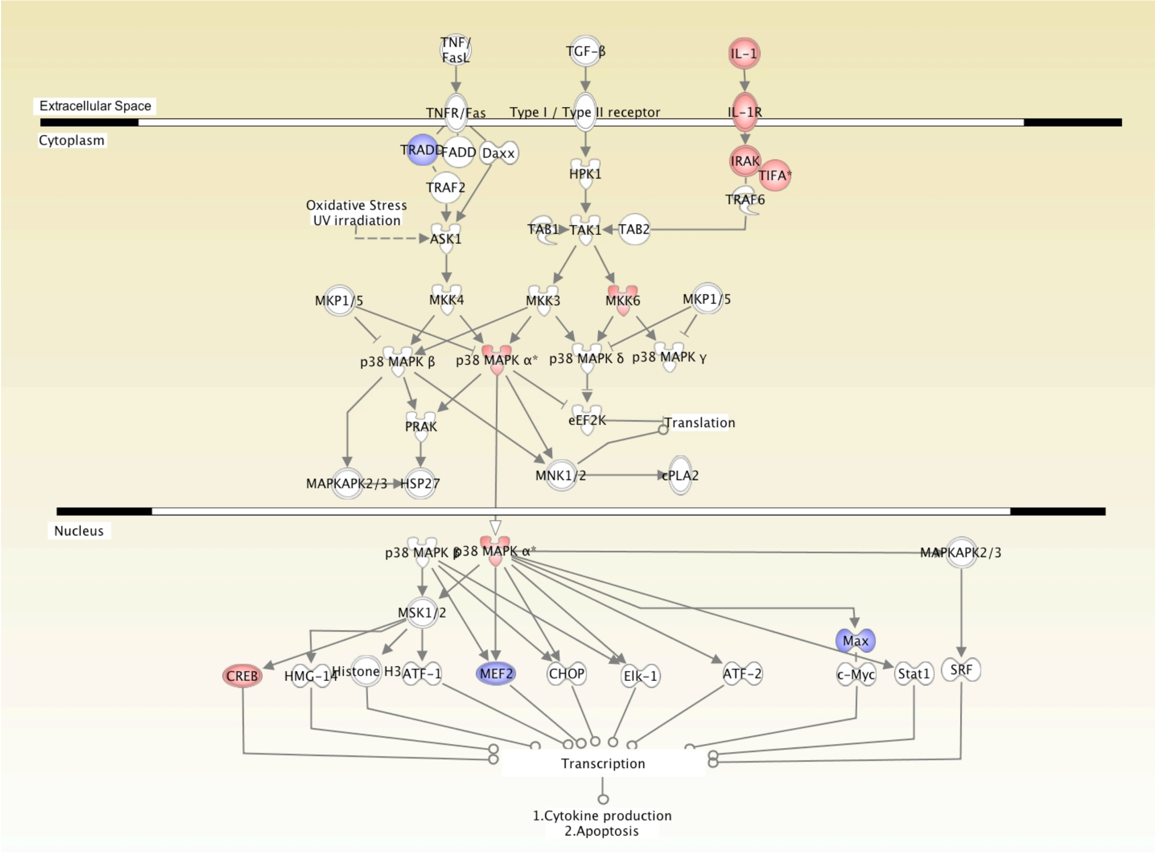

© 2000-2010 Ingenuity Systems, Inc. All rights reserved.

Supplementary Figure 17. The p38 MAPK signaling pathway. Among the top 500 probesets, 15 are in this canonical pathway (representing 11 genes). Those genes in blue and red have negative and positive spearman correlation coefficients between WPEC and ocMOF respectively. TRADD, MEF2 and Max were removed from further analysis because their correlations were inconsistent with those identified by IPA.
