# Supplementary material for: Dissecting Inflammatory Complications in Critically Injured Patients by Within-Patient Gene Expression Changes: A Longitudinal Clinical Genomics Study
Source: PLoS Med. 2011 Sep 13;8(9):e1001093. doi: 10.1371/journal.pmed.1001093 (PMC3172280; doi:10.1371/journal.pmed.1001093)
Supplement: Figure S18 — Gene expression profiles of the 12 probesets involved in the p38 MAPK signaling pathway. (a) Boxplots of WPEC versus ocMOF indicate a positive dosage effect as we go from ocMOF i to v (p-value of the Spearman's test <10−15). (b) For each ocMOF group, the dominant trajectory (thick colored line) was obtained by averaging all the standardized p38 MAPK probeset trajectories (gray dotted lines) of patients within the ocMOF subgroup. Generally, the early dominant trajectory decreases with time for ocMOF i and ii, initially increases and then decreases for ocMOF iii, and increases for ocMOF iv and v. Both WPEC and the dominant trajectories exhibit similar trends. (c) The dominant trajectories within the first 100 h suggest that early expression changes (gray region) correlate with patient outcome. (d) The number of up-regulated p38 MAPK probesets (computed using two time points near hour 40–80) separates patients with ocMOF i, ii, and iii from patients with ocMOF iv and v (p-value of the Kruskal-Wallis test is 0.00668). (PDF) [file pmed.1001093.s019.pdf]

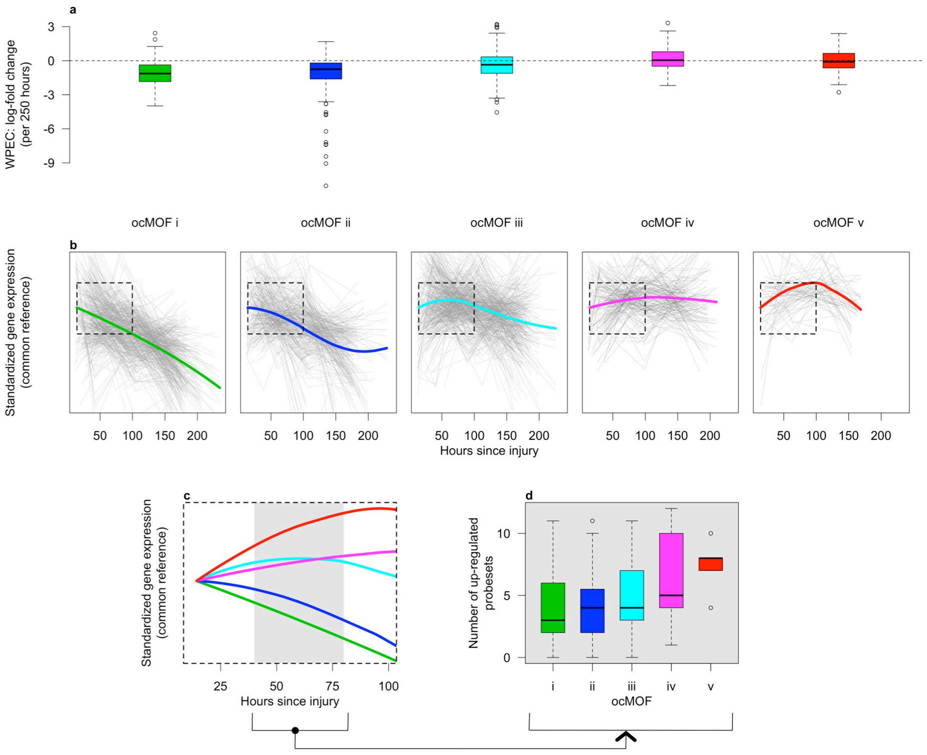

Supplementary Figure 18. Gene expression profiles of the 12 probesets involved in the p38 MAPK signaling pathway. a, Boxplots of WPEC versus ocMOF indicate a positive dosage effect as we go from *ocMOF i* to *v* (p-value of the Spearman's test $<10^{-15}$). **b**, For each ocMOF group, the dominant trajectory (thick colored line) was obtained by averaging all the standardized p38MAPK probeset trajectories (gray dotted lines) of patients within the ocMOF subgroup. Generally, the early dominant trajectory decreases with time for *ocMOF i* and *ii,* initially increases and then decreases for *ocMOF iii*, and increases for *ocMOF iv* and *v*. Both WPEC and the dominant trajectories exhibit similar trend. **c**, The dominant trajectories within the first 100 hours suggest that early expression changes (gray region) correlate with patient outcome. **d**, The number of up-regulated p38MAPK probesets (computed using two time points near hour 40 to 80) separates patients with *ocMOF i, ii, and iii* from patients with *ocMOF iv* and *v* (p-value of the Kruskal-Wallis test is 0.00668).
