# Supplementary material for: Dissecting Inflammatory Complications in Critically Injured Patients by Within-Patient Gene Expression Changes: A Longitudinal Clinical Genomics Study
Source: PLoS Med. 2011 Sep 13;8(9):e1001093. doi: 10.1371/journal.pmed.1001093 (PMC3172280; doi:10.1371/journal.pmed.1001093)
Supplement: Figure S19 — The Toll-like receptor (TLR) pathway. Among the top 500 probesets, 12 are in this canonical pathway (representing nine genes). Those genes in blue and red have negative and positive Spearman correlation coefficients between WPEC and ocMOF, respectively. JNK1 was removed from further analysis because its correlation was inconsistent with that identified by IPA. (PDF) [file pmed.1001093.s020.pdf]

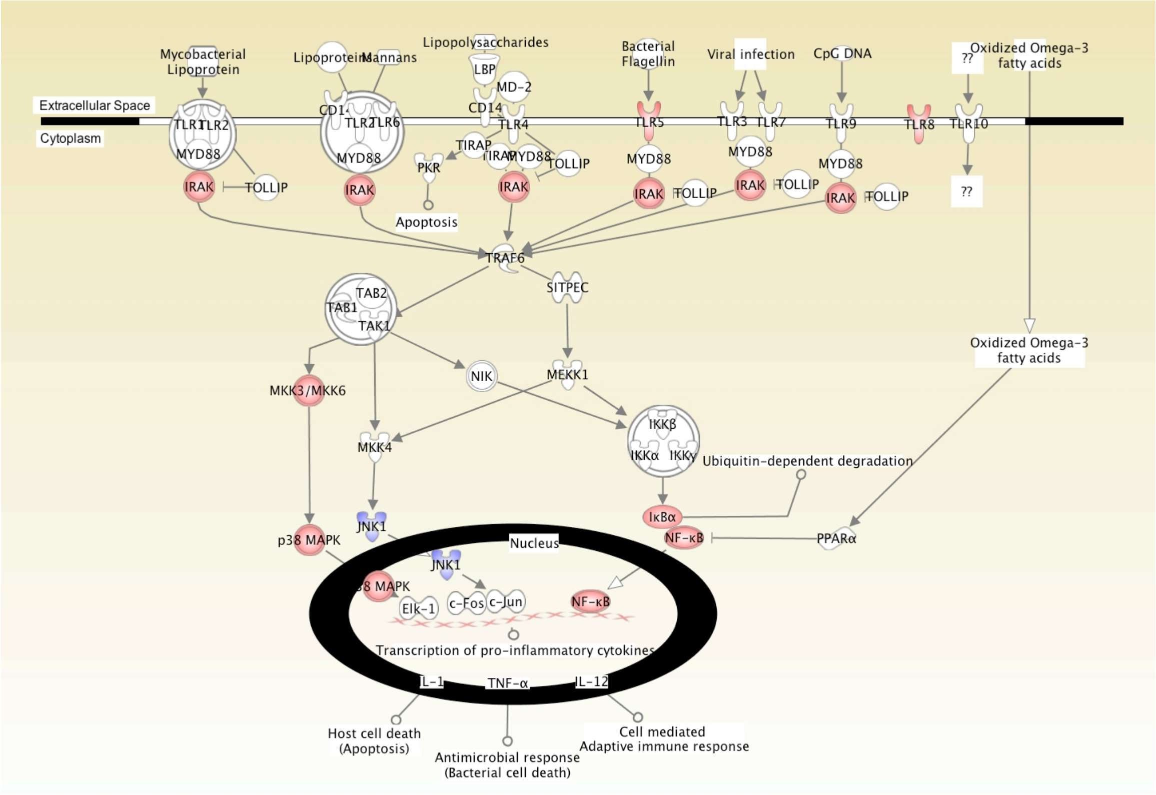

© 2000-2010 Ingenuity Systems, Inc. All rights reserved.

Supplementary Figure 19. The Toll-like receptor (TLR) pathway. Among the top 500 probesets, 12 are in this canonical pathway (representing 9 genes). Those genes in blue and red have negative and positive spearman correlation coefficients between WPEC and ocMOF respectively. JNK1 was removed from further analysis because its correlation was inconsistent with what identified by IPA.
