# Supplementary material for: Dissecting Inflammatory Complications in Critically Injured Patients by Within-Patient Gene Expression Changes: A Longitudinal Clinical Genomics Study
Source: PLoS Med. 2011 Sep 13;8(9):e1001093. doi: 10.1371/journal.pmed.1001093 (PMC3172280; doi:10.1371/journal.pmed.1001093)
Supplement: Figure S21 — The Interleukin (IL)-6 signaling pathway. Among the top 500 probesets, 14 are in this canonical pathway (representing ten genes). Genes in blue and red have negative and positive Spearman correlation coefficients between WPEC and ocMOF, respectively. JNK was removed from further analysis because its correlation was inconsistent with that identified by IPA. (PDF) [file pmed.1001093.s022.pdf]

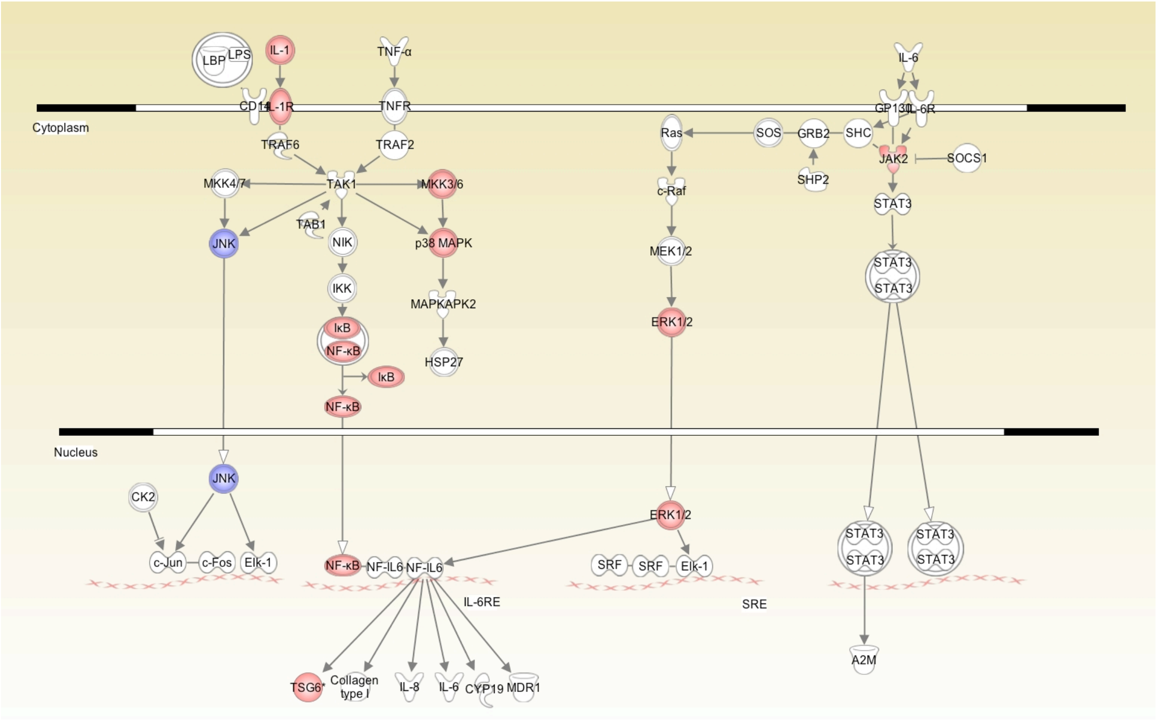

© 2000-2010 Ingenuity Systems, Inc. All rights reserved.

Supplementary Figure 21. The Interleukin (IL)-6 signaling pathway. Among the top 500 probesets, 14 are in this canonical pathway (representing 10 genes). Those genes in blue and red have negative and positive spearman correlation coefficients between WPEC and ocMOF respectively. JNK was removed from further analysis because its correlation was inconsistent with the one identified by IPA.
