# Supplementary material for: Dissecting Inflammatory Complications in Critically Injured Patients by Within-Patient Gene Expression Changes: A Longitudinal Clinical Genomics Study
Source: PLoS Med. 2011 Sep 13;8(9):e1001093. doi: 10.1371/journal.pmed.1001093 (PMC3172280; doi:10.1371/journal.pmed.1001093)
Supplement: Figure S22 — Gene expression profiles of probesets involved in the IL-6 signaling pathway. Similar to p38 MAPK signaling pathway. See Figure S18 for details. Altogether 13 probesets (representing nine genes) were used for this pathway. For (a) the p-value of the Spearman's test <10−15 and for (d) the p-value of the Kruskal-Wallis test is 0.00898. (PDF) [file pmed.1001093.s023.pdf]

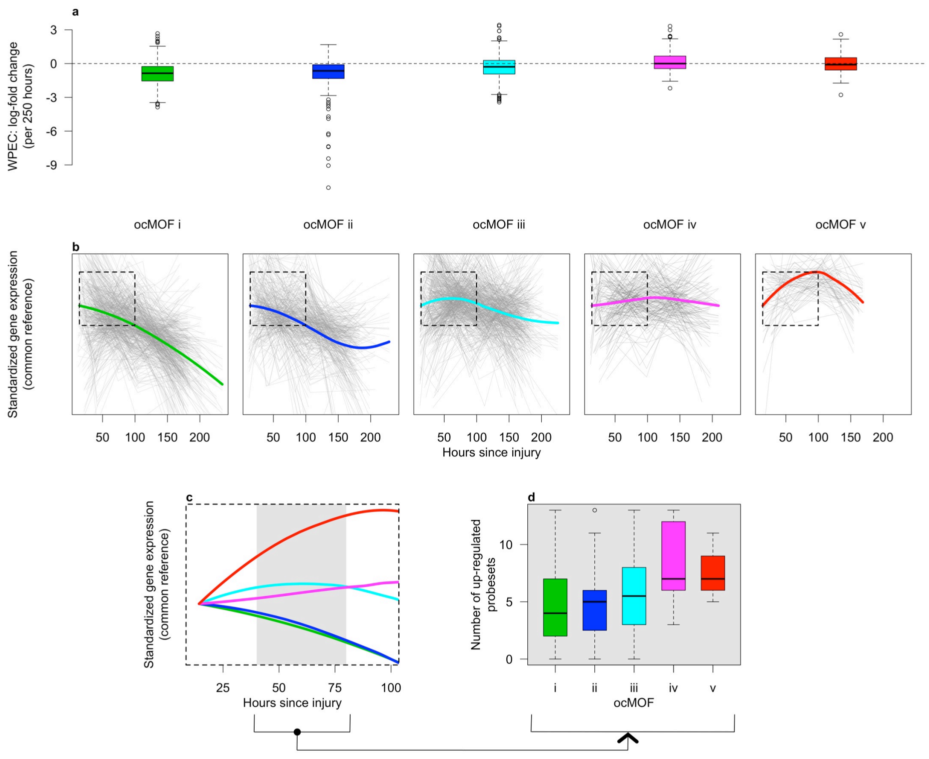

Supplementary Figure 22. Gene expression profiles of probesets involved in the IL-6 signaling pathway. Similar to p38 MAPK signaling pathway. See Supp. Fig. 18 for details. Altogether 13 probesets (representing 9 genes) were used for this pathway. For **a**, the p-value of the Spearman's test $<10^{-15}$ and for **d**, the p-value of the Kruskal-Wallis test is 0.00898.
