# Supplementary material for: Dissecting Inflammatory Complications in Critically Injured Patients by Within-Patient Gene Expression Changes: A Longitudinal Clinical Genomics Study
Source: PLoS Med. 2011 Sep 13;8(9):e1001093. doi: 10.1371/journal.pmed.1001093 (PMC3172280; doi:10.1371/journal.pmed.1001093)
Supplement: Figure S23 — The production of nitric oxide and reactive oxygen species in macrophages pathway. Among the top 500 probesets, 18 are in this canonical pathway (representing 13 genes). Genes in blue and red have negative and positive Spearman correlation coefficients between WPEC and ocMOF, respectively. JNK and PP1/PP2a were removed from further analysis because their correlations were inconsistent with those identified by IPA. IkB and p38 MAPK were removed because of their complexity in this pathway. (PDF) [file pmed.1001093.s024.pdf]

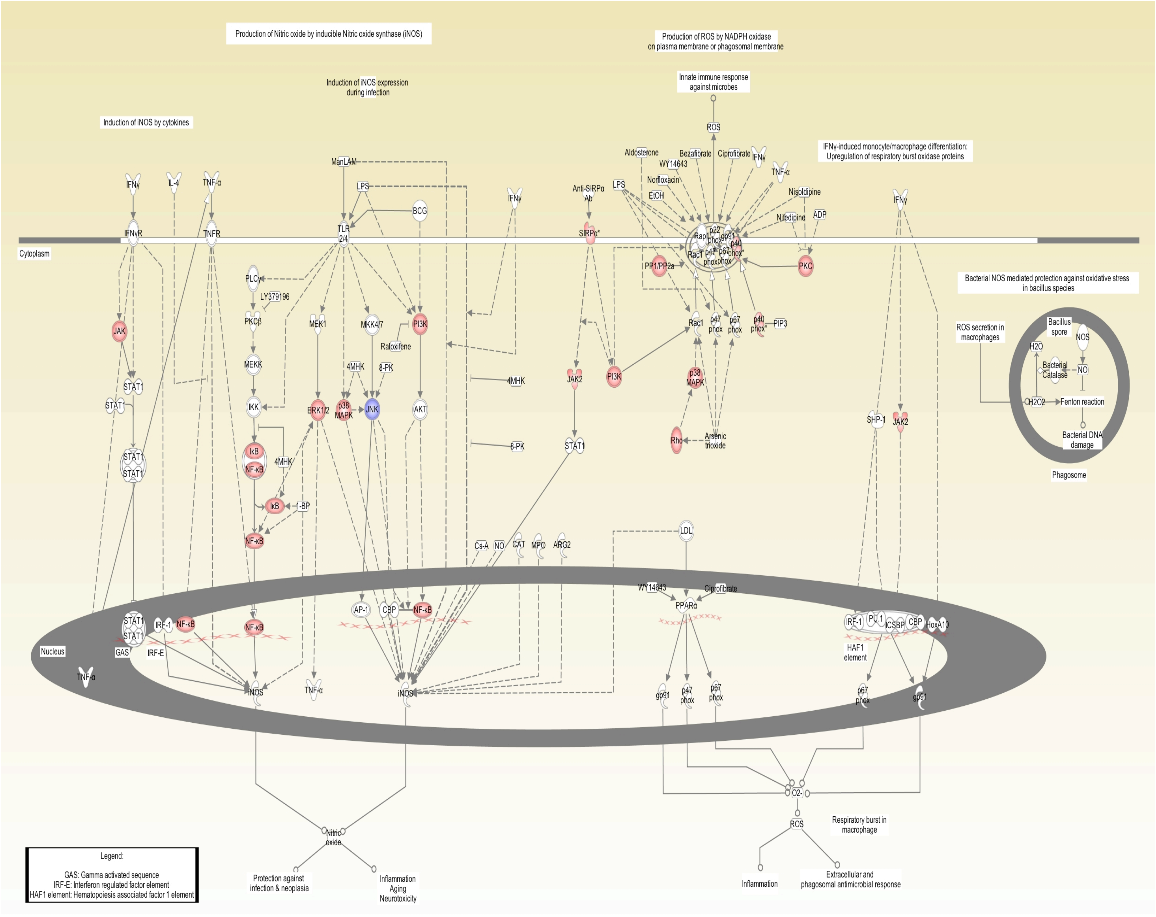

© 2000-2010 Ingenuity Systems, Inc. All rights reserved.

Supplementary Figure 23. The production of nitric oxide and reactive oxygen species in macrophages pathway. Among the top 500 probesets, 18 are in this canonical pathway (representing 13 genes). Those genes in blue and red have negative and positive spearman correlation coefficients between WPEC and ocMOF respectively. JNK and PP1/PP2a were removed from further analysis because their correlations were inconsistent with those identified by IPA. IkB and p38 MAPK were removed because of their complexity in this pathway.
