# Supplementary material for: Dissecting Inflammatory Complications in Critically Injured Patients by Within-Patient Gene Expression Changes: A Longitudinal Clinical Genomics Study
Source: PLoS Med. 2011 Sep 13;8(9):e1001093. doi: 10.1371/journal.pmed.1001093 (PMC3172280; doi:10.1371/journal.pmed.1001093)
Supplement: Figure S24 — Gene expression profiles of probesets involved in the production of nitric oxide and reactive oxygen species in macrophages pathway. Similar to p38 MAPK signaling pathway. See Figure S18 for details. Altogether 11 probesets (representing nine genes) were used for this pathway. For (a) the p-value of the Spearman's test <10−15 and for (d) the p-value of the Kruskal-Wallis test is 0.01370. (PDF) [file pmed.1001093.s025.pdf]

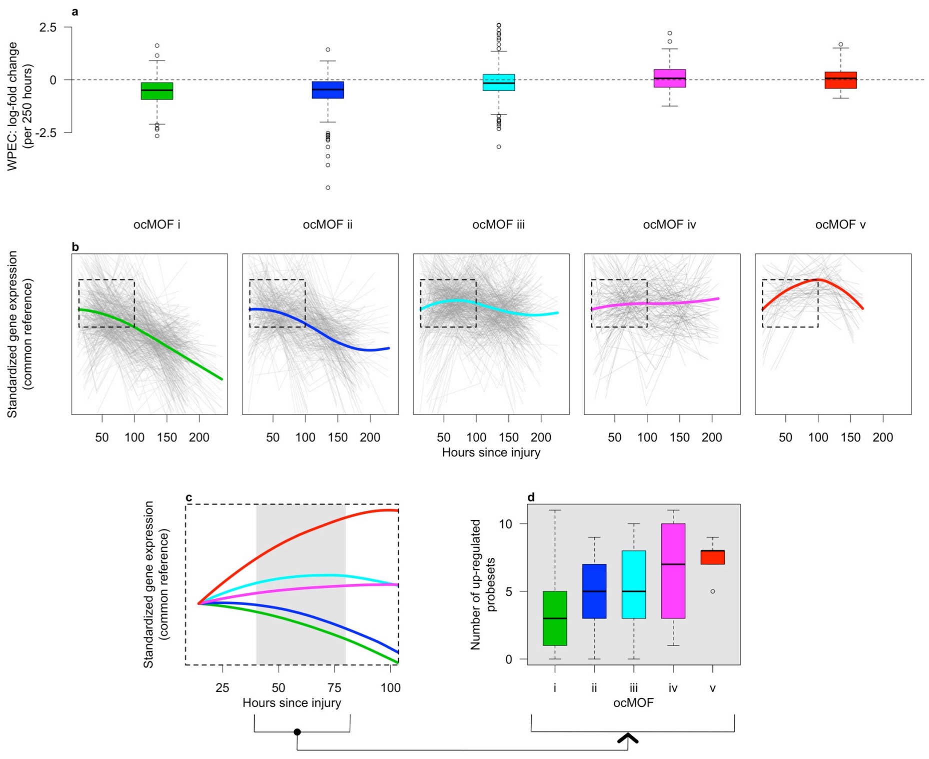


**Supplementary Figure 24. Gene expression profiles of probesets involved in the production of nitric oxide and reactive oxygen species in macrophages pathway.** Similar to p38 MAPK signaling pathway. See Supp. Fig. 18 for details. Altogether 11 probesets (representing 9 genes) were used for this pathway. For **a**, the p-value of the Spearman's test $<10^{-15}$ and for **d**, the p-value of the Kruskal-Wallis test is 0.01370.
