# Supplementary material for: Dissecting Inflammatory Complications in Critically Injured Patients by Within-Patient Gene Expression Changes: A Longitudinal Clinical Genomics Study
Source: PLoS Med. 2011 Sep 13;8(9):e1001093. doi: 10.1371/journal.pmed.1001093 (PMC3172280; doi:10.1371/journal.pmed.1001093)
Supplement: Figure S25 — Gene expression profiles of MHC-II and p38 MAPK in a controlled endotoxin experiment. (a,b) The mean of the log-expression of MHC-II (a) and p38 MAPK (b). The black and red lines correspond to the healthy patients administered with placebo and endotoxin, respectively. After hour 5 (the region to the right of the dotted vertical line), the mean trajectories corresponding to healthy patients administered with endotoxin are similar to the dominant trajectories of ocMOF i and ii. (PDF) [file pmed.1001093.s026.pdf]

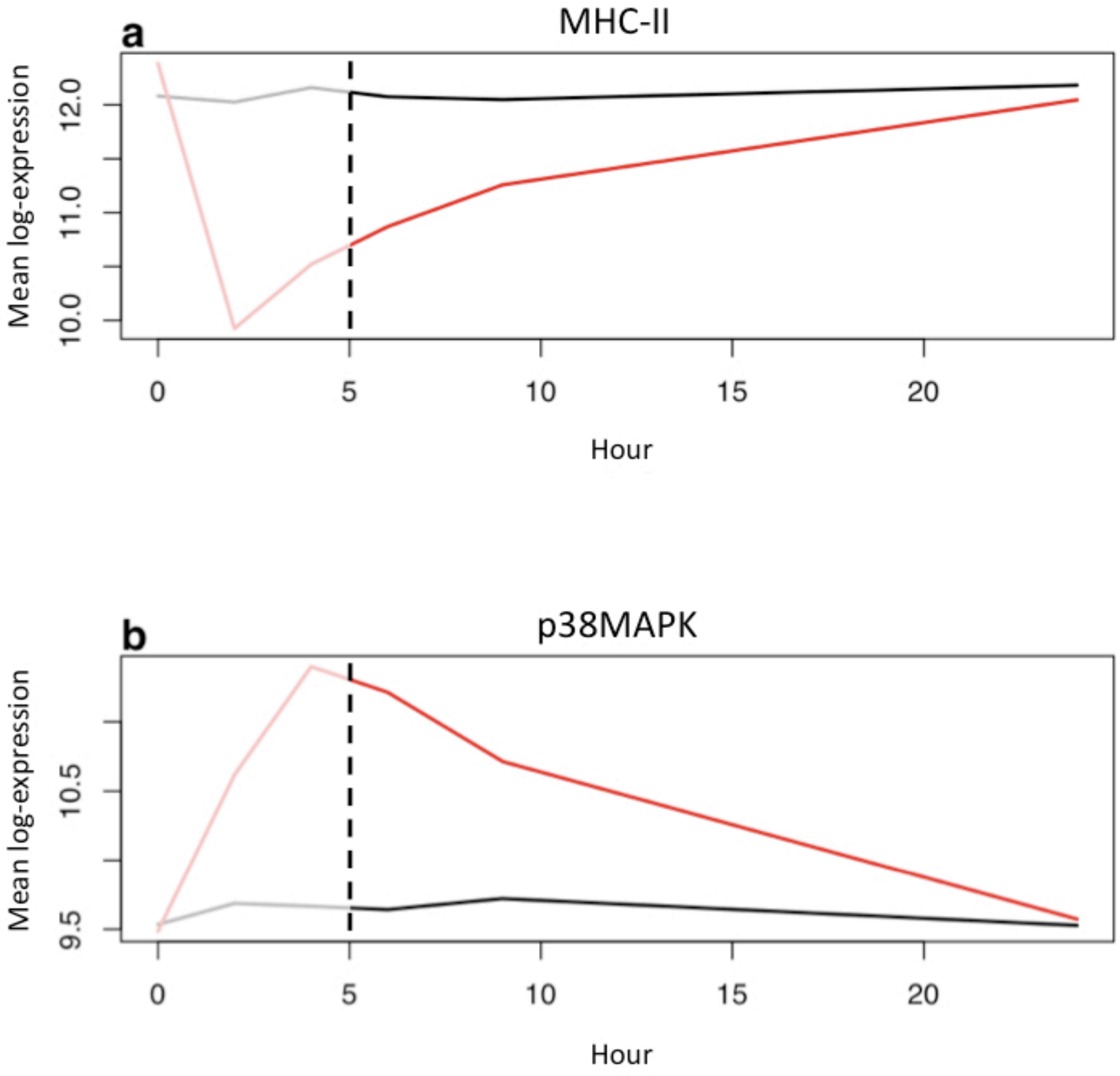

Supplementary Figure 25. Gene expression profiles of MHC-II and p38MAPK in a controlled endotoxin experiment. a-b, The mean of the log-expression of MHC-II (shown in **a**) and p38MAPK (shown in **b**). The black and red lines correspond to the healthy patients administered with placebo and endotoxin, respectively. After hour 5 (the region to the right of the dotted vertical line), the mean trajectories corresponding to healthy patients administered with endotoxin are similar to the dominant trajectories of *ocMOF i* and *ii*.
