# Supplementary material for: Dissecting Inflammatory Complications in Critically Injured Patients by Within-Patient Gene Expression Changes: A Longitudinal Clinical Genomics Study
Source: PLoS Med. 2011 Sep 13;8(9):e1001093. doi: 10.1371/journal.pmed.1001093 (PMC3172280; doi:10.1371/journal.pmed.1001093)
Supplement: Table S1 — The ten most significant clinical variables (out of 393) associated with the eight principal components from the WPEC matrix. Legend: *Clinical variables are treated as categorical variables, and R2 corresponds to McFadden's pseudo R2. (PDF) [file pmed.1001093.s027.pdf]

Rank	Clinical variables	P-value	Q-value	R^2
1	ocMOF*	$5.6x10^{-8}$	$5.0x10^{-6}$	0.26
2	Respiratory component of Marshall score on day 6*	$2.3x10^{-5}$	$1.0x10^{-3}$	0.25
3	Hepatic component of Marshall score on day 3*	$5.7x10^{-5}$	$1.7x10^{-3}$	0.24
4	Denver score on day 6	$7.9x10^{-5}$	$1.8x10^{-3}$	0.24
5	Worst cardio score over days 2-28	$1.3x10^{-4}$	$2.1x10^{-3}$	0.23
6	Denver score on day 3*	$1.4x10^{-4}$	$2.1x10^{-3}$	0.22
7	Denver score on day 5*	$2.4x10^{-4}$	$3.1x10^{-3}$	0.22
8	Marshall score on day 6	$4.1x10^{-4}$	$4.6x10^{-3}$	0.21
9	Initial white blood cell count	$4.8x10^{-4}$	$4.8x10^{-3}$	0.22
10	First day in full recovery from cardio	$7.3x10^{-4}$	$5.3x10^{-3}$	0.21

Table S1. The ten most significant clinical variables (out of 393) associated with the eight principal components from the WPEC matrix.
Legend: *Clinical variables are treated as categorical variables, and R^2 corresponds to McFadden's pseudo R^2.
