# Supplementary material for: Dissecting Inflammatory Complications in Critically Injured Patients by Within-Patient Gene Expression Changes: A Longitudinal Clinical Genomics Study
Source: PLoS Med. 2011 Sep 13;8(9):e1001093. doi: 10.1371/journal.pmed.1001093 (PMC3172280; doi:10.1371/journal.pmed.1001093)
Supplement: Table S2 — The ten most significant clinical variables (out of 393) associated with the eight principal components from mean expression matrix. Legend: *Clinical variables are treated as categorical variables, and R2 corresponds to McFadden's pseudo R2. (PDF) [file pmed.1001093.s028.pdf]

**Rank**	Clinical variables	P-value	Q-value	R^2
1	Sampling phase*	0	0	1.00
2	Renal component of Marshall score on day 5*	$3.7x10^{-8}$	$1.1x10^{-6}$	0.38
3	Highest serum creatinine on day 6	$7.8x10^{-8}$	$1.5x10^{-6}$	0.42
4	Highest serum creatinine on day 3	$1.3x10^{-7}$	$1.6x10^{-6}$	0.35
5	Highest serum creatinine on day 4	$1.4x10^{-7}$	$1.6x10^{-6}$	0.37
6	First discharge day from ICU since injury	$2.4x10^{-7}$	$2.2x10^{-6}$	0.32
7	Trauma center*	$5.2x10^{-7}$	$4.3x10^{-6}$	0.34
8	Lowest Hemoglobin on day 3	$7.9x10^{-7}$	$5.2x10^{-6}$	0.33
9	Highest serum creatinine on day 5	$8.2x10^{-7}$	$5.2x10^{-6}$	0.35
10	Marshall score on day 3	$2.0x10^{-6}$	$1.1x10^{-5}$	0.29

Table S2. The ten most significant clinical variables (out of 393) associated with the eight principal components from mean expression matrix.
Legend: *Clinical variables are treated as categorical variables, and R^2 corresponds to McFadden's pseudo R^2.
