# Supplementary material for: Dissecting Inflammatory Complications in Critically Injured Patients by Within-Patient Gene Expression Changes: A Longitudinal Clinical Genomics Study
Source: PLoS Med. 2011 Sep 13;8(9):e1001093. doi: 10.1371/journal.pmed.1001093 (PMC3172280; doi:10.1371/journal.pmed.1001093)
Supplement: Table S5 — The top five canonical statistically significant (p-value<0.002, after Bonferroni correction) pathways for the five dynamic co-expression modules. For each canonical pathway we report the p-value of Fisher's exact test that ascertains enrichment and the proportion of genes in the pathway that were actually in the module within the brackets, and the gene names (in italics). Note that N.A. denotes no significant pathways with three or more genes were identified. (PDF) [file pmed.1001093.s031.pdf]

Rank	Module A	Module B	Module C	Module D	Module E
1	Oxidative Phosphorylation (5.3x10^{-5}/0.024) *ATP6V1C1, ATP6V0D1, ATP5L, ATP6V0E1*	N.A.	RAN Signaling (1.7x10^{-5}/0.217) *KPNA5, KPNA4, TNPO1, XPO1, KPNA1*	Allograft Rejection Signaling (1.2x10^{-12}/0.104) *HLA-DQB1, HLA-DOA, HLA-DRB4, HLA-DRA, HLA-DQA1, HLA-DRB1, HLA-DMB, HLA-DPB1, HLA-DPA1, HLA-F*	Protein Ubiquitination Pathway (3.3x10^{-8}/0.075) USP12, USP15, UBE2A, USP38, USP36, USP54, UBE2D1, UBE2F, UBE4A, UBE2G2, USP32, USP4, UBE2G1, UBE2E2, USP9Y
2	N.A.	N.A.	IL-10 Signaling (4.4x10^{-5}/0.114) *IL1R2, MAP2K6, IL10RB, IL10RA, MAP4K4, MAPK13, IL1R1, CHUK*	Antigen Presentation Pathway *(3.8x10^{-12}/0.231)* *HLA-DOA, HLA-DRB4, HLA-DRA, HLA-DQA1,HLA-DRB1, HLA-DMB, HLA-DPB1, HLA-DPA1, HLA-F*	Hypoxia Signaling in the Cardiovascular System (2.7x10^{-5}/0.1) *UBE2G2, UBE2A, UBE2G1, UBE2E2, CREB5, UBE2D1, UBE2F*
3	N.A.	N.A.	IL-6 Signaling (6.0x10^{-4}/0.086) *IL1R2, MAP2K6, MAP2K2, SOS2, MAP4K4, MAPK13, IL1R1, CHUK*	Cytotoxic T Lymphocyte-mediated Apoptosis of Target Cells (3.9x10^{-12}/0.116) HLA-DQB1, HLA-DOA, HLA-DRB4, HLA-DRA, HLA-DQA1, HLA-DRB1, HLA-DMB, HLA-DPB1, HLA-DPA1, HLA-F	Cleavage and Polyadenylation of Pre-mRNA (5.4x10^{-4}/0.25) *CPSF2, CPSF6, WDR33*
4	N.A.	N.A.	Glycosphingolipid Biosynthesis – Lactoseries (1.9x10^{-3}/0.111) *ST3GAL2, ST3GAL3, ST3GAL4*	OX40 Signaling Pathway (1.4x10^{-11}/0.105) *HLA-DQB1, HLA-DOA, HLA-DRB4, HLA-DRA, HLA-DQA1, HLA-DRB1, HLA-DMB, HLA-DPB1, HLA-DPA1, HLA-F*	N.A.
5	N.A.	N.A.	N.A.	Nur77 Signaling in T Lymphocytes (1.9x10^{-8}/0.125) *HLA-DQB1, HLA-DOA, HLA-DRA, HLA-DQA1, HLA-DRB1, SIN3B, HLA-DMB, SIN3A*	N.A.

Table S5. The top five canonical statistically significant (p-value <0.002, after Bonferroni correction) pathways for the five dynamic co-expression modules.
For each canonical pathway we report the p-value of Fisher's exact test that ascertains enrichment and the proportion of genes in the pathway that were actually in the module within the brackets, and the gene names (in italics). Note that N.A. denotes no significant pathways with three or more genes were identified.
