# Supplementary material for: Dissecting Inflammatory Complications in Critically Injured Patients by Within-Patient Gene Expression Changes: A Longitudinal Clinical Genomics Study
Source: PLoS Med. 2011 Sep 13;8(9):e1001093. doi: 10.1371/journal.pmed.1001093 (PMC3172280; doi:10.1371/journal.pmed.1001093)
Supplement: Table S6 — The top 20 canonical pathways for the top 500 probesets from WPEC and ocMOF association analysis (IPA, obtained May 2010). Legend: p, p-value of Fisher's exact test for ascertaining enrichment; and ratio, the proportion of genes in the pathway that are in the top 500. (PDF) [file pmed.1001093.s032.pdf]

Canonical Pathways	-log(p)	Ratio	Genes
Dendritic Cell Maturation	6.54	0.0862	HLA-DQB1,PIK3CG,MAPK1,MAPK8,JAK2,CD1C,NFKB1,HLA-DRA,HLA-DMB,NFKBIA,MAPK14,HLA-DRB1,CREB5,HLA-DQA1,IL1RN
Toll-like Receptor Signaling	6.19	0.167	TLR5,IRAK3,NFKBIA,IRAK2,MAPK14,MAPK8,MAP2K6,NFKB1,TLR8 (includes EG:51311)
p38 MAPK Signaling	5.45	0.113	MAX,TRADD,IRAK3,IRAK2,MAPK14,IL1R1,MAP2K6,CREB5,TIFA,MEF2C,IL1RN
IL-6 Signaling	4.81	0.108	NFKBIA,MAPK14,MAPK1,IL1R1,MAPK8,JAK2,MAP2K6,IL1RN,NFKB1,TNFAIP6
Production of Nitric Oxide and Reactive Oxygen Species in Macrophages	4.66	0.0703	PIK3CG,MAPK1,PRKCD,MAPK8,JAK2,NCF4,NFKB1,PPP1R12A,NFKBIA,SIRPA,MAPK14,RHOT1,JAK3
Antigen Presentation Pathway	4.65	0.154	HLA-DRB1,HLA-DPA1,HLA-DPB1,HLA-DQA1,HLA-DRA,HLA-DMB
CD40 Signaling	4.48	0.119	PIK3CG,NFKBIA,MAPK14,MAPK1,MAPK8,MAP2K6,JAK3,NFKB1
IL-10 Signaling	4.43	0.114	NFKBIA,MAPK14,IL1R1,IL10RB,MAPK8,MAP2K6,IL1RN,NFKB1
Type I Diabetes Mellitus Signaling	4.4	0.087	TRADD,NFKBIA,MAPK14,IL1R1,MAPK8,JAK2,MAP2K6,HLA-DPB1,NFKB1,HLA-DMB
B Cell Receptor Signaling	4.32	0.0779	PIK3CG,NFKBIA,BCL2A1,MAPK14,MAPK1,MAPK8,MAP2K6,CREB5,CAMK2D,BCL6,NFKB1,PIK3AP1
Role of Macrophages, Fibroblasts and Endothelial Cells in Rheumatoid Arthritis	4.26	0.0528	TLR5,PIK3CG,IRAK3,PRKCD,MAPK1,IL1R1,MAP2K6,JAK2,IL18R1,CAMK2D,NFKB1,TLR8 (includes EG:51311),TRADD,NFKBIA,IRAK2,MAPK14,CREB5,IL1RN
IL-4 Signaling	4.22	0.108	HLA-DQB1,PIK3CG,HLA-DRB1,JAK2,JAK3,HLA-DQA1,HLA-DRA,HLA-DMB
LPS-stimulated MAPK Signaling	3.98	0.103	PIK3CG,NFKBIA,MAPK14,PRKCD,MAPK1,MAPK8,MAP2K6,NFKB1
IL-1 Signaling	3.91	0.0849	IRAK3,NFKBIA,IRAK2,MAPK14,IL1R1,MAPK8,MAP2K6,GNB1,NFKB1
Nicotinate and Nicotinamide Metabolism	3.66	0.0662	VNN1,BST1,CSNK1D,PRKCD,MAPK1,MAPK8,MAP2K6,NAMPT,VNN2
Acute Phase Response Signaling	3.64	0.0674	TRADD,PIK3CG,NFKBIA,SOD2,MAPK14,MAPK1,IL1R1,MAPK8,JAK2,MAP2K6,IL1RN,NFKB1
IL-22 Signaling	3.63	0.179	MAPK14,MAPK1,STAT5B,IL10RB,MAPK8
B Cell Development	3.63	0.132	HLA-DQB1,HLA-DRB1,HLA-DQA1,HLA-DRA,HLA-DMB
IL-15 Signaling	3.57	0.104	PIK3CG,MAPK14,MAPK1,STAT5B,JAK2,JAK3,NFKB1
Erythropoietin Signaling	3.35	0.0921	PIK3CG,NFKBIA,PRKCD,MAPK1,STAT5B,JAK2,NFKB1

Table S6. The top 20 canonical pathways for the top 500 probesets from WPEC and ocMOF association analysis (IPA, obtained May 2010).
Legend: p, p-value of Fisher's exact test for ascertaining enrichment; and ratio, the proportion of genes in the pathway that are in the top 500.
