# Supplementary material for: Dissecting Inflammatory Complications in Critically Injured Patients by Within-Patient Gene Expression Changes: A Longitudinal Clinical Genomics Study
Source: PLoS Med. 2011 Sep 13;8(9):e1001093. doi: 10.1371/journal.pmed.1001093 (PMC3172280; doi:10.1371/journal.pmed.1001093)
Supplement: Table S7 — Detailed results of DAVID analysis on the top 500 probesets from WPEC and ocMOF association analysis. (PDF) [file pmed.1001093.s033.pdf]

Gene Group 1	Enrichment Score: 3.43
AFFYMETRIX_3PRIME_IVT_ID	Gene Name
212671_s_at	similar to hCG2042724; similar to HLA class II histocompatibility antigen, DQ(1) alpha chain precursor (DC-4 alpha chain); major histocompatibility complex, class II, DQ alpha 1
201137_s_at	major histocompatibility complex, class II, DP beta 1
205987_at	CD1c molecule
204670_x_at, 209312_x_at	major histocompatibility complex, class II, DR beta 4; major histocompatibility complex, class II, DR beta 1
221491_x_at, 215193_x_at	major histocompatibility complex, class II, DR beta 3
208894_at	major histocompatibility complex, class II, DR alpha
212998_x_at, 209823_x_at, 212999_x_at, 211654_x_at, 211656_x_at	major histocompatibility complex, class II, DQ beta 1; similar to major histocompatibility complex, class II, DQ beta 1
203932_at	major histocompatibility complex, class II, DM beta
211991_s_at, 213537_at, 211990_at	major histocompatibility complex, class II, DP alpha 1

Gene Group 2	Enrichment Score: 0.99
AFFYMETRIX_3PRIME_IVT_ID	Gene Name
222981_s_at	RAB10, member RAS oncogene family
219210_s_at	RAB8B, member RAS oncogene family
209882_at	Ras-like without CAAX 1
202101_s_at	v-ral simian leukemia viral oncogene homolog B (ras related; GTP binding protein)
223831_x_at	RAB43, member RAS oncogene family; hypothetical LOC100131426

Gene Group 3	Enrichment Score: 0.51
AFFYMETRIX_3PRIME_IVT_ID	Gene Name
206618_at	interleukin 18 receptor 1
202948_at	interleukin 1 receptor, type I
211102_s_at	leukocyte immunoglobulin-like receptor, subfamily A (with TM domain), member 2
202897_at, 202896_s_at	signal-regulatory protein alpha
220000_at	sialic acid binding Ig-like lectin 5
Gene Group 4	Enrichment Score: 0.44
AFFYMETRIX_3PRIME_IVT_ID	Gene Name
235568_at	chromosome 19 open reading frame 59
226841_at, 226818_at	macrophage expressed 1
240310_at, 1560724_at	torsin A interacting protein 1
218728_s_at, 223993_s_at, 228437_at	cornichon homolog 4 (Drosophila)
235245_at	transmembrane protein 92
212090_at	glutamate receptor, ionotropic, N-methyl D-aspartate-associated protein 1 (glutamate binding)
209551_at	Yip1 domain family, member 4
1555728_a_at	membrane-spanning 4-domains, subfamily A, member 4
227052_at	chromosome 4 open reading frame 34
220945_x_at	MANSC domain containing 1
225462_at	transmembrane protein 128
213173_at, 213159_at	pecanex homolog (Drosophila)
203437_at	transmembrane protein 11
226529_at	transmembrane protein 106B

222995_s_at, 232053_x_at	rhomboid domain containing 2

Gene Group 5	Enrichment Score: 0.23
AFFYMETRIX_3PRIME_IVT_ID	Gene Name
224782_at	zinc finger, matrin type 2
238493_at	zinc finger protein 506
226344_at	zinc finger, matrin type 1
221844_x_at	zinc finger protein 574
226680_at	IKAROS family zinc finger 5 (Pegasus)
229743_at	zinc finger protein 438
227150_at, 205322_s_at	metal-regulatory transcription factor 1
214686_at	zinc finger protein 266
218312_s_at, 217593_at	zinc finger and SCAN domain containing 18
228460_at	zinc finger protein 319

Table S7. Detailed results of DAVID analysis on the top 500 probesets from WPEC and ocMOF association analysis.
