# Supplementary material for: Dissecting Inflammatory Complications in Critically Injured Patients by Within-Patient Gene Expression Changes: A Longitudinal Clinical Genomics Study
Source: PLoS Med. 2011 Sep 13;8(9):e1001093. doi: 10.1371/journal.pmed.1001093 (PMC3172280; doi:10.1371/journal.pmed.1001093)
Supplement: Text S1 — Inclusion/exclusion criteria and gene expression information. (PDF) [file pmed.1001093.s034.pdf]

Text S1. Inclusion/Exclusion Criteria and Gene Expression Information

Study design

Our trauma patient population is defined by the following inclusion/exclusion criteria.

Inclusion Criteria

- Patient has blunt trauma without isolated head injury
- Absence of traumatic brain injury defined as AIS head <4 or GCS motor >3 within 24 hours of injury
- Emergency Department (ED) arrival ≤6 hours from time of injury
- Blood transfusion within 12 hours of injury
- Base deficit ≥6 or systolic blood pressure <90 mmHg within 60 minutes of ED arrival
- Fully or partially intact cervical spinal cord

Exclusion Criteria

- Age < 16
- Anticipated survival of <24 hours from time of injury
- Anticipated survival <28 days due to pre-existing medical condition
- Inability to obtain initial blood draw within first 12 hours after injury
- Traumatic brain injury, i.e. GCS ≤ 8 after ICU admission AND brain computerized tomography scan abnormality within first 12 hours after injury
- Inability to obtain informed consent
- Pre-existing, ongoing immunosuppression - e.g. transplant recipient
- Pre-existing, ongoing immunosuppression - e.g. chronic high dose corticosteroids (>20 mg/prednisone-equivalents/day)
- Pre-existing, ongoing immunosuppression - oncolytic drug(s) therapy within the past 14 days
- Pre-existing, ongoing immunosuppression - HIV positive AND CD4 count < 200 cells/mm3
- Possible requirement for early immunosuppression - e.g. significant likelihood of requiring high dose corticosteroids (e.g. spinal cord injury)
- Significant pre-existing organ dysfunction - lung: currently receiving home oxygen therapy, as documented in the medical records
- Significant pre-existing organ dysfunction - heart: congestive heart failure, as documented in the medical records
- Significant pre-existing organ dysfunction - renal: chronic renal failure (creatinine >2 μmol/L)
- Significant pre-existing organ dysfunction - liver: cirrhosis with portal hypertension or encephalopathy

Among the 168 patients, 7 patients experienced death. One patient died from severe head injuries and was subsequently removed from the analysis. The primary causes of death among the remaining six patients who experienced death are: Hypovolemic shock (1 patient), Sepsis (1 patient), Hypoxia (1 patient), Brain Death (1 patient), Multiple Organ Failure (2 patients).

Longitudinal gene expression

Detailed protocols used for obtaining and processing the total blood leukocytes and for array hybridization are described elsewhere[1,2]. Briefly, blood leukocytes were lysed and total cellular RNA extracted (Qiagen RNeasy Midi Kit Cat 75142), and the resulting cellular RNA was hybridized onto an Affymetrix HU133 Plus 2.0 GeneChip™ according to the manufacturer's recommendations. A total of 797 arrays were used to measure the longitudinal gene expression. Intended sampling was on days 0, 1, 4, 7, 14, 21 and 28 since injury, but depending on the total days from injury to discharge/death, the number of sampling points per patient was 2-7 (Supp. Fig. 1). To model early expression changes, we focused on samples collected ≤250 hours and meeting the RNA quality requirements, giving 604 arrays. We performed normalization and probe-set summarization, taking into account four separate sampling and processing phases. All arrays for a given patient were always collected

and processed in the same sampling phase. We used the DNA-chip Analyzer[3] to compute probe-set specific mRNA levels from the CEL files, which performs an invariant set normalization and computes model-based expression to pool information across multiple arrays. While taking the $\log_2$ of gene expression, we added a small constant, 10, to each measurement for variance stabilization of low values[4].

References

1. Cobb JP, Mindrinos MN, Miller-Graziano C, Calvano SE, Baker HV, et al. (2005) Application of genome-wide expression analysis to human health and disease. Proc Natl Acad Sci USA 102: 4801-4806.
2. Feezor RJ, Baker HV, Mindrinos M, Hayden D, Tannahill CL, et al. (2004) Whole blood and leukocyte RNA isolation for gene expression analyses. Physiol Genomics 19: 247-254.
3. Li C, Wong WH (2001) Model-based analysis of oligonucleotide arrays: expression index computation and outlier detection. Proc Natl Acad Sci USA 98: 31-36.
4. Storey JD, Xiao W, Leek JT, Tompkins RG, Davis RW (2005) Significance analysis of time course microarray experiments. Proc Natl Acad Sci USA 102: 12837-12842.
