# Supplementary material for: Dissecting Inflammatory Complications in Critically Injured Patients by Within-Patient Gene Expression Changes: A Longitudinal Clinical Genomics Study
Source: PLoS Med. 2011 Sep 13;8(9):e1001093. doi: 10.1371/journal.pmed.1001093 (PMC3172280; doi:10.1371/journal.pmed.1001093)
Supplement: Text S2 — Additional details on statistical framework. (PDF) [file pmed.1001093.s035.pdf]

Text S2. Additional Details on Statistical Framework

Statistical analysis framework

We present a mathematical abstraction of our proposed framework (Fig. 2 of main paper) as a flow chart, and provide a detailed description of the chart. Additional descriptions specific to its application to IHRI and the R computer code that implements the statistical analyses are available in subsequent subsections.

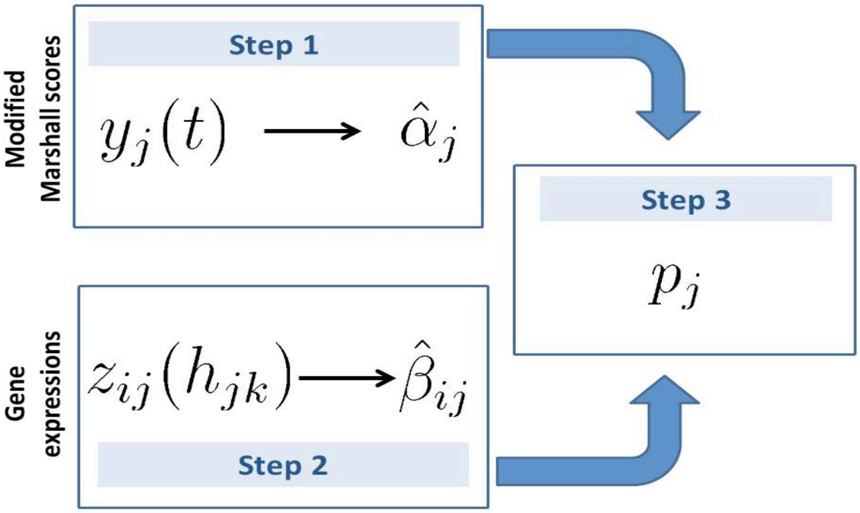

We denoted modified Marshall scores and gene expressions as follows:

- $y_j(t)$: Modified Marshall score of j-th patient at day t, where $j = 1, 2, \ldots, 168,$ and $t \in \{0, 2, 3, \ldots, 28\}$.
- $z_{ij}(h_{jk})$: Gene expression of i-th gene and j-th patient at hour h_{jk}, where $i = 1, 2, \ldots, 54675,$ $j = 1, 2, \ldots, 168,$ $h_{jk} \in [12, 250],$ $k = 1, 2, \ldots, k(j),$ and $k(j) \geq 3$.

Step 1. We performed hierarchical clustering on $y_j(t)$, imputing missing scores with the k-nearest neighbor to get ocMOF, where $j = 1, 2, \ldots, 168,$ and $t \in \{0, 2, 3, \ldots, 20\}$ (see *STEP 1: Longitudinal measure of developing MOF* for more details w.r.t. its application to IHRI):

- $\hat{\alpha}_j$: ocMOF of j-th patient, for $j = 1, 2, \ldots, 126.$

Step 2. We fitted the following linear regression model on the gene expression data:

$$\log\{z_{ij}(h_{jk}) + 10\} = \beta^0_{ij} + \beta_{ij} h_{jk} + \epsilon_{ij}(h_{jk}), \qquad \text{(regression model 1)}$$

where $h_{jk} \in [12, 250],$ $k = 1, 2, \ldots, k(j),$ $i = 1, 2, \ldots, 54675,$ and $j = 1, 2, \ldots, 126,$ to estimate WPEC:

- $\hat{\beta}_{ij}$: WPEC measure of i-th gene and j-th patient, for $i = 1, 2, \ldots, 54675,$ and $j = 1, 2, \ldots, 126.$

Step 3. We performed an adjusted Spearman correlation test between $\hat{\beta}_{ij}$ and $\hat{\alpha}_j$. To account for confounders when testing for association, we fitted the following linear

model (see *STEP 3: Adjusted Spearman rank-based correlation test* for more details w.r.t. its application to IHRI):

$$\hat{\beta}_{ij} = \gamma_0 + \sum_{g=1}^{4} \gamma_g 1\{\hat{\alpha}_j = g\} + \sum_{l \in \Lambda} \gamma_l x_{jl} + \epsilon_{ij}, \qquad \text{(regression model 2)}$$

where Λ is the set of parameter indices that do not correspond to ocMOF, x_{jl}'s are the confounding variables corresponding to the j-th patient, for $i = 1, 2, \ldots, 54675,$ $j = 1, 2, \ldots, 126,$ and $\Lambda = \{5, 6, \ldots, n(\Lambda) + 4\}$. We subtracted from $\hat{\beta}_{ij}$ the fitted variations that are not attributed to ocMOF:

$$\tilde{\beta}_{ij} = \hat{\beta}_{ij} - \hat{\gamma}_0 - \sum_{l \in \Lambda} \hat{\gamma}_l x_{jl}.$$

The values of $\hat{\alpha}_j$ and $\tilde{\beta}_{ij}$ are converted to ranks r_j^a and r_{ij}^b, and the adjusted Spearman correlation coefficient is computed from these ranks:

$$\rho_i = \frac{\sum_{j=1}^{126} (r_j^a - \bar{r}^a)(r_{ij}^b - \bar{r}_i^b)}{\sqrt{\sum_{j=1}^{126} (r_j^a - \bar{r}^a)^2 \sum_{j=1}^{126} (r_{ij}^b - \bar{r}_i^b)^2}},$$

where $\bar{r}^a = \sum_{j=1}^{126} r_j^a / 126$ and $\bar{r}_i^b = \sum_{j=1}^{126} r_{ij}^b / 126.$

To generate the null distribution of ρ_i, we first computed the residuals from regression model 2:

$$\hat{\epsilon}_{ij} = \hat{\beta}_{ij} - \hat{\gamma}_0 - \sum_{g=1}^{4} \hat{\gamma}_g 1\{\hat{\alpha}_j = g\} - \sum_{l \in \Lambda} \hat{\gamma}_l x_{jl}.$$

These residuals are resampled B=200 times. At each b-th resampling iteration, we resampled (without replacement) the residuals on a gene-by-gene basis. These resampled residuals are denoted as $\hat{\epsilon}_{ij}^b$'s, for $b = 1, 2, \ldots, B,$ and simulate the null scenario (i.e. no association between $\hat{\beta}_{ij}$ and $\hat{\alpha}_j$). Therefore, by computing the adjusted Spearman correlation coefficient between $\hat{\epsilon}_{ij}^b$ and $\hat{\alpha}_j$, we obtain 54675 null statistics for each resampling iteration.

By pooling ρ_i^b's across genes and resampling iterations, we obtained the resampled null distribution of the adjusted Spearman correlation coefficient. Hence the p-value for each ρ_i can be computed:

$$p_i = \sum_{l=1}^{54675} \sum_{b=1}^{B} 1\{|\rho_l^b| \geq |\rho_i|\} / (54675 \times B),$$

where $i = 1, 2, \ldots, 54675$. From this step we obtain:

- p_i: resampled p-value of i-th gene, for $i = 1, 2, \ldots, 54675.$

The histogram of these p-values is shown in Fig. 4a of main paper. We next performed false discovery rate calculations on these 54675 p-values in order to identify statistically significant probesets. The number of statistically significant probesets at various FDR cut-offs are provided in Fig. 4b of main paper.

STEP 1: Longitudinal measure of developing MOF

The reasons for missing modified Marshall (neurological component excluded) scores vary across patients, for example, death, discharge, or transfer to some facility. In addition, more than 50% of the patients had missing modified Marshall scores after

day 20 since injury. Therefore, we used only the partial trajectories from day 0 to 20 and imputed the remaining missing entries using the k-nearest neighbor (k-NN) approach, where k=10 [1]. Using the Euclidean distance as the dissimilarity metric and Ward as the agglomeration method, we performed hierarchical clustering on the modified Marshall scores collected from day 0 to 20, with missing scores imputed via k-NN and obtained five clusters/subgroups (see Text S5). To obtain the ordering of the five clusters, we used clinically relevant variables, such as the 28 day mortality and the proportion of ICU free days. From these variables, we computed subgroup-specific summary statistics (e.g. proportion of 28 day mortality and mean ICU fee days) and used them to rank the subgroups.

STEP 2: Longitudinal gene expression

To model early expression changes, we focused on samples collected ≤250 hours and meeting the RNA quality requirements, giving 604 arrays. There were originally 129 patients with ≥3 arrays meeting the RNA quality requirements among hours 12-250. The first 12 hours were excluded because of different gene expression dynamics from subsequent hours, which we found to be an informative result in itself (see Text S3 for the technical details and Text S6 for the result). We removed two further patients (*ocMOF i* and *iv*) due to data quality issues (Supp. Fig. 2) and one due to death from head injury (*ocMOF iv*). Thus, we considered the longitudinal gene expression data of 126 patients, where 38, 28, 42, 13, and 5 patients were in *ocMOF i* to *v* respectively.

STEP 3: Adjusted Spearman rank-based correlation test

We fitted the following linear model

WPEC = ocMOF + batch + sex + noise,

where ocMOF, batch (i.e., sampling and processing phase) and sex were coded as factors. Next, we subtracted the variation fitted to the batch and sex variables from the WPEC matrix but kept the fitted variation of ocMOF to obtain adjusted WPEC values, which were then used to calculate probeset-specific adjusted Spearman's rank correlations. We converted adjusted correlations to p-values through a resampling-based null distribution obtained by resampling the residuals (regressing out ocMOF, sampling phase and sex) and recalculating adjusted Spearman correlations.

Note that the ocMOF-WPEC association analysis reported in the paper includes sampling phase and gender as the adjustment variables. The following observations motivated the choice of sampling phase: (i) the microarrays were processed and subsequently normalized in four separate batches, and hence sampling phase is a major source of technical variation, both static and dynamic; and (ii) there is a statistically significant correlation between sampling phase and ocMOF (p-value 0.004, Table 1 of main paper), suggesting that sampling phase could be a potential confounder. The following observations motivated the choice of gender: (i) the literature suggests that gender plays an important role in shaping the host response following trauma [2], making it a potential biological confounder affecting gene expression dynamics; and (ii) there is a statistically significant correlation between gender and ocMOF (p-value 0.01, Table 1 of main paper). In our study there are ~400 clinical variables to choose from and there is no principled way of selecting adjustment variables. Therefore, in order to account for the remaining confounders and uninteresting sources of variation without having to include additional

adjustment variables, we summarized the gene expression trajectories using the WPEC measure. The results of our principal component analysis (PCA) based analysis indicate that the WPEC measure helps alleviate the problem, allowing us to capture relevant clinical variation (see Text S3 for the technical details and Text S6 for the results).

Detailed Documentation of the R code

In an effort to make our data analysis fully reproducible by others [3], we have created an interactive suite of annotated scripts in the R statistical programming language that fully reproduces the results of this work from the raw data. This suite is available upon request from the authors and the codes are available as a supplementary dataset (see Supplementary Dataset S1). We ran these R codes on the R statistical software (version 2.8.0).

The *Dataset S1.zip* file contains the code for running the entire analysis in R statistical software (cran.r-project.org). We have organized the working directory into manageable and immediately apparent folders*.* The workflow of the entire analysis is ordered according to the subfolders within the *code* folder.

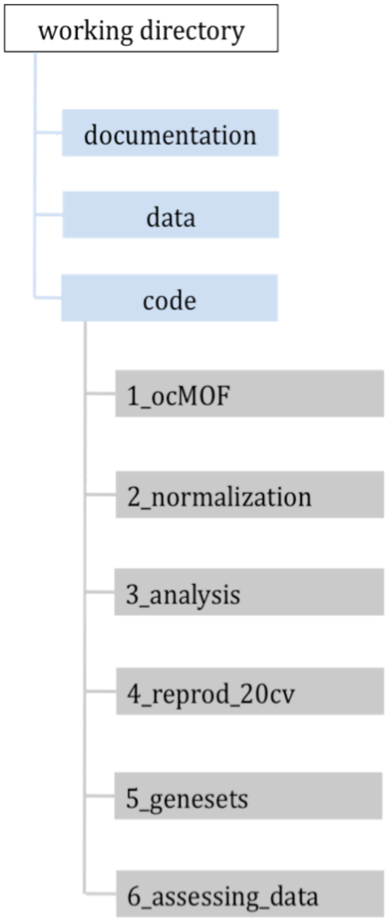

The code folder contains a *main.R* file that runs the overall analysis: loading data, executing other software and running sub-routines for performing the analysis. The R

codes are modular, allowing us to divide them into subfolders which are numbered according to their sequence in the *main.R*. The workflow in which *main.R* executes R codes corresponds to the following order of subfolders:

- **1_ocMOF**
  To get the ordered categorical MOF (ocMOF), the modified Marshall score trajectories from day 0 to 20 are used and missing entries are imputed by using the k-NN (k=10) approach, i.e. we used the R function *impute.knn* from R library *impute* with the argument *k* set to 10 (see *STEP 1: Longitudinal measure of developing MOF* for details). We performed hierarchical clustering on these scores to obtain five clusters, using the Euclidean distance as the dissimilarity metric and Ward as the agglomeration method by running *Getocmof.R*, i.e. we used the R function *hclust* with the argument *method* set to *"ward"*.
- **2_normalize**
  We normalize the microarrays separately according to batch with *dChip.exe* by running *DChipnormalization.R*, specifying the normalization settings with *dChipDefault.ini*. After normalizing the microarray data, we consolidate them with *Consolidatebatches.R* and compute the WPEC by running *ComputeWPEC.R*. To get the 20 cross-validation datasets, we run *Splitdata.R*.
- **3_analysis**
  We perform the adjusted Spearman analysis on WPEC and ocMOF, and obtain p-values by resampling the residuals without replacement (see *STEP 3: Adjusted Spearman rank-based correlation test* for details), i.e. we used the R function *adjSpearman* from *analysisfunctions.R* to perform the adjusted Spearman analysis and *sample* with the argument *replace* set to *FALSE* to obtain the resampled residuals.
- **4_reprod_20cv**
  We perform reproducibility analysis using 20 cross-validations by running *Reproducibility.R* (see *Assessment of Reproducibility* in the main paper).
- **5_genesets**
  We investigate the gene sets and modules identified and discussed in the paper by plotting the dominant trajectories and/or counting the number up/down regulated gene expression within each patient (*Boxplot_WPEC.R, Dominant_trajectory.R, ModuleTop3663.R*). We plot the mean log-expression of MHC-II and p38MAPK gene sets in the endotoxin data by using *Endotoxin_analysis.R*.
- **6_assessing_data**
  We assess the microarrays of the GLUE data, for example, the PCA analysis (*PCAarrayClinVarWPEC.R*, *PCAarrayClinVarMean.R*), heatmap of all 168 patients from hour 0 to 800 (*HeatmapAll.R*), exclusion of the first 12 hours (*First12hour.R*) and quality assessment of the microarrays (*DataQualArrayTime.R*). We obtain the clinical charateristics of the ocMOF subgroups (*ClinInfoTable.R*).

The *documentation* folder contains the *information.rtf* file, which gives an overview of the working directory and analysis framework, and provides instructions on how to run the R codes. For the user to navigate through the folders with minimal assistance, a *README.rtf* file is also available at the root of each folder, which describes the files and subfolders located in the folder.

The *data* folder contains the datasets used in the analysis, for example, the raw and normalized microarray files are in the *CEL* and *normarray* subfolders respectively. All of the data are freely available at www.gluegrant.org for registered researchers:

"Members who seek access to the human research data will have been granted from their home institution, institutional review board (IRB) approval to receive human research data in a manner consistent with the protection of confidentiality of the subjects" (www.gluegrant.org/glueadmin/register_consortium.jsp).

References

1. Troyanskaya O, Cantor M, Sherlock G, Brown P, Hastie T, et al. (2001) Missing value estimation methods for DNA microarrays. Bioinformatics 17: 520-525.
2. Choudhry MA, Bland KI, Chaudry IH (2007) Trauma and immune response--effect of gender differences. Injury 38: 1382-1391.
3. Baggerly K (2010) Disclose all data in publications. Nature 467: 401-401.
