# Supplementary material for: Dissecting Inflammatory Complications in Critically Injured Patients by Within-Patient Gene Expression Changes: A Longitudinal Clinical Genomics Study
Source: PLoS Med. 2011 Sep 13;8(9):e1001093. doi: 10.1371/journal.pmed.1001093 (PMC3172280; doi:10.1371/journal.pmed.1001093)
Supplement: Text S3 — Details on assessing WPEC robustness and reproducibility. (PDF) [file pmed.1001093.s036.pdf]

Text S3. Details on Assessing WPEC Robustness and Reproducibility

Assessing adequacy of WPEC

We sought to characterize WPEC by quantifying per-hour log-fold change. To do so, we regressed the log gene expression on time (hour) and extracted the linear slope. For genes with monotonically increasing or decreasing expression trajectories, the utility of our WPEC measure to discriminate is obvious. Our WPEC measure can also discriminate with more complex trajectories which may be reflective of the host genomic response to the initial injury and to the post-injury recovery. But inconsistent sampling timings across patients may have considerable effect, creating potential confounding. To increase the robustness and power of the analysis, it is useful to locate a time window such that the number of genes exhibiting reasonably monotonic trend increases.

For patients with enough time points (i.e. 3 or more time points), we computed the difference of WPEC (hour 12 to 250) and WPEC (hour 0 to 250) to form the DWPEC matrix. We used the one sample t-test to identify the set of probesets with significantly non-zero mean DWPEC. To get an overall goodness-of-fit between the loess fit (i.e. nonlinear trend permitted) and linear fit, we computed the mean-square difference (MSD) between the average trajectory and average linear trajectory of each probeset at various time windows: 0-250 hours, 4-250 hours, 8-250 hours and 12-250 hours. The boxplots of the MSDs for the 5000 most non-significant (ns) and significant (sig) probesets are used to evaluate the adequacy in using the slope to estimate WPEC (see Text S6 for the results).

Principal component analysis (PCA) based analysis

Besides WPEC, another summary measure that can be extracted from a longitudinal expression trajectory and be used in the adjusted Spearman analysis is the mean expression. However, this summary measure conflates the baseline expression and WPEC, which could potentially introduce spurious signal and/or weaken the actual signal. To gain better understanding of the robustness aspects of these measures, we performed an additional PCA based analysis. Note that the ocMOF outcome was derived prior to this analysis.

We performed PCA on the WPEC matrix (hour 12 to 250) and the mean expression matrix (hour 12 to 250); see Text S6 for the results. To determine the number of components, we used the scree plot. To model each clinical variable with PCs as explanatory variables, we used multiple linear regression and multinomial regression for continuous and categorical clinical variables respectively. If the continuous clinical variable contained more than five unique values, then it was modeled as a quantitative variable by using a robust, rank based regression model. Otherwise, we modeled the clinical variable as a categorical variable. To make the linear regression model rank based, we mapped the quantiles of the continuous variables to the corresponding quantiles of a normal distribution with mean equals 0 and variance equals 1.

Cross-validation for assessing statistical reproducibility

We utilized 20 cross-validations to establish reproducibility of these statistical associations. For each cross-validation, we randomly divided patients into similar size “discovery” and “validation” subsets while maintaining similar ocMOF distributions to avoid sampling bias. The entire analysis pipeline, including normalization, was performed separately between the discovery and validation data sets.
