# Supplementary material for: Dissecting Inflammatory Complications in Critically Injured Patients by Within-Patient Gene Expression Changes: A Longitudinal Clinical Genomics Study
Source: PLoS Med. 2011 Sep 13;8(9):e1001093. doi: 10.1371/journal.pmed.1001093 (PMC3172280; doi:10.1371/journal.pmed.1001093)
Supplement: Text S4 — Details on module, pathway, and gene set analysis. (PDF) [file pmed.1001093.s037.pdf]

Text S4. Details on Module, Pathway, and Gene Set Analysis

We used DAVID (Database for Annotation, Visualization, and Integrated Discovery)[1] with its default options and the "highest" classification stringency cut-off to obtain functionally related gene groups. DAVID uses a set of fuzzy classification algorithms to group genes based on their co-occurrences in annotation terms and ranks the gene groups using an internal (EASE) score [2]. We used Ingenuity Pathway Analysis (Ingenuity® Systems, www.ingenuity.com) to identify the top canonical pathways associated with a list of probesets, which uses the Fisher's exact test to ascertain enrichment.

For the module analysis, we used DAVID to classify 1256 of the 3663 probesets into 54 functionally related gene sets. For each gene set, we obtained five dominant expression trajectories corresponding to five ocMOF subgroups. While computing these dominant trajectories, we only included probesets with identical unadjusted and adjusted Spearman correlation signs to ensure robustness, and rescaled their gene expression to have a positive ocMOF-WPEC correlation. We used the average Pearson correlation of dominant trajectories across ocMOF subgroups as the similarity metric. If the signs of these Pearson correlations were inconsistent across ocMOF subgroups, then the similarity metric was set to zero (no similarity). Then, we used Ward as the agglomeration method and performed hierarchical clustering to obtain five modules (see Supp. Fig. 8). For each module, the five dominant trajectories were obtained by averaging the corresponding gene sets trajectories (see Supp. Fig. 9-13).

To obtain the five dominant expression trajectories for a gene set, we first removed patient-probeset specific effects by standardizing the gene expression values (scaling each patient using the sample standard deviation of all of its probesets and separately mean-centering each scaled probeset), and subsequently fitted a loess curve to each ocMOF subgroup. These five dominant trajectories were aligned to a common initial reference.

Ingenuity Pathway Analysis (IPA)
We upload a list of probesets (for example, the top 500 probesets) and perform a *Core Analysis* with the default settings in IPA. The *Canonical Pathway Analysis* in IPA associates the probesets with the canonical pathways in Ingenuity's Knowledge Base and returns two measures of association: (1) a ratio of the number of genes from the list that maps to the pathway divided by the total number of genes that map to the same pathway, and (2) a p-value of the Fisher's exact test. To identify significant gene sets that are the top five canonicial pathways from the five modules, we apply Bonferroni correction (i.e. $p\text{-value} < 0.05/25 = 0.002$) to the p-values in order to maintain the type I error at 5%.

For the top canonical pathways (i.e. gene sets) obtained from the top 500 probesets, we kept probesets with consistent empirical and biological relationships. The biological relationships between genes in a canonical pathway are available from *My Pathways* in IPA (which we referred to as IPA pathway). Using the biological relationships from IPA pathway and the top 50 probesets in the canonical pathway as references, we derive a trend between WPEC and ocMOF that is biologically driven for all genes in the same pathway. If this trend is consistent with the one computed from the data, the probeset is retained and used to compute the dominant trajectory. The IPA pathway provides a graphical representation of the biological

relationships between genes in a canonical pathway, where nodes represent genes and edges represent the biological relationships. Each edge is supported by at least one reference from the literature, a textbook, or canonical information stored in the Ingenuity Pathways Knowledge Base, providing us a relationship summary between genes.

References

1. Dennis G, Sherman BT, Hosack DA, Yang J, Gao W, et al. (2003) DAVID: Database for Annotation, Visualization, and Integrated Discovery. Genome Biol 4: P3.
2. Huang DW, Sherman BT, Tan Q, Collins JR, Alvord WG, et al. (2007) The DAVID Gene Functional Classification Tool: a novel biological module-centric algorithm to functionally analyze large gene lists. Genome Biol 8: R183.
