# Supplementary material for: Dissecting Inflammatory Complications in Critically Injured Patients by Within-Patient Gene Expression Changes: A Longitudinal Clinical Genomics Study
Source: PLoS Med. 2011 Sep 13;8(9):e1001093. doi: 10.1371/journal.pmed.1001093 (PMC3172280; doi:10.1371/journal.pmed.1001093)
Supplement: Text S5 — Marshall MOF-derived clinical outcomes. (PDF) [file pmed.1001093.s038.pdf]

Text S5. Marshall MOF-Derived Clinical Outcomes

Five clusters/subgroups were obtained by clustering the modified Marshall scores with missing scores imputed via k-NN (Supp. Fig. 3). Using the mean observed modified Marshall score trajectory for each subgroup from day eight onwards, we obtained the following ranking of subgroups (with increasing order of severity in clinical outcome): *ocMOF i*, *ocMOF ii*, *ocMOF iii*, *ocMOF iv* and *ocMOF v* (Supp. Fig. 4), containing 68, 32, 47, 16 and 5 patients respectively. Despite *ocMOF v* being the only subgroup with all its patients experiencing death (mean death day since injury is 12 days, ranging from 8 to 24 days), which suggests that *ocMOF v* is the subgroup with the worst clinical outcome; however, its first seven days mean observed MOF trajectory is very similar to that for *ocMOF iii*, suggesting the modified Marshall score alone is not very effective in discriminating post-injury MOF. The only other group with patients experiencing death is *ocMOF iv* (2 out of 16 deaths, 12 % mortality rate, the death days since injury are 11 and 20). For the four subgroups, *ocMOF i* to *iv*, the same ranking was observed for several other clinical variables. From Fig. 3 of main paper we observed that *ocMOF i* had the lowest median days for hospital stay/death and the median increased as we progressed from *ocMOF i* to *iv*. To adjust for the total number of days for hospital stay/death, we computed the proportion of ICU free days and ICU ventilation free days among the total number of days for hospital stay/death. For these variables, *ocMOF i* had the highest proportion and the median decreased as we progressed from *ocMOF i* to *v*. From Table 1 of main paper we observed that *ocMOF i* had the lowest percentage of patients with ventilator associated pneumonia, non-infectious complications, surgical site infections, nosocomial infections and ICU tracheostomy, and these percentages increased as we progressed from *ocMOF i* to *iv*. The high and early mortality experienced by patients with *ocMOF v* may explain the lower percentage values than *ocMOF iv* in some of the clinical variables.

If we define the incidence of MOF as the occurence of the modified Marshall score exceeding the value six, then the percentage of patients experiencing MOF increased from 0% for *ocMOF i* to 100% for both *ocMOF iv* and *v* as we progressed from *ocMOF i* to *v*. Similarly, the percentage of patients experiencing death within 28 days increased from 0% for *ocMOF i*, *ii* and *iii* to 100% for *ocMOF v* as we progressed from *ocMOF i* to *v*. We also performed the Spearman correlation test or the Deviance test as appropriate, and found statistically significant association (p-value<0.05) as well as monotonic trend between ocMOF and the above mentioned variables (Fig. 3 and Table 1 of main paper). While performing these tests, ocMOF was treated as a numerical variable (i.e., *ocMOF i* to ocMOF *v* taking the numerical values from 1 to 5, respectively).
