# Supplementary material for: Dissecting Inflammatory Complications in Critically Injured Patients by Within-Patient Gene Expression Changes: A Longitudinal Clinical Genomics Study
Source: PLoS Med. 2011 Sep 13;8(9):e1001093. doi: 10.1371/journal.pmed.1001093 (PMC3172280; doi:10.1371/journal.pmed.1001093)
Supplement: Text S6 — Results on assessing WPEC robustness. (PDF) [file pmed.1001093.s039.pdf]

Text S6. Results on Assessing WPEC Robustness

Assessing adequacy of WPEC

From the one sample t-test on DWPEC, we estimated that 52% of the probesets were either flat or had reasonably monotonic trajectories throughout hour 0 to 250. Supp. Fig. 5 plots the within-patient trajectories (gray lines) for a non-significant (i.e., zero mean DWPEC) and a significant probeset. The population average trajectory estimated from loess smoothing (black lines) and the population average linear trajectory (red lines) are also shown. For the significant probeset, after excluding expression <12 hours, the average trajectory seemed reasonably monotonic. Supp. Fig. 6 shows the MSDs of the 5000 most non-significant (ns) and significant (sig) probesets as we progressively exclude the early hours. At the cut-off of hour 12, the MSD drops considerably and we are still able to retain 126 patients with at least 3 time points between hour 12 to 250 for our final analysis.

The WPEC measure robustly captures relevant clinical variation

We performed PCA on the WPEC matrix (hour 12 to 250) and obtained eight principal components (PCs) explaining 31% of total variation (see Supp. Fig. 7a for the scree plot). We found that most of the top 10 clinical variables associated with the 8 PCs are related to Marshall or Denver scores (Supp. Table 1). Similarly, we performed PCA on the mean expression matrix (hour 12 to 250) and obtained eight PCs explaining 62% of total variaton (see Supp. Fig. 7b for the scree plot). Although most of the top ten clinical variables are related to MOF, two of them, sampling phase and trauma center, are time-independent and unrelated to trauma response (Supp. Table 2). Whereas, for WPEC, sampling phase was ranked 32 (p-value = 3.5×10^{-3}, q-value = 9.6×10^{-3}, McFadden's pseudo R^2=0.14) and trauma center was ranked 165 (p-value = 1.3×10^{-1}, q-value = 7.3×10^{-2}, McFadden's pseudo R^2=0.17). This suggests that the mean expression is affected by patient heterogeneity and confounded by other factors, whereas WPEC is more robust. For mean expression, ocMOF was ranked 46 (p-value = 9.0×10^{-5}, q-value = 1.1×10^{-4}, McFadden's pseudo R^2=0.20). We also analyzed the estimated gene expression at hour 12 (baseline) and noticed similar problems as the mean expression. Furthermore, we also repeated this analysis for a range of number of prinicipal components, (from 4 to 20), and these results also indicated that WPEC is less suspectible to confounders.
