# Supplementary material for: Dissecting Inflammatory Complications in Critically Injured Patients by Within-Patient Gene Expression Changes: A Longitudinal Clinical Genomics Study
Source: PLoS Med. 2011 Sep 13;8(9):e1001093. doi: 10.1371/journal.pmed.1001093 (PMC3172280; doi:10.1371/journal.pmed.1001093)
Supplement: Text S7 — Key pathways associated with trauma outcomes. (PDF) [file pmed.1001093.s040.pdf]

Text S7. Key Pathways Associated with Trauma Outcomes

Antigen presentation pathway

Using DAVID we obtained 16 probesets to represent the MHC-II gene set. The gene names from DAVID (Supp. Table 7) were used to map the probesets to their corresponding genes: HLA-DMB, HLA-DPA1, HLA-DPB1, HLA-DQA1, HLA-DRA, HLA-DRB1, HLA-DRB3, HLA-DRB4, LOC100294318, and LOC100133678.

p38 MAPK signaling pathway

Using Ingenuity Pathway Analysis (IPA) we obtained an additional 11 probesets that were in the top 500 and involved in p38MAPK (Supp. Fig. 17). We kept probesets with correlations between WPEC and ocMOF that were consistent with those identified by the IPA pathway by using the top 50 probesets (MAPK14, IRAK2 and TIFA) to infer the overall trend between WPEC and ocMOF in the p38 MAPK signaling pathway. Five of them had correlations that were consistent with those identified by IPA and they matched to the following genes: CREB5, IL1R1, IL1RN, IRAK3 and MAP2K6. Altogether 12 probesets (representing 8 genes) were used to investigate the effect of trauma on p38MAPK. The approach we took to identify the genes and probesets representing this pathway was also applied to the other canonical pathways discussed below.

The other canonical pathways

We also analyzed the gene expression trajectories of the other three canonical pathways: Toll-like receptor (TLR) signaling (Supp. Fig. 19 and 20), Interleukin(IL)-6 signaling (Supp. Fig. 21 and 22) and Production of nitric oxide and reactive oxygen species in macrophages (Supp. Fig. 23 and 24). Note that dendritic cell maturation contain genes from MHC-II and p38MAPK, which were shown above (Supp. Fig. 16 and 18). We applied the same approach as used for the p38 MAPK signaling pathway to identify genes in the other three canonical pathways. From the analysis, we observed that the trend of the dominant expression trajectories are similar to p38MAPK. These results are consistent with TLR expression being up-regulated in patients with sepsis [1,2], IL-6 being up-regulated in patients experiencing septic shock or death [3], and reactive oxygen species and nitric oxide produced by macrophages having an important role in tissue injury after ischemia and reperfusion [4]. However, as a caveat to the canonical pathway "Production of Nitric Oxide and Reactive Oxygen Species in Macrophages", we note: (i) the probesets representing this pathway are generic for inflammation (for example, NFkB, IkB, JAK, P13K, p38MAPK) and (ii) it is not clear whether related products, such as iNOS and NADPH, are up-regulated.

Endotoxin Data

To corroborate the dominant trajectories of MHC-II and p38MAPK among patients with fast and uncomplicated recovery (i.e. *ocMO*F *i* and *ii*), we used a controlled endotoxin experiment data set. The data set consisted of eight human subjects. Endotoxin was administered to four of the subjects while a placebo was administered to the rest. Blood samples were collected before infusion and at 2, 4, 6, 9, and 24 hours afterward. The protocols for the collection of blood samples and array hybridization are described elsewhere[5]. Among subjects who were administered

the placebo, their mean log-expression (black lines) were flat for both MHC-II and p38MAPK (Supp. Fig. 25). Among subjects who were administered the endotoxin, we observed a trough and peak in their mean log-expression (red lines) of MHC-II and p38MAPK respectively, and a similarity between their mean log-expression after hour 5 and the dominant expression trajectory of *ocMOF i* and *ii*, which increased and decreased over time for MHC-II and p38MAPK respectively.

References

1. Harter L, Mica L, Stocker R, Trentz O, Keel M (2004) Increased expression of toll-like receptor-2 and -4 on leukocytes from patients with sepsis. Shock 22: 403-409.
2. Armstrong L, Medford ARL, Hunter KJ, Uppington KM, Millar AB (2004) Differential expression of Toll-like receptor (TLR)-2 and TLR-4 on monocytes in human sepsis. Clinical and Experimental Immunology 136: 312-319.
3. Presterl E, Staudinger T, Pettermann M, Lassnigg A, Burgmann H, et al. (1997) Cytokine profile and correlation to the APACHE III and MPM II scores in patients with sepsis. American Journal of Respiratory and Critical Care Medicine 156: 825-832.
4. Mannick JA, Rodrick ML, Lederer JA (2001) The immunologic response to injury. Journal of the American College of Surgeons 193: 237-244.
5. Calvano SE, Xiao WZ, Richards DR, Felciano RM, Baker HV, et al. (2005) A network-based analysis of systemic inflammation in humans. Nature 437: 1032-1037.
