# Supplementary material for: Dissecting Inflammatory Complications in Critically Injured Patients by Within-Patient Gene Expression Changes: A Longitudinal Clinical Genomics Study
Source: PLoS Med. 2011 Sep 13;8(9):e1001093. doi: 10.1371/journal.pmed.1001093 (PMC3172280; doi:10.1371/journal.pmed.1001093)
Supplement: Text S8 — p38 MAPK and MHC-II as biomarkers for SIRS and CARS. (PDF) [file pmed.1001093.s041.pdf]

Text S8. p38 MAPK and MHC-II as Biomarkers for SIRS and CARS

To discover probesets with similar WPEC pattern as the 16 MHC-II probesets, we computed the Spearman correlation between the probeset and each of the 16 MHC-II probesets and took the mean. We kept probesets with consistent signs across all 16 correlation coefficients. We selected 500 probesets with the largest absolute average correlation and used Ingenuity to identify the top six canonical pathways in these 500 probesets (p-values, 4.0×10^{-5}-8.1×10^{-4}): B cell development, CTLA4 signaling in cytoxic T Lymphocytes, Calcium-induced T Lymphocyte Apoptosis, Regulation of eIF4 and p70s6K signaling, Primary immunodeficiency signaling and EIF2 signaling.

Two of the pathways are associated with apoptosis and immunodeficiency, which are characteristics of CARS [1]. Since IL-6 levels represent the net effect of IL-1 and tumor necrosis factor (TNF) [2], both p38MAPK (Supp. Fig. 18) and IL-6 signaling (Supp. Fig. 22) having similar dominant trajectories suggest that p38MAPK is a potential biomarker for SIRS. It has been posited that MOF is an outcome of an inappropriate generalized inflammatory response as well as a failure of adaptive immunity, and increased susceptibility to secondary infections [3]. An interesting scenario to explore in future studies is that the increase in p38MAPK expression seen here after severe trauma reflect the development of SIRS while the persistent decrease in MHC-II expression reflect the development of CARS.

References

1. Adib-Conquy M, Cavaillon JM (2009) Compensatory anti-inflammatory response syndrome. Thrombosis and Haemostasis 101: 36-47.
2. Dinarello CA (1997) Proinflammatory and anti-inflammatory cytokines as mediators in the pathogenesis of septic shock. Chest 112: 321S-329S.
3. Remick DG (2007) Pathophysiology of sepsis. Am J Pathol 170: 1435-1444.
